# Genomic admixture tracks pulses of economic activity over 2,000 years in the Indian Ocean trading network

**DOI:** 10.1038/s41598-017-03204-y

**Published:** 2017-06-07

**Authors:** Nicolas Brucato, Pradiptajati Kusuma, Philippe Beaujard, Herawati Sudoyo, Murray P. Cox, François-Xavier Ricaut

**Affiliations:** 1 Evolutionary Medicine Group, Laboratoire d’Anthropologie Moléculaire et Imagerie de Synthèse UMR 5288 CNRS, Université Toulouse III, Université de Toulouse, Toulouse, France; 2 Genome Diversity and Diseases Laboratory, Eijkman Institute for Molecular Biology, Jakarta, Indonesia; 3 Institut des Mondes Africains, UMR 8171 CNRS UMR 243 IRD Paris, France; 4 Department of Medical Biology, Faculty of Medicine, University of Indonesia, Jakarta, Indonesia; 5 Statistics and Bioinformatics Group, Institute of Fundamental Sciences, Massey University, Palmerston North, New Zealand

## Abstract

The Indian Ocean has long been a hub of interacting human populations. Following land- and sea-based routes, trade drove cultural contacts between far-distant ethnic groups in Asia, India, the Middle East and Africa, creating one of the world’s first proto-globalized environments. However, the extent to which population mixing was mediated by trade is poorly understood. Reconstructing admixture times from genomic data in 3,006 individuals from 187 regional populations reveals a close association between bouts of human migration and trade volumes during the last 2,000 years across the Indian Ocean trading system. Temporal oscillations in trading activity match phases of contraction and expansion in migration, with high water marks following the expansion of the Silk Roads in the 5^th^ century AD, the rise of maritime routes in the 11^th^ century and a drastic restructuring of the trade network following the arrival of Europeans in the 16^th^ century. The economic fluxes of the Indian Ocean trade network therefore directly shaped exchanges of genes, in addition to goods and concepts.

## Introduction

For more than 2,000 years, the Indian Ocean rim has been an area of intense interaction between African, Middle Eastern and Asian populations, driven by a strong tradition of wealthy maritime and land-based trading routes^1, 2^. The political unification of large territories in the third century BCE (Before Current Era) opened up new trading routes, most famously the Silk Roads and the maritime network along the coasts of the Indian Ocean^3, 4^. These in turn triggered sustained interactions between major geopolitical poles, including states in China, India, Indonesia, Arabia and East Africa^1, 5, 6^. Trade was both diverse and intense, benefiting from specialized local production, such as cotton and beads from India, gold from East Africa, spices from the Malacca city-states, incense from Yemen and silk from China^1^. With population growth and technical advances, notably in agriculture, the first century CE saw a major intensification in the movements of goods and people^3^. The development of new sailing techniques, particularly during the 11^th^ century CE, enabled movements over very long distance. Indonesian traders reached as far as East Africa and the Swahili city-states^7^; Arab sailors installed trading posts on Madagascar and the west coast of India^1^; and Chinese traded across Island Southeast Asia and East India. Far from competing, the various maritime and terrestrial routes created an intertwined and dense network that rapidly diffused goods, but also knowledge, beliefs and values, proving a unifying force across a diverse set of partners^1, 3, 6^. New urban spaces acted as hubs to the flow of trade and culture, often growing into cosmopolitan cities with large immigrant populations, such as in Baghdad and Zanzibar^8, 9^. The intensity, stability and speed of these trading connections formed a large world-system, a precursor to the heavily globalized societies of today^10^. Yet whether Indian Ocean trade directly shaped the genetics of modern populations is less well understood.

## Results

Genome-wide genetic variation in 3,006 individuals from 187 regional populations was used to build a picture of gene flow around the Indian Ocean rim over the past 2,000 years (Supplementary Table 1). The genetic landscape of the Indian Ocean rim today, as characterized by ADMIXTURE^11^ and EEMS^12^, is a structured space with distinct regional genetic ancestries allowing the fine-scale reconstruction of historical human migrations mediating gene flow (Supplementary Figures 1–6). Long corridors of genetic similarity can be seen along the coasts of East Africa, South Asia and the rim of the China Sea, but also strong genetic barriers such as one observed between South Asia and East Africa (Supplementary Figure 1). Despite these genetic barriers remarkable instance of gene flow can be identified such as the Asian gene flow to Madagascar (Supplementary Figures 4 and 5)^13, 14^.

**Figure 1:**
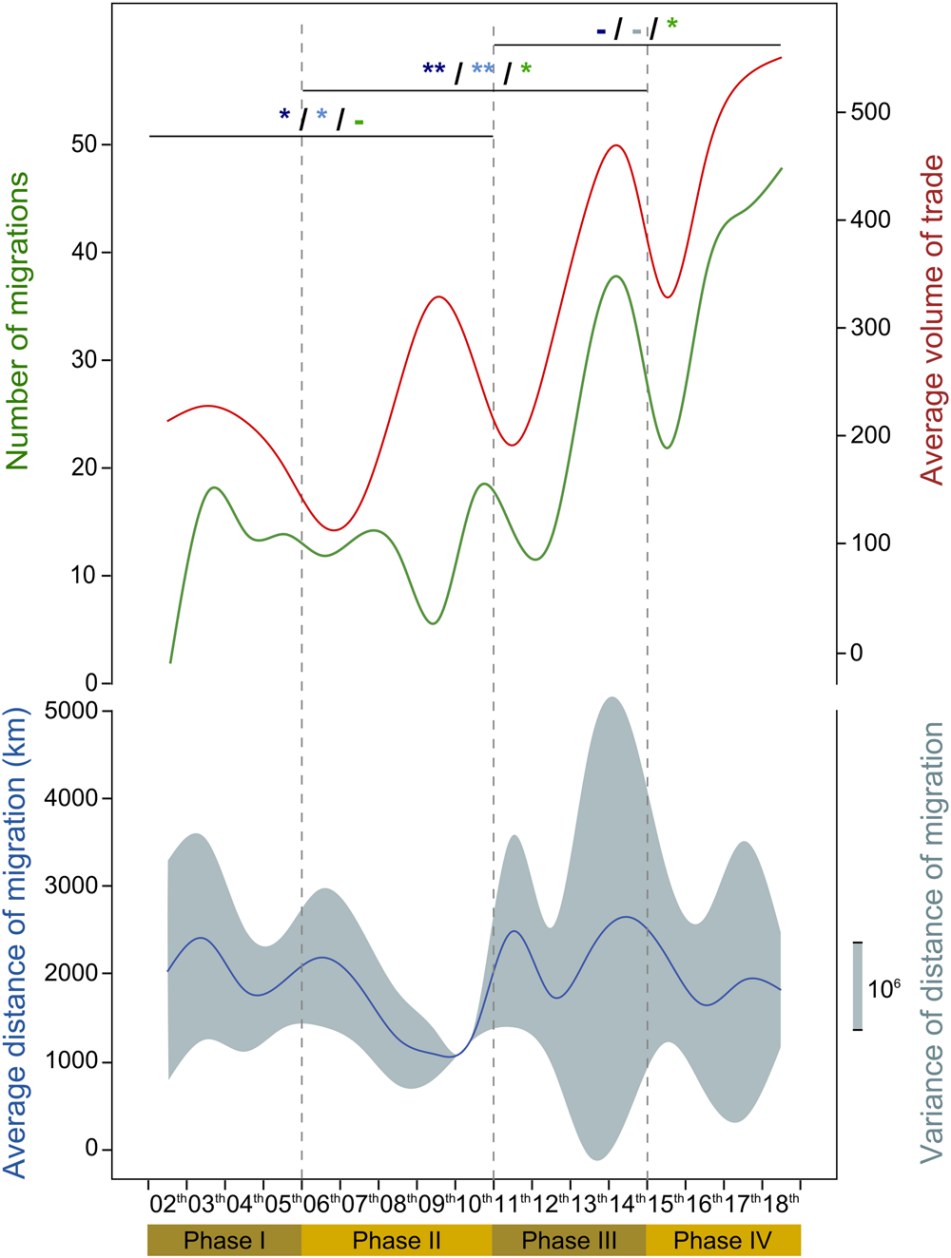
Association between human migration and trade volumes through time. The green line shows the number of migration events estimated by GLOBETROTTER^15^ per century. The red line shows the average volume of trade per century adapted from data detailed by Beaujard^9^. The blue line shows the average distance of migration per century, with light blue shading showing the variance. Dashed vertical lines mark the four trade phases^9^. Horizontal bars represent t-tests between successive phases with significance values shown for the variance of migration distances (light blue), the average migration distance (dark blue) and the number of migration events (green): **P < 0.01; *P < 0.05; -: not significant.

To determine whether trade drove significant bouts of population mixing, the temporal pulses of genetic dispersal around the Indian Ocean were estimated with GLOBETROTTER^15^ and MALDER^16^ (Supplementary Tables 2 and 3). Consistent with the idea that trading activities stimulate biological contacts, both analyses indicate that migration is highly correlated with historical trading volumes^9^ (*r* ^*2*^ = 0.89, *P* = 0.00001; Fig. 1; Supplementary Tables 4, 5 and Supplementary Figure 7). This model is a significantly better fit to the data than a simple increase of the number of admixture events over time (*P* = 0.009; Supplementary Table 5), which might be expected due to the statistical bias of the software towards estimating more recent admixture events. While the overall intensity of trade and population interactions increased steadily over time, these activities instead directly track the periodicity of economic developments and recession^9^. Four major phases of trade have previously been described^9^, with intervening recessions not breaking the network but instead restructuring connections leading to a new dynamic^9^. These periods of increased trade are associated with bursts of human migration (*F* = 10.39, *P* = 0.0002; Fig. 1; Supplementary Table 6).

Despite technological advancements and the expansion of Indian Ocean trade, the average migration distance did not increase through time (*P* > 0.05; Fig. 1; Supplementary Table 5). However, migration distances did fluctuate, with periods of extreme migration followed by contractions in population movements, corresponding to the phases of trade (Fig. 1; *F*_*average*_ = 5.34, *P* = 0.01; *F*_*variance*_ = 4.33, *P* = 0.03; Supplementary Table 6).

**Figure 2:**
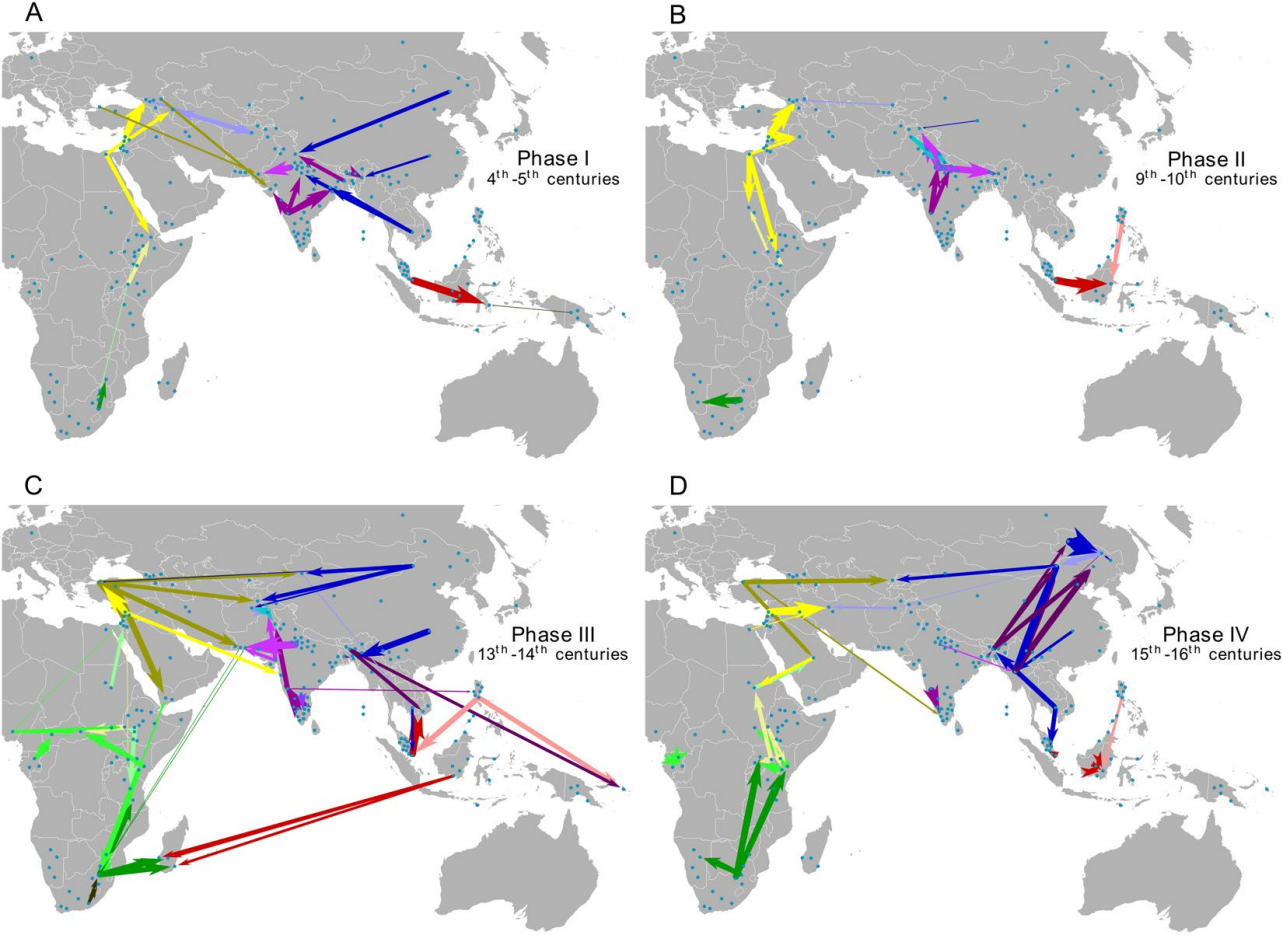
Maps showing gene flow during the four phases of Indian Ocean trade. Admixture events during two century periods are plotted for each phase: (**A**) Phase I, (**B**) Phase II, (**C**) Phase III and (**D**) the beginning of Phase IV. Arrow widths are proportional to the percentage of ancestry inherited from each source population. Colors are specific to each cluster, as defined by the fineSTRUCTURE^31^ results: green palette: sub-Saharan African Pygmies, Bantu, East African and Malagasy clusters; yellow palette: Middle East, Nilo-Saharan and Caucasus clusters; purple palette: Pakistan, India and Bengal clusters; blue palette: East and North Asian clusters; red palette: Indonesian clusters; brown and orange palettes: Negrito, Andaman and Papuan clusters. Blue dots illustrate the locations of sampled populations. Maps were generated using Global Mapper v.15. (http://www.bluemarblegeo.com/products/global-mapper.php).

The first phase (1^st^–5^th^ century) reflects the rise of the Silk Roads^1, 2^, dominated by terrestrial and coastal migrations from China to Arabia (Fig. 2A; Supplementary Table 2). Major gene flows are detected in the northern part of India corresponding to the influential zone of the Gupta Empire (330–550 CE), as previously identified^17^. This gene flow highlights long-distance interactions with Southeast Asia and China, which were the main actors for trade in items sold to West Eurasian and Middle East markets. At this period, Arab merchants were already dominating the Near East trading routes, whose influence can be seen by the numerous gene flows originating from the Arabian peninsula (Fig. 2A; Supplementary Table 2)^18, 19^.

The consolidation of the second *Pax Sinica* by the Chinese Tang Empire stimulated the Indian Ocean world-system to expand further^3, 4^ (Phase II; 6^th^–10^th^ century). This occurred in parallel with the spread of Islam by Arab merchants, marked by an intensification of gene flows from the Middle East to East Africa and Central Asia (Fig. 2B; Supplementary Table 2), as also reported in previous studies^19, 20^. Although causes of recessions are always multi-factorial, trade conditions likely declined at the end of Phase II due to the demographic collapse of urban centers in the Middle East, such as Baghdad following the fall of the Abbasid Caliphate in Arabia, and perhaps exacerbated by arid climatic conditions affecting agricultural production in Central Asia^3^. The end of Phase II is characterized by both a significant reduction in human migration and smaller migration distances (*P* < 0.05; Figs 1 and 2B; Supplementary Table 6).

Major technical improvements in sailing, such as the widespread adoption of the compass, likely triggered Phase III (11^th^–14^th^ century) with the appearance of new maritime routes^2^ (Fig. 2C; Supplementary Table 2). Hindu Malay Empires, such as Srivijaya and Mojopahit in Island Southeast Asia^3^, dominated this reinvigorated maritime trade, notably with Chinese and Indian Empires, as can be seen by numerous gene flows occurring between these areas (Fig. 2C; Supplementary Table 2). This state of domination was followed by Austronesian settlements in the Comoros and in Madagascar^21^, which we had previously established^13, 14^. We note that no other Austronesian gene flow to the western rim of the Indian Ocean was inferred by our analyses, suggesting a direct route of migration to Madagascar. Along with the development of the Swahili Corridor^22^, which can be inferred from the high density of gene flows in East Africa at that time, Arab merchants developed trading posts on the East African coast, to increase their access to gold and slaves. These slaves were deported to Arabia and South Asia, as shown by the South African Bantu gene flow into Yemen and South Pakistan^15, 23^. Finally, we also detected gene flow between Mongols and populations from Central Asia and Anatolia^24^, as well as Turkish gene flows to Middle Eastern groups, converging with the Mongol migration which started in 1206 with the reign of Genghis Khan and reached Arabia in 1258. These vast migrations participated in the unification of Indian Ocean trade partners at an unprecedented geographical scale.

However, the most drastic change occurred in the middle of the 14^th^ century (Fig. 2D; Supplementary Table 6). Outbreaks of plague in Asia, Africa and Europe combined with climatic changes led to a major demographic crisis^9^. This is reflected in major geopolitical restructuring, such as the fall of the Chinese Yuan Empire, which controlled the terrestrial Silk Roads, and the prohibition of trade between China and Southeast Asia, dictated by the Ming Empire in 1433 AD^3^. These events are mostly noticeable in our analyses by reduced gene flow in Island Southeast Asia and increased migration within China. An additional shock included the arrival of Europeans in the 16^th^ century, a major disruptive factor. This recession was followed by Phase IV (15^th^–16^th^ centuries to the present), with the Industrial Revolution driving another long period of strong international trade.

All of these trade spikes were paired with physical population movements, showing that the flow of goods and ideas was linked to the movements of the people who brought them. At least in the Indian Ocean, at a global scale, trade and migration were coupled forces, and the Indian Ocean trade network therefore provides an early example of globalization, showing connections between human trade and mobility that are still apparent around the world today.

## Methods

### Dataset

Our dataset is based on previously published studies of populations around the Indian Ocean rim (Supplementary Table 1). This dataset was built to trade-off large population diversity with the high number of overlapping SNPs, necessary for Identity-by-Descent (IBD) based methods, given the wide spectrum of genotyping platforms used by the scientific community. To avoid any statistical bias that could be introduced by a size effect of over-represented populations, we randomly selected a maximum of 25 individuals in each group, such that each population has sample size between 3 and 25. Quality controls were applied using Plink v1.9^25^ to filter for i) close relatives, using an IBD estimation with upper threshold of 0.25 (second degree relatives); ii) SNPs that failed the Hardy-Weinberg exact (HWE) test (P < 10^−6^) were excluded; iii) samples with a call rate <0.99 and displayed missing rates >0.05 across all samples in each population were excluded. After filtering, our dataset included a total of 3,006 individuals, genotyped for 215,335 SNPs, from 187 different populations located in Southeast Asia, South Asia, East Asia, Middle East, East Africa, South Africa, and Europe (Supplementary Table 1). All genotypes were phased together with SHAPEIT v2.r790^26^ using the 1000Genomes phased data^27^ as reference panel and the HapMap phase 2 genetic map^28^. The same dataset was in parallel pruned for Linkage Disequilibrium (LD; r^2^ < 0.2) with Plink v1.9^25^ resulting in an alternative dataset of 100,830 SNPs for specific analyses.

### Statistical Analyses

The genetic diversity of our dataset pruned for LD was first analyzed by EEMS v1^12^ to define genetic barriers and corridors. Using geographic coordinates (with the noticeable exception of HGDP CEU samples placed in Germany for graphical convenience) and a genetic dissimilarity matrix between populations, we set a map of the Indian Ocean rim defining a grid of 1,000 demes. Depending on their location, several populations may be included in one deme. 3 × 10^6^ MCMC iterations were run before checking for convergence of the MCMC chain. Plots were generated in R following the EEMS v1^12^ manual (Supplementary Figures 1 and 2). ADMIXTURE v1.23^11^ was used with default settings to decompose genetic ancestries of the pruned dataset. Ten iterations with randomized seeds were run and compiled with CLUMPAK v1^29^. We use the minimum average cross-validation value to define the most descriptive K components, and the major modes defined by CLUMPAK v1^29^ are reported. Plots were obtained using Genesis v.0.2.5^30^. The lowest cross-validation value was obtained for K = 29 (Supplementary Figures 4–6). Both of these analyses were used together to define the genetic diversity of our dataset.

Before estimating admixture scenarios, we defined clusters of populations. We first performed a fineSTRUCTURE v2.07^31^ analysis using the phased dataset to define genetic clusters^31^ (Supplementary Figure 3). This method detects shared IBD fragments between each pair of individuals, without self-copying, calculated with CHROMOPAINTER v2.0^31^ (default settings) to perform a model-based Bayesian clustering of genotypes. Mutational rates (Mu) and effective population size (Ne) were estimated with an Estimation-Maximization (EM) algorithm running in CHROMOPAINTER v2.0^31^, and was performed on all 22 autosomes for the entire dataset (10 iterations). The weighted average of these parameters, according to the SNP coverage of each chromosome and the number of individuals, was then used to compute the chromosome painting. Using fineSTRUCTURE v2.07^31^ with 2 × 10^6^ Markov-Chain-Monte-Carlo (MCMC) iterations, discarding the first 1 × 10^6^ iterations as “burn-in”, sampling from the posterior distribution every 10,000 iterations following the burn-in, a coancestry heat map and a dendrogram were inferred to visualize the number of clusters defined statistically that best describe the data.

This analysis defined clusters of populations that share a similar genetic history, so that populations in one cluster cannot be used as a parental group for another population in the same cluster. This criterion is critical to avoid statistical bias for the following analyses, notably GLOBETROTTER v2.0^15^. Population clusters were defined in two steps. From the fineSTRUCTURE v2.07^31^ results, each cluster was defined by: (i) high posterior probabilities given for the nodes of the population dendogram (>0.8); (ii) at least 100 individuals per cluster. This last criteria, although arbitrary, allowed us to define uniform clusters from statistically robust branches higher up in the tree, in a similar approach to that reported previously^31^. Subsequently an F_ST_ matrix was calculated with Eigensoft v5.0.2^32^ between populations within each cluster to define outliers with F_ST_ values greater than one standard deviation from the mean (Supplementary Table 7). Eight populations called as outliers could only be defined as ‘surrogates’ within their respective cluster (Supplementary Table 7). Those outliers were not analysed as a ‘target’ as they all show positive f3-statistics^33^ results and are known to have no recent history of admixture^33–37^ (Supplementary Table 7). After all criteria were applied, 22 clusters were defined (Supplementary Table 1 and Supplementary Figure 4).

To test different scenarios of admixture we performed GLOBETROTTER v2.0^15^ analyses for each population (excluding outliers) defining surrogates populations from all clusters but its own. Note that the numbers of populations within a given cluster is not correlated with the estimated dates of admixture (*P* = 0.95). The painted chromosomes obtained by CHROMOPAINTER v2.0^31^ for each population were used in GLOBETROTTER v2.0^15^ to estimate the ratios and dates of the potential admixture events that characterize them. Coancestry curves were estimated with and without standardization using a ‘NULL’ individual, and consistency for each estimated parameter was checked. 100 bootstrap resamplings were performed to estimate the p-value of the admixture events (considering the ‘NULL’ individual) and the 95% confidence interval for the obtained dates. The ‘best-guess’ scenario given by GLOBETROTTER v2.0^15^ was considered for each target population. Admixture events whose estimated 95% confidence intervals of dates between both ‘NULL0’ and’NULL1’ models do not overlap were subsequently reclassified as ‘uncertain’, as described by Hellenthal *et al*.^15^. To obtain a second estimate of potential admixture scenarios, we ran MALDER v1^16^, a modified version of the ALDER v1.3^16^ software to observe any multiple admixture events, using the parental populations defined by GLOBETROTTER v2.0^15^. Both analyses give highly correlated admixture dates (r^2^ = 0.65; *P* < 0.00001; Supplementary Figure 7). The estimated dates likely reflect the midpoint or end of noticeable admixture events rather than the exact date of migration (which could occur prior to any admixture). The number of migrations per century is equivalent to the cumulative number of parental populations, given by the ‘best-matching’ scenario in GLOBETROTTER v2.0^15^, and involved in each admixture event not defined as ‘uncertain’ (Supplementary Tables 3 and 4). Dates of admixture, given in generations, were converted to chronological time using a generation interval of 25 years.

In parallel with the admixture dates obtained from GLOBETROTTER^15^ and MALDER^16^, we computed geographical distances and historical trading volumes in order to perform correlation tests. Euclidian distances between a given target group, whose admixture scenario is not defined as ‘uncertain’ in GLOBETROTTER v2.0^15^, and each of its parental sources, given by the ‘best-matching’ scenario, were calculated using the great circle formula. We used the estimated value of 6,371 km for the Earth’s radius. Average volumes of trade per century were calculated from historical data^9^. Five measures of trade volume were taken for each century to obtain an average per century. This allows us to compare these data to the number of admixture events per century. However we note that this also smoothes the exact evolution of trade, as more precisely described in Beaujard^9^, as for example the drastic decrease observed at the end of Phase III^9^ which appears more progressive in our representation (Fig. 1). Therefore this measure does not reflect the exact evolution of trade volume but rather its overall trend across the centuries. Descriptive statistics of the distance of gene flow per century, correlation tests, t-tests and analyses of variance were computed with SPSS v20.0^38^. Curves were generated in SPSS v20.0^38^ using the spline interpolation. We performed correlation tests between our variables as we did not put any assumption on the dependence of one to another (for example, trade on the number of migrations). We performed an F-test to compare the models with time and volume of trade using SPSS v20.0^38^. When required, the Bonferroni multiple testing correction was applied. Networks were generated with Cytoscape v3.2.1^39^ and maps were generated using Global Mapper v.15. (http://www.bluemarblegeo.com/products/global-mapper.php).

## Electronic supplementary material

Supplementary File
