# Supplementary material for: Genomic admixture tracks pulses of economic activity over 2,000 years in the Indian Ocean trading network
Source: Sci Rep. 2017 Jun 7;7:2919. doi: 10.1038/s41598-017-03204-y (PMC5462752; doi:10.1038/s41598-017-03204-y)
Supplement: Supplementary file 1 — Supplementary File [file 41598_2017_3204_MOESM1_ESM.pdf]

SUPPLEMENTARY FILES

Genomic admixture tracks pulses of economic activity over 2,000 years in the Indian Ocean trading network

Nicolas Brucato[1], Pradiptajati Kusuma[1,2], Philippe Beaujard[3], Herawati Sudoyo[2,4], Murray P. Cox[5] and François-Xavier Ricaut[1].

[1] Evolutionary Medicine Group, Laboratoire d'Anthropologie Moléculaire et Imagerie de Synthèse UMR 5288 CNRS, Université Toulouse III, Université de Toulouse. France.
[2] Genome Diversity and Diseases Laboratory, Eijkman Institute for Molecular Biology, Jakarta, Indonesia.
[3] Institut des Mondes Africains, UMR 8171 CNRS UMR 243 IRD, Paris, France.
[4] Department of Medical Biology, Faculty of Medicine, University of Indonesia, Jakarta, Indonesia.
[5] Statistics and Bioinformatics Group, Institute of Fundamental Sciences, Massey University, Palmerston North, New Zealand.

Supplementary Table 1 – Database of the 3,006 individuals used in the present study. The first column specifies the cluster label used with fineSTRUCTURE[1] analysis.

Cluster	Country	Population	N	Reference
17	CAR	Biaka Pygmy	20	[2]
17	Gabon	Baka	16	[3]
17	Gabon	Bongo	25	[3]
17	DR Congo	Mbuti Pygmy	13	[2]
17	Uganda	Batwa	25	[3]
17	Tanzania	Sandawe	25	[4]
18	Nigeria	Yoruba	25	[4]
18	Gabon	Nzebi	20	[3]
18	South Africa	SW Bantu	12	[5]
18	Uganda	Bakiga	25	[3]
18	Kenya	Luhya	25	[4]
18	Kenya	Bantu Kenya	10	[2]
19	South Africa	SE Bantu	20	[5]
19	South Africa	Bantu Soweto	25	[6]
19	South Africa	BantuSA	8	[2]
16	Malagasy	Mikea	21	[7]
16	Malagasy	Temoro	24	[7]
16	Malagasy	Vezo	24	[7]
16	South Africa	Coloured Wellington	20	[5]
16	South Africa	Coloured Colesberg	20	[5]
20	Tanzania	San	5	[4]
20	South Africa/Namibia	Juhoansi	18	[5]
20	South Africa	Xun	19	[5]
20	South Africa	Khwe	17	[5]
20	South Africa/Botswana	GuiGhanaKgal	15	[5]
20	South Africa	San	25	[4]
20	South Africa/Namibia	Nama	20	[5]
20	South Africa	Khomani	25	[5]
20	South Africa	Karretjie	20	[5]
22	Ethiopia	Ari Blacksmith	17	[8]
22	Ethiopia	Ari Cultivator	24	[8]
22	Tanzania	Hadza	6	[4]
22	Ethiopia	Gumuz	19	[8]
22	Ethiopia	Anuak	23	[8]
22	Sudan	Sudanese	24	[8]
22	Kenya	Turkana	8	[9]
21	Somalia	Somali	25	[8]
21	Ethiopia	Somali	17	[8]
21	Kenya	Samburu	10	[9]
21	Ethiopia	Wolayta	8	[8]
21	Ethiopia	Oromo	25	[10]
21	Ethiopia	Afar	12	[8]

21	Ethiopia	Ethiopian Jews	13	10
21	Ethiopia	Amhara	25	10
21	Ethiopia	Ethiopians	5	10
21	Ethiopia	Tygray	21	8
21	Sudan	Nubian	14	9
21	Sudan	Arab	16	9
14	Yemen	Yemenite Jews	15	10
14	Saudi Arabia	Saudi	20	10
14	Israel	Samaritians	3	10
14	Israel	Bedouin	25	2
14	Israel	Palestinian	25	2
14	Jordania	Jordanian	20	2
14	Lebanon	Lebanese	7	10
14	Syria	Syrians	16	10
14	Egypt	Egyptian	12	4
14	Yemen	Yemeni	10	2
15	Israel	Druze	25	2
15	Iraq	Iraqi Jews	11	10
15	Turkey	Sephardic Jews	10	10
15	Georgia	Georgian Jews	4	10
15	Iran	Iranian Jews	4	10
15	Azerbaijan	Azerbaijani Jews	8	10
15	Armenia	Armenian	19	2
15	Georgia	Georgian	10	2
15	Turkey	Turks	17	10
15	Georgia	Georgians	10	10
15	Iran	Iranian	20	2
15	USA	European-descendants from Utah	25	2
15	Russia	Lezgin	9	10
13	Pakistan	Makrani	25	2
13	Pakistan	Balochi	24	2
13	Pakistan	Brahui	25	2
13	Pakistan	Pathan	22	2
13	Pakistan	Sindhi	24	2
13	India	Khatri	19	11
13	Pakistan	Burusho	25	2
13	Pakistan	Kalash	23	2
12	India	Mumbai Jews	4	10
12	India	Cochin Jews	4	10
12	India	Punjabi	25	2
12	India	Brahmin	25	12
12	India	Gujarat Brahmin	19	11
12	India	Brahmins from Uttar Pradesh	8	13
12	India	Iyer	20	11
12	India	West Bengal Brahmin	18	11
12	India	Kshatriya	7	14

12	Pakistan	Punjabi	8	2
12	India	Muslim	5	13
12	India	Gujarati	25	2
12	India	Dharkar	12	13
12	India	Uttar Pradesh Scheduled Caste	5	13
12	India	Kanjars	8	13
11	India	Chamar	10	13
11	India	Kol	17	13
11	India	North Kannadi	9	10
11	India	Palian	20	11
11	India	Hakkipikki	4	13
11	India	Sakilli	4	10
11	India	Chenchus	4	13
11	India	Velama	10	12
11	India	Maratha	7	11
11	India	Piramalai Kallar	8	13
11	Sri Lanka	Sri Lanka UK	25	2
11	Bangladesh	Bengali	7	2
11	India	Kurumba	4	13
11	India	Bengali	25	2
11	India	Dusadh	10	13
10	India	Birhor	17	15
10	India	Irula	20	12
10	India	Kadar	20	11
10	India	Korva	18	11
10	India	Santal	20	11
10	India	Ho	23	16
9	India	Bonda	4	16
9	India	Gond	24	13
9	India	Pulliyar	5	13
9	India	Paniya	22	10
9	India	Jarawa	19	11
9	India	Onge	17	12
8	India	Tharu	22	12
8	India	Manipuri Brahmin	20	11
8	Myanmar	Burmese	25	16
8	India	Jamatia	18	11
8	India	Tripuri	19	11
8	India	Garo	4	16
8	India	Khasi	3	16
1	Indonesia	Ma'anyan	25	17
1	Indonesia	SK Dayak	25	17
1	Indonesia	Banjar	16	18
1	Malaysia	Malay	25	19
2	Indonesia	Murut	23	20
2	Indonesia	Dusun	22	20

2	Philippines	Casigurans	6	[21]
2	Philippines	Zambales	6	[21]
2	Phillippines	Filipino	16	[20]
2	Phillippines	Igorot	22	[20]
2	Indonesia	Sumba	25	[22]
2	Indonesia	Bajo	25	[7]
2	Indonesia	Mandar	24	Pierron et al., unpublished
3	Indonesia	Lebbo	18	[7]
3	Malaysia	Jakun	11	[23]
3	Malaysia	Temuan	13	[23]
3	Malaysia	CheWong	11	[23]
3	Philippines	Tagbanua	13	[21]
3	Philippines	Agta	3	[21]
3	Philippines	Batak	25	[21]
3	Malaysia	MahMeri	24	[23]
3	Malaysia	Seletar	23	[23]
3	Philippines	Aeta	25	[21]
4	Malaysia	Mendriq	13	[23]
4	Malaysia	Kintaq	15	[23]
4	Malaysia	Jehai	22	[23]
4	Malaysia	Bateq	10	[23]
5	Papua New Guinea	Bougainville	9	[2]
5	Papua New Guinea	Koinambe	11	[21]
5	Papua New Guinea	Kosipe	5	[21]
5	Papua New Guinea	5	17	[2]
5	Papua New Guinea	PNG Highlander	10	[21]
6	Cambodia	Cambodian	10	[2]
6	China	Dai	25	[2]
6	Vietnam	Kinh	25	[2]
6	Vietnam	Vietnamese	20	[24]
6	China	Lahu	8	[2]
6	China	Miao	10	[2]
6	China	South Han	25	[2]
6	China	She	10	[2]
6	China	Tujia	10	[2]
6	China	Han	25	[24]
6	China	Yi	10	[2]
6	China	Naxi	8	[2]
6	China	Han North China	10	[2]
6	Japan	Japanese	25	[2]
6	China	Mongolian	9	[2]
6	China	Tu	10	[2]
6	China	Xibo	9	[2]
6	China	Daur	9	[2]
6	China	Hezhen	8	[2]
6	India	Naga	4	[13]

7	Afghanistan	Afghan	24	25
7	Uzbekstan	Uzbek	15	25
7	China	Uygur	10	2
7	Pakistan	Hazara	22	25
7	Russia	Yakut	25	2
7	India	Indian Telugu UK	25	19
7	China	Oroqen	9	2

Supplementary table 2 –GLOBETROTTER[26] inferred dates in generations from present (G) for each studied population. The column 'Cluster' identidies the cluster of populations defined in fineSTRUCTURE and F_{ST} analyses. The best-fit models of admixture are labeled as: 1D: one date; 2D: multiple dates; MW: multiway one date; U: uncertain. The column 'best-fit 2' refers to the most probable scenario of admixture given by GLOBETROTTER[26] and the column 'best-fit 1' refers to the reclassification of models for which the bootstrap 95%CIs of both Null0 and Null1 models do not overlap (i.e. relabelled as U). P: p-value of evidence of any detectable admixture event obtained after 100 bootstrap resamplings of the NULL procedure. Date confidence intervals are based on 100 bootstrap replicates of the date inference. M corresponds to the additional R^2 explained by adding a second date versus assuming only a single date of admixture (M>0.35 to infer multiple dates event). FQ1 and FQ2 correspond respectively to the fit of a single admixture event and the fit of the first two principal components capturing the admixture events.

Cluster	Population	Null	Best-fit 1	Best-fit 2	p	R^2.1D	FQ1	FQ2	G.1D	G.1D. 95%CI	% 1D Source 1	1D Source 1	1D Source 2	% MW Source 1	MW Source 1	MW Source 2	M	G.2D.1	G.2D.1. 95%CI	% 2D.1 Source 1	2D.1 Source 1	2D.1 Source 2	G.2D.2	G.2D.2. 95%IC	% 2D.2 Source 1	2D.2 Source 1	2D.2 Source 2
1	Island Southeast Asia Indonesia Banjar	0	U	MW	0.01	0.91	0.92	0.99	17.90	11.25-24.18	0.31	Mainland Southeast Asia Cambodia Cambodian	Island Southeast Asia Indonesia Ma'anyan	0.39	Island Southeast Asia Indonesia Ma'anyan	Island Southeast Asia Phillippines Filipino	0.17	8.04		0.32	Mainland Southeast Asia Cambodia Cambodian	Island Southeast Asia Indonesia Ma'anyan	38.8		0.48	Island Southeast Asia Indonesia Ma'anyan	Island Southeast Asia Phillippines Filipino
1	Island Southeast Asia Indonesia Banjar	1	U	MW	0.01	0.88	0.95	0.99	37.76	30.44-46.55	0.40	Mainland Southeast Asia Cambodia Cambodian	Island Southeast Asia Indonesia Ma'anyan	0.42	Island Southeast Asia Indonesia Ma'anyan	Island Southeast Asia Phillippines Filipino	0.10	7.95		0.20	Island Southeast Asia Phillippines Filipino	Island Southeast Asia Indonesia Ma'anyan	52.37		0.28	Mainland Southeast Asia Cambodia Cambodian	Island Southeast Asia Indonesia Ma'anyan
1	Island Southeast Asia Indonesia SKDayak	0	MW	MW	0.01	0.54	0.88	1.00	17.15	1.75-33.02	0.36	Island Southeast Asia Phillippines Filipino	Island Southeast Asia Indonesia Ma'anyan	0.46	Island Southeast Asia Indonesia Ma'anyan	Island Southeast Asia Indonesia Ma'anyan	0.25	2.28		0.45	Island Southeast Asia Indonesia Ma'anyan	Island Southeast Asia Phillippines Filipino	46.37		0.48	Island Southeast Asia Phillippines Filipino	Island Southeast Asia Indonesia Ma'anyan
1	Island Southeast Asia Indonesia SKDayak	1	MW	MW	0.01	0.62	0.96	1.00	44.52	28.06-60.80	0.34	Island Southeast Asia Phillippines Filipino	Island Southeast Asia Indonesia Ma'anyan	0.48	Island Southeast Asia Indonesia Ma'anyan	Island Southeast Asia Phillippines Filipino	0.15	1.00		0.35	Island Southeast Asia Phillippines Filipino	Island Southeast Asia Indonesia Ma'anyan	56.32		0.33	Mainland Southeast Asia Cambodia Cambodian	Island Southeast Asia Indonesia Ma'anyan
1	Mainland Southeast Asia Malaysia Malay	0	2D	2D	0.01	0.97	0.94	0.99	13.66	13.59-22.36	0.31	South Asia India Khasi	Island Southeast Asia Phillippines Filipino	0.27	Island Southeast Asia Phillippines Filipino	Mainland Southeast Asia Cambodia Cambodian	0.49	2.57	1.37-11.90	0.27	Mainland Southeast Asia Cambodia Cambodian	Island Southeast Asia Phillippines Filipino	23.66	14.33-39.66	0.32	Mainland Southeast Asia Cambodia Cambodian	Island Southeast Asia Phillippines Filipino
1	Mainland Southeast Asia Malaysia Malay	1	2D	2D	0.01	0.96	0.96	0.99	19.01	10.67-14.13	0.28	South Asia India Khasi	Island Southeast Asia Phillippines Filipino	0.30	Island Southeast Asia Phillippines Filipino	Mainland Southeast Asia Cambodia Cambodian	0.59	6.17	2.33-17.50	0.17	Mainland Southeast Asia Cambodia Cambodian	Island Southeast Asia Phillippines Filipino	42.06	35.39-63.73	0.29	South Asia India Khasi	Island Southeast Asia Phillippines Filipino
2	Island Southeast Asia Indonesia Bajo	0	2D	2D	0.01	0.91	0.99	1.00	37.92	29.52-45.92	0.10	Melanesia Papua New Guinea PNG Highlander	Mainland Southeast Asia Malaysia Malay	0.27	Island Southeast Asia Indonesia Banjar	Mainland Southeast Asia Malaysia Malay	0.49	7.54	1.33-28.21	0.23	Mainland Southeast Asia Malaysia Malay	Mainland Southeast Asia Malaysia Malay	65.18	45.18-83.51	0.09	Melanesia Papua New Guinea PNG Highlander	Mainland Southeast Asia Malaysia Malay
2	Island Southeast Asia Indonesia Bajo	1	2D	2D	0.01	0.90	0.99	1.00	40.63	31.55-53.69	0.08	Melanesia Papua New Guinea PNG Highlander	Mainland Southeast Asia Malaysia Malay	0.26	Island Southeast Asia Indonesia Banjar	Mainland Southeast Asia Malaysia Malay	0.43	6.8	2.67-36.13	0.30	Mainland Southeast Asia Malaysia Malay	Mainland Southeast Asia Malaysia Malay	65.86	53.86-85.19	0.09	Melanesia Papua New Guinea PNG Highlander	Mainland Southeast Asia Malaysia Malay
2	Island Southeast Asia Indonesia Dusun	0	1D	1D	0.01	0.63	1.00	1.00	6.24	1.81-10.57	0.36	Island Southeast Asia Indonesia Banjar	Mainland Southeast Asia Malaysia Malay	0.49	Mainland Southeast Asia Malaysia Malay	Mainland Southeast Asia Malaysia Malay	0.04	1.00		0.43	Island Southeast Asia Indonesia Banjar	Mainland Southeast Asia Malaysia Malay	10.64		0.17	Island Southeast Asia Indonesia Banjar	Mainland Southeast Asia Malaysia Malay
2	Island Southeast Asia Indonesia Dusun	1	1D	1D	0.01	0.46	1.00	1.00	13.10	5.55-26.44	0.46	Mainland Southeast Asia Malaysia Malay	Island Southeast Asia Indonesia Banjar	0.37	Mainland Southeast Asia Malaysia Malay	Mainland Southeast Asia Malaysia Malay	0.09	5.77		0.30	Mainland Southeast Asia Malaysia Malay	Mainland Southeast Asia Malaysia Malay	63.54		0.35	Island Southeast Asia Indonesia Banjar	Mainland Southeast Asia Malaysia Malay
2	Island Southeast Asia Indonesia Mandar	0	MW	MW	0.01	0.92	0.84	1.00	54.54	40.63-69.82	0.13	Island Southeast Asia Indonesia Banjar	Mainland Southeast Asia Malaysia Malay	0.40	Mainland Southeast Asia Malaysia Malay	Island Southeast Asia Indonesia Banjar	0.20	9.03		0.14	Island Southeast Asia Indonesia Banjar	Mainland Southeast Asia Malaysia Malay	68.21		0.04	Melanesia Papua New Guinea 5	Mainland Southeast Asia Malaysia Malay
2	Island Southeast Asia Indonesia	1	MW	MW	0.01	0.89	0.84	1.00	58.38	42.03-69.04	0.14	Island Southeast Asia Indonesia	Mainland Southeast Asia Malaysia	0.39	Mainland Southeast Asia Malaysia Malay	Island Southeast Asia Indonesia Banjar	0.05	8.15		0.22	Mainland Southeast Asia Malaysia Malay	Mainland Southeast Asia Malaysia Malay	67.46		0.09	Melanesia Papua New Guinea 5	Mainland Southeast Asia Malaysia Malay

	Mandar											Banjar	Malay														
2	Island Southeast Asia Indonesia Murut	0	1D	1D	0.01	0.53	0.99	1.00	9.44	4.71-17.05	0.15	Island Southeast Asia Indonesia Banjar	Mainland Southeast Asia Malaysia Malay	0.07	Mainland Southeast Asia Malaysia Malay	Mainland Southeast Asia Malaysia Malay	0.14	5.61		0.44	Island Southeast Asia Indonesia Banjar	Mainland Southeast Asia Malaysia Malay	17.74		0.32	Mainland Southeast Asia Malaysia Malay	Mainland Southeast Asia Malaysia Malay
2	Island Southeast Asia Indonesia Murut	1	1D	1D	0.01	0.28	0.99	1.00	35.04	16.22-163.61	0.18	Island Southeast Asia Indonesia Banjar	Mainland Southeast Asia Malaysia Malay	0.22	Mainland Southeast Asia Malaysia Malay	Mainland Southeast Asia Malaysia Malay	0.03	2.73		0.40	Mainland Southeast Asia Malaysia Malay	Island Southeast Asia Indonesia Banjar	31.83		0.12	Island Southeast Asia Indonesia Banjar	Mainland Southeast Asia Malaysia Malay
2	Island Southeast Asia Indonesia Sumba	0	1D	1D	0.01	0.90	1.00	1.00	75.26	65.99-83.84	0.13	Melanesia Papua New Guinea PNG Highlander	Mainland Southeast Asia Malaysia Malay	0.16	Island Southeast Asia Indonesia Banjar	Mainland Southeast Asia Malaysia Malay	0.06	1.00		0.19	Island Southeast Asia Indonesia Banjar	Mainland Southeast Asia Malaysia Malay	71.75		0.15	Melanesia Papua New Guinea PNG Highlander	Mainland Southeast Asia Malaysia Malay
2	Island Southeast Asia Indonesia Sumba	1	1D	1D	0.01	0.85	1.00	1.00	82.28	65.23-94.55	0.13	Melanesia Papua New Guinea PNG Highlander	Mainland Southeast Asia Malaysia Malay	0.15	Island Southeast Asia Indonesia Banjar	Mainland Southeast Asia Malaysia Malay	0.10	3.03		0.12	Island Southeast Asia Indonesia Banjar	Mainland Southeast Asia Malaysia Malay	71.97		0.15	Melanesia Papua New Guinea PNG Highlander	Mainland Southeast Asia Malaysia Malay
2	Island Southeast Asia Philippines Casigurans	0	MW	MW	0.01	0.62	0.95	1.00	4.94	1.56-9.16	0.38	Mainland Southeast Asia Malaysia Malay	Mainland Southeast Asia Malaysia Malay	0.37	Mainland Southeast Asia Malaysia Malay	Mainland Southeast Asia Malaysia Malay	0.06	2.53		0.39	Island Southeast Asia Indonesia Banjar	Mainland Southeast Asia Malaysia Malay	12.58		0.17	Island Southeast Asia Philippines Tagbanua	Mainland Southeast Asia Malaysia Malay
2	Island Southeast Asia Philippines Casigurans	1	MW	MW	0.01	0.37	0.94	1.00	9.44	1.67-33.42	0.33	Mainland Southeast Asia Malaysia Malay	Mainland Southeast Asia Malaysia Malay	0.30	Island Southeast Asia Indonesia Banjar	Mainland Southeast Asia Malaysia Malay	0.05	1.00		0.46	Mainland Southeast Asia Malaysia Malay	Island Southeast Asia Indonesia Banjar	35.76		0.49	Mainland Southeast Asia Malaysia Malay	Mainland Southeast Asia Malaysia Malay
2	Island Southeast Asia Philippines Zambales	0	MW	MW	0.01	0.65	0.93	1.00	7.65	3.02-13.03	0.46	Mainland Southeast Asia Malaysia Malay	Island Southeast Asia Philippines Aeta	0.46	Mainland Southeast Asia Malaysia Malay	Mainland Southeast Asia Malaysia Malay	0.04	8.62		0.40	Island Southeast Asia Philippines Aeta	Mainland Southeast Asia Malaysia Malay	19.04		0.32	Island Southeast Asia Indonesia Banjar	Mainland Southeast Asia Malaysia Malay
2	Island Southeast Asia Philippines Zambales	1	MW	MW	0.01	0.35	0.96	1.00	13.75	6.24-20.03	0.48	Island Southeast Asia Philippines Aeta	Mainland Southeast Asia Malaysia Malay	0.41	Mainland Southeast Asia Malaysia Malay	Mainland Southeast Asia Malaysia Malay	0.02	2.42		0.42	Island Southeast Asia Philippines Aeta	Mainland Southeast Asia Malaysia Malay	11.73		0.41	Mainland Southeast Asia Malaysia Malay	Mainland Southeast Asia Malaysia Malay
2	Island Southeast Asia Phillippines Filipino	0	U	U	0.01	0.86	0.96	0.98	8.79	5.93-13.75	0.45	Mainland Southeast Asia Malaysia Malay	Island Southeast Asia Indonesia Banjar	0.33	Mainland Southeast Asia Malaysia Malay	Mainland Southeast Asia Malaysia Malay	0.11	1.00		0.39	Mainland Southeast Asia Malaysia Malay	Mainland Southeast Asia Malaysia Malay	15.26		0.5	Mainland Southeast Asia Malaysia Malay	Mainland Southeast Asia Malaysia Malay
2	Island Southeast Asia Phillippines Filipino	1	MW	MW	0.01	0.78	0.97	0.99	17.99	10.32-24.87	0.42	Mainland Southeast Asia Malaysia Malay	Island Southeast Asia Indonesia Banjar	0.23	Mainland Southeast Asia Malaysia Malay	Mainland Southeast Asia Malaysia Malay	0.09	8.33		0.38	Mainland Southeast Asia Malaysia Malay	Mainland Southeast Asia Malaysia Malay	70.01		0.4	Island Southeast Asia Indonesia Banjar	Mainland Southeast Asia Malaysia Malay
3	Island Southeast Asia Indonesia Lebbo	0	MW	MW	0.01	0.85	0.72	0.99	38.18	26.26-50.26	0.47	Mainland Southeast Asia Malaysia Malay	Island Southeast Asia Philippines Casigurans	0.10	Island Southeast Asia Philippines Zambales	Mainland Southeast Asia Malaysia Malay	0.20	4.33		0.40	Island Southeast Asia Philippines Zambales	Mainland Southeast Asia Malaysia Malay	48.42		0.47	Mainland Southeast Asia Malaysia Malay	Island Southeast Asia Philippines Casigurans
3	Island Southeast Asia Indonesia Lebbo	1	MW	MW	0.01	0.79	0.86	0.99	62.26	49.44-77.56	0.14	Melanesia Papua New Guinea PNG Highlander	Island Southeast Asia Phillippines Filipino	0.18	Island Southeast Asia Philippines Casigurans	Mainland Southeast Asia Malaysia Malay	0.04	1.00		0.47	Island Southeast Asia Philippines Zambales	Mainland Southeast Asia Malaysia Malay	64.51		0.14	Melanesia Papua New Guinea PNG Highlander	Island Southeast Asia Phillippines Filipino
3	Island Southeast Asia Philippines Aeta	0	2D	2D	0.01	0.94	1.00	1.00	14.13	10.52-17.80	0.13	South Asia India West Bengal Brahmin	Island Southeast Asia Philippines Zambales	0.30	Island Southeast Asia Philippines Zambales	Island Southeast Asia Philippines Zambales	0.41	1.22	1.00-13.89	0.31	Island Southeast Asia Phillippines Filipino	Island Southeast Asia Philippines Zambales	24.49	2.49-41.49	0.14	South Asia India Maratha	Island Southeast Asia Philippines Zambales
3	Island Southeast Asia Philippines Aeta	1	2D	2D	0.01	0.93	1.00	1.00	21.25	16.53-29.73	0.14	South Asia India Maratha	Island Southeast Asia Philippines Zambales	0.39	Island Southeast Asia Philippines Zambales	Island Southeast Asia Philippines Zambales	0.41	6.31	2.33-13.64	0.25	Island Southeast Asia Phillippines Filipino	Island Southeast Asia Philippines Zambales	46.58	26.91-68.58	0.17	South Asia India Maratha	Island Southeast Asia Philippines Zambales
3	Island Southeast Asia Philippines Agta	0	1D	1D	0.01	0.63	1.00	1.00	11.93	5.40-36.49	0.27	South Asia India Khasi	Island Southeast Asia Philippines Zambales	0.27	Island Southeast Asia Phillippines Filipino	Island Southeast Asia Phillippines Filipino	0.27	3.26		0.43	Island Southeast Asia Phillippines Filipino	Island Southeast Asia Phillippines Zambales	56.1		0.22	Melanesia Papua New Guinea 5	Island Southeast Asia Philippines Zambales
3	Island Southeast Asia Philippines Agta	1	1D	1D	0.01	0.25	1.00	1.00	58.65	33.02-101.00	0.29	South Asia India Bengali	Island Southeast Asia Philippines Zambales	0.41	Island Southeast Asia Phillippines Filipino	Island Southeast Asia Phillippines Filipino	0.03	1.00		0.49	Island Southeast Asia Phillippines Filipino	Island Southeast Asia Phillippines Casigurans	63.02		0.3	South Asia India Bengali	Island Southeast Asia Philippines Zambales
3	Island Southeast Asia Philippines Batak	0	U	1D	0.01	0.90	0.99	1.00	26.13	20.20-33.07	0.15	South Asia India Bengali	Island Southeast Asia Phillippines Filipino	0.28	Island Southeast Asia Philippines Zambales	Island Southeast Asia Phillippines Filipino	0.26	2.93		0.33	Island Southeast Asia Philippines Zambales	Island Southeast Asia Phillippines Filipino	36.96		0.14	Melanesia Papua New Guinea 5	Island Southeast Asia Phillippines Filipino
3	Island Southeast Asia Philippines	1	U	1D	0.01	0.87	1.00	1.00	59.63	48.47-70.01	0.17	South Asia India Maratha	Island Southeast Asia	0.20	Island Southeast Asia Philippines Zambales	Island Southeast Asia Phillippines Filipino	0.33	4.32		0.40	Island Southeast Asia Philippines Zambales	Island Southeast Asia Phillippines Filipino	73.2		0.18	South Asia India Maratha	Island Southeast Asia Philippines

	Batak												Phillippines Filipino														Zambales
3	Island Southeast Asia Philippines Tagbanua	0	U	1D	0.01	0.85	0.99	1.00	24.46	16.66-30.63	0.15	South Asia India Khasi	Island Southeast Asia Philippines Zambales	0.48	Island Southeast Asia Philippines Zambales	Mainland Southeast Asia Malaysia Malay	0.29	2.39		0.35	Island Southeast Asia Phillippines Filipino	Island Southeast Asia Philippines Zambales	34.49		0.27	Mainland Southeast Asia Malaysia Malay	Island Southeast Asia Philippines Zambales
3	Island Southeast Asia Philippines Tagbanua	1	U	1D	0.01	0.78	0.99	1.00	51.92	33.02-64.48	0.15	South Asia Bangladesh Bengali	Island Southeast Asia Philippines Zambales	0.19	Island Southeast Asia Philippines Zambales	Island Southeast Asia Phillippines Filipino	0.12	1.00		0.45	Island Southeast Asia Phillippines Filipino	Island Southeast Asia Phillippines Filipino	60.5		0.16	South Asia Bangladesh Bengali	Island Southeast Asia Philippines Zambales
3	Mainland Southeast Asia Malaysia CheWong	0	1D	1D	0.01	0.76	1.00	1.00	13.75	8.17-17.21	0.44	Mainland Southeast Asia Cambodia Cambodian	Mainland Southeast Asia Malaysia Mendriq	0.49	Mainland Southeast Asia Malaysia Mendriq	Mainland Southeast Asia Malaysia Mendriq	0.06	7.39		0.50	Mainland Southeast Asia Malaysia Mendriq	Mainland Southeast Asia Malaysia Mendriq	15.08		0.32	Mainland Southeast Asia Cambodia Cambodian	Mainland Southeast Asia Malaysia Mendriq
3	Mainland Southeast Asia Malaysia CheWong	1	1D	1D	0.01	0.65	1.00	1.00	24.57	17.58-31.21	0.39	Mainland Southeast Asia Cambodia Cambodian	Mainland Southeast Asia Malaysia Mendriq	0.43	Mainland Southeast Asia Malaysia Mendriq	Mainland Southeast Asia Malaysia Mendriq	0.03	11.24		0.21	Mainland Southeast Asia Malaysia Jehai	Mainland Southeast Asia Malaysia Mendriq	23.46		0.33	Mainland Southeast Asia Cambodia Cambodian	Mainland Southeast Asia Malaysia Mendriq
3	Mainland Southeast Asia Malaysia Jakun	0	1D	1D	0.01	0.76	1.00	1.00	12.83	8.16-15.81	0.33	Mainland Southeast Asia Malaysia Mendriq	Mainland Southeast Asia Malaysia Malay	0.16	Mainland Southeast Asia Cambodia Cambodian	Mainland Southeast Asia Malaysia Malay	0.08	9.9		0.31	Mainland Southeast Asia Malaysia Mendriq	Mainland Southeast Asia Malaysia Malay	19.8		0.41	Island Southeast Asia Indonesia Banjar	Mainland Southeast Asia Cambodia Cambodian
3	Mainland Southeast Asia Malaysia Jakun	1	1D	1D	0.01	0.60	1.00	1.00	17.63	10.75-25.17	0.38	Mainland Southeast Asia Malaysia Mendriq	Mainland Southeast Asia Malaysia Malay	0.16	Mainland Southeast Asia Cambodia Cambodian	Mainland Southeast Asia Malaysia Malay	0.04	1.00		0.27	Mainland Southeast Asia Malaysia Malay	Mainland Southeast Asia Malaysia Malay	20.39		0.37	Mainland Southeast Asia Malaysia Mendriq	Mainland Southeast Asia Malaysia Malay
3	Mainland Southeast Asia Malaysia MahMeri	0	1D	1D	0.01	0.58	1.00	1.00	15.30	10.48-19.40	0.30	Mainland Southeast Asia Malaysia Mendriq	Mainland Southeast Asia Malaysia Malay	0.24	Mainland Southeast Asia Cambodia Cambodian	Mainland Southeast Asia Malaysia Malay	0.04	4.76		0.38	Mainland Southeast Asia Malaysia Malay	Mainland Southeast Asia Cambodia Cambodian	14.85		0.45	Mainland Southeast Asia Malaysia Mendriq	Mainland Southeast Asia Malaysia Malay
3	Mainland Southeast Asia Malaysia MahMeri	1	1D	1D	0.01	0.46	1.00	1.00	17.90	7.86-24.18	0.29	Mainland Southeast Asia Malaysia Mendriq	Mainland Southeast Asia Malaysia Malay	0.25	Mainland Southeast Asia Cambodia Cambodian	Mainland Southeast Asia Malaysia Malay	0.01	1.00		0.13	Island Southeast Asia Phillippines Filipino	Mainland Southeast Asia Cambodia Cambodian	17.83		0.33	Mainland Southeast Asia Malaysia Malay	Mainland Southeast Asia Malaysia Malay
3	Mainland Southeast Asia Malaysia Seletar	0	1D	1D	0.01	0.61	1.00	1.00	12.76	6.45-24.18	0.02	Mainland Southeast Asia Malaysia Jehai	Mainland Southeast Asia Malaysia Malay	0.16	East Asia China Han NChina	Mainland Southeast Asia Malaysia Malay	0.12	1.00		0.02	Island Southeast Asia Phillippines Igorot	Mainland Southeast Asia Malaysia Malay	26.8		0.05	Mainland Southeast Asia Malaysia Mendriq	Mainland Southeast Asia Malaysia Malay
3	Mainland Southeast Asia Malaysia Seletar	1	1D	1D	0.01	0.58	1.00	1.00	12.26	2.72-29.35	0.01	Mainland Southeast Asia Malaysia Mendriq	Mainland Southeast Asia Malaysia Malay	0.11	East Asia China Tujia	Mainland Southeast Asia Malaysia Malay	0.07	5.59		0.02	Island Southeast Asia Phillippines Igorot	Mainland Southeast Asia Malaysia Malay	37.08		0.04	Mainland Southeast Asia Malaysia Mendriq	Mainland Southeast Asia Malaysia Malay
3	Mainland Southeast Asia Malaysia Temuan	0	1D	1D	0.01	0.48	0.99	1.00	18.34	4.94-24.87	0.43	Mainland Southeast Asia Malaysia Malay	Mainland Southeast Asia Cambodia Cambodian	0.31	Mainland Southeast Asia Cambodia Cambodian	Mainland Southeast Asia Malaysia Malay	0.06	1.00		0.48	Mainland Southeast Asia Cambodia Cambodian	Mainland Southeast Asia Malaysia Malay	23.14		0.35	Mainland Southeast Asia Malaysia Mendriq	Mainland Southeast Asia Malaysia Malay
3	Mainland Southeast Asia Malaysia Temuan	1	1D	1D	0.01	0.41	0.99	1.00	19.46	8.28-26.79	0.45	Mainland Southeast Asia Malaysia Malay	Mainland Southeast Asia Cambodia Cambodian	0.28	Mainland Southeast Asia Cambodia Cambodian	Mainland Southeast Asia Malaysia Malay	0.03	1.00		0.36	Mainland Southeast Asia Malaysia Malay	Mainland Southeast Asia Malaysia Malay	23.86		0.42	Mainland Southeast Asia Malaysia Mendriq	Mainland Southeast Asia Malaysia Malay
5	Melanesia Papua New Guinea Bougainville	0	1D	1D	0.01	0.65	0.99	1.00	24.74	15.01-34.22	0.40	South Asia India Khasi	Island Southeast Asia Phillippines Filipino	0.31	Island Southeast Asia Phillippines Filipino	Island Southeast Asia Indonesia Sumba	0.12	2.56		0.41	Island Southeast Asia Phillippines Filipino	Island Southeast Asia Indonesia Sumba	25.95		0.46	Island Southeast Asia Indonesia Sumba	South Asia India Khasi
5	Melanesia Papua New Guinea Bougainville	1	1D	1D	0.01	0.37	0.99	0.99	51.42	22.03-82.00	0.22	Island Southeast Asia Phillippines Filipino	Island Southeast Asia Indonesia Sumba	0.45	Island Southeast Asia Indonesia Sumba	Island Southeast Asia Phillippines Filipino	0.04	1.00		0.32	Island Southeast Asia Phillippines Filipino	Island Southeast Asia Indonesia Sumba	43.8		0.23	Island Southeast Asia Phillippines Filipino	Island Southeast Asia Indonesia Sumba
5	Melanesia Papua New Guinea Koinambe	0	MW	MW	0.01	0.55	0.96	1.00	12.22	10.45-21.79	0.32	Island Southeast Asia Indonesia Sumba	Island Southeast Asia Indonesia Sumba	0.38	Island Southeast Asia Indonesia Sumba	Island Southeast Asia Indonesia Sumba	0.07	7.97		0.40	Island Southeast Asia Indonesia Sumba	Island Southeast Asia Indonesia Sumba	46.93		0.33	Island Southeast Asia Indonesia Sumba	Island Southeast Asia Indonesia Sumba
5	Melanesia Papua New Guinea Koinambe	1	U	U	0.01	0.04	0.24	0.25	7.50	3.26-14.61	0.48	Island Southeast Asia Indonesia Sumba	Island Southeast Asia Indonesia Sumba	0.45	Island Southeast Asia Indonesia Sumba	Island Southeast Asia Indonesia Sumba	0.06	2.23		0.49	Island Southeast Asia Indonesia Sumba	Island Southeast Asia Indonesia Sumba	10.63		0.39	Island Southeast Asia Indonesia Sumba	Island Southeast Asia Indonesia Sumba
5	Melanesia Papua New Guinea Kosipe	0	1D	1D	0.01	0.30	1.00	1.00	9.10	2.96-26.97	0.32	Island Southeast Asia Indonesia Sumba	Island Southeast Asia Indonesia Sumba	0.21	Island Southeast Asia Indonesia Sumba	Island Southeast Asia Indonesia Sumba	0.08	2.77		0.42	Island Southeast Asia Indonesia Sumba	Island Southeast Asia Indonesia Sumba	34.9		0.38	Island Southeast Asia Indonesia Sumba	Island Southeast Asia Indonesia Sumba
5	Melanesia Papua New Guinea Kosipe	1	U	U	0.01	0.04	0.61	0.65	1.64	1.13-10.03	0.40	Island Southeast Asia Indonesia Sumba	Island Southeast Asia Indonesia Sumba	0.33	Island Southeast Asia Indonesia Sumba	Island Southeast Asia Indonesia Sumba	0.04	1.92		0.34	Island Southeast Asia Indonesia Bajo	Island Southeast Asia Indonesia Sumba	8.15		0.36	Island Southeast Asia Indonesia Bajo	Island Southeast Asia Indonesia Sumba

5	Melanesia Papua New Guinea 5	0	MW	MW	0.01	0.53	0.95	1.00	12.03	7.65-30.77	0.28	Island Southeast Asia Indonesia Sumba	Island Southeast Asia Indonesia Sumba	0.29	Island Southeast Asia Indonesia Sumba	Island Southeast Asia Indonesia Sumba	0.17	1.26		0.36	Island Southeast Asia Indonesia Sumba	Island Southeast Asia Indonesia Sumba	37.96		0.29	Island Southeast Asia Indonesia Sumba	Island Southeast Asia Indonesia Sumba
5	Melanesia Papua New Guinea 5	1	U	U	0.01	0.11	0.86	0.87	1.00	1.00-126.16	0.43	Island Southeast Asia Indonesia Sumba	Island Southeast Asia Indonesia Sumba	0.45	Island Southeast Asia Indonesia Sumba	Island Southeast Asia Indonesia Sumba	0.05	2.4		0.44	Island Southeast Asia Indonesia Sumba	Island Southeast Asia Indonesia Sumba	14.05		0.46	Island Southeast Asia Indonesia Sumba	Island Southeast Asia Indonesia Sumba
5	Melanesia Papua New Guinea PNG Highlander	0	MW	MW	0.01	0.47	0.96	1.00	12.36	1.56-11.51	0.37	Island Southeast Asia Indonesia Sumba	Island Southeast Asia Indonesia Sumba	0.48	Island Southeast Asia Indonesia Sumba	Island Southeast Asia Indonesia Sumba	0.08	8.3		0.45	Island Southeast Asia Indonesia Sumba	Island Southeast Asia Indonesia Sumba	42.25		0.41	Island Southeast Asia Indonesia Sumba	Island Southeast Asia Indonesia Sumba
5	Melanesia Papua New Guinea PNG Highlander	1	U	U	0.01	0.04	0.87	0.88	55.84	8.17-20.37	0.41	Island Southeast Asia Indonesia Sumba	Island Southeast Asia Indonesia Sumba	0.48	Island Southeast Asia Indonesia Sumba	Island Southeast Asia Indonesia Sumba	0.04	1.00		0.29	Island Southeast Asia Indonesia Mandar	Island Southeast Asia Indonesia Sumba	40.77		0.45	Island Southeast Asia Indonesia Sumba	Island Southeast Asia Indonesia Sumba
4	Mainland Southeast Asia Malaysia Bateq	0	U	MW	0.01	0.64	0.97	1.00	13.47	8.93-23.63	0.42	Mainland Southeast Asia Malaysia Temuan	Mainland Southeast Asia Malaysia CheWong	0.47	Mainland Southeast Asia Malaysia CheWong	Mainland Southeast Asia Malaysia CheWong	0.26	10.96		0.35	Mainland Southeast Asia Malaysia Temuan	Mainland Southeast Asia Malaysia CheWong	67.96		0.4	Mainland Southeast Asia Malaysia CheWong	Mainland Southeast Asia Malaysia CheWong
4	Mainland Southeast Asia Malaysia Bateq	1	U	1D	0.01	0.59	1.00	1.00	34.73	25.38-47.18	0.36	Mainland Southeast Asia Malaysia Malay	Mainland Southeast Asia Malaysia CheWong	0.32	Mainland Southeast Asia Malaysia CheWong	Mainland Southeast Asia Malaysia CheWong	0.04	1.00		0.32	Mainland Southeast Asia Malaysia CheWong	Mainland Southeast Asia Malaysia CheWong	40.37		0.38	Mainland Southeast Asia Malaysia Malay	Mainland Southeast Asia Malaysia CheWong
4	Mainland Southeast Asia Malaysia Jehai	0	U	1D	0.01	0.63	0.99	1.00	17.00	9.88-21.91	0.34	Mainland Southeast Asia Cambodia Cambodian	Mainland Southeast Asia Malaysia CheWong	0.22	Mainland Southeast Asia Malaysia Jakun	Mainland Southeast Asia Malaysia CheWong	0.16	11.08		0.41	Mainland Southeast Asia Malaysia CheWong	Mainland Southeast Asia Malaysia CheWong	42.06		0.49	Mainland Southeast Asia Malaysia CheWong	Mainland Southeast Asia Cambodia Cambodian
4	Mainland Southeast Asia Malaysia Jehai	1	U	1D	0.01	0.58	1.00	1.00	57.59	47.82-70.99	0.47	Mainland Southeast Asia Cambodia Cambodian	Mainland Southeast Asia Malaysia CheWong	0.31	Mainland Southeast Asia Malaysia CheWong	Mainland Southeast Asia Malaysia CheWong	0.25	9.06		0.18	Mainland Southeast Asia Malaysia CheWong	Mainland Southeast Asia Malaysia CheWong	46.06		0.33	Mainland Southeast Asia Cambodia Cambodian	Mainland Southeast Asia Malaysia CheWong
4	Mainland Southeast Asia Malaysia Kintaq	0	U	2D	0.01	0.69	0.97	0.99	11.76	6.28-17.63	0.29	Mainland Southeast Asia Cambodia Cambodian	Mainland Southeast Asia Malaysia CheWong	0.31	Mainland Southeast Asia Malaysia CheWong	Mainland Southeast Asia Malaysia CheWong	0.37	17.00	2.67-30.33	0.37	Mainland Southeast Asia Cambodia Cambodian	Mainland Southeast Asia Malaysia CheWong	172.00	163.00-183.33	0.37	Mainland Southeast Asia Malaysia CheWong	Mainland Southeast Asia Malaysia CheWong
4	Mainland Southeast Asia Malaysia Kintaq	1	U	1D	0.01	0.65	1.00	1.00	38.61	25.30-51.25	0.33	Mainland Southeast Asia Malaysia Malay	Mainland Southeast Asia Malaysia CheWong	0.27	Mainland Southeast Asia Malaysia CheWong	Mainland Southeast Asia Malaysia CheWong	0.08	7.01		0.35	Mainland Southeast Asia Malaysia CheWong	Mainland Southeast Asia Malaysia CheWong	34.89		0.3	Mainland Southeast Asia Malaysia Malay	Mainland Southeast Asia Malaysia CheWong
4	Mainland Southeast Asia Malaysia Mendriq	0	U	U	0.01	0.79	0.96	0.98	14.98	7.78-20.61	0.26	Mainland Southeast Asia Malaysia Temuan	Mainland Southeast Asia Malaysia CheWong	0.43	Mainland Southeast Asia Malaysia CheWong	Mainland Southeast Asia Malaysia CheWong	0.30	18.03		0.30	Mainland Southeast Asia Malaysia Temuan	Mainland Southeast Asia Malaysia CheWong	124.05		0.47	Mainland Southeast Asia Malaysia CheWong	Mainland Southeast Asia Malaysia CheWong
4	Mainland Southeast Asia Malaysia Mendriq	1	1D	1D	0.01	0.68	0.99	0.99	35.04	18.78-45.61	0.35	Mainland Southeast Asia Cambodia Cambodian	Mainland Southeast Asia Malaysia CheWong	0.48	Mainland Southeast Asia Malaysia CheWong	Mainland Southeast Asia Malaysia CheWong	0.06	1.00		0.40	Mainland Southeast Asia Malaysia CheWong	Mainland Southeast Asia Malaysia Temuan	42.53		0.38	Mainland Southeast Asia Cambodia Cambodian	Mainland Southeast Asia Malaysia CheWong
6	East Asia China Dai	0	MW	MW	0.01	0.66	0.97	0.99	7.57	3.02-11.19	0.48	Mainland Southeast Asia Malaysia Malay	Mainland Southeast Asia Malaysia CheWong	0.45	Mainland Southeast Asia Malaysia Malay	Mainland Southeast Asia Malaysia Malay	0.14	1.15		0.13	Island Southeast Asia Indonesia Banjar	Mainland Southeast Asia Malaysia Malay	22.12		0.21	Mainland Southeast Asia Malaysia Malay	Mainland Southeast Asia Malaysia Malay
6	East Asia China Dai	1	1D	1D	0.01	0.22	0.99	1.00	34.22	12.56-75.37	0.45	South Asia India Khasi	Mainland Southeast Asia Malaysia Malay	0.35	Mainland Southeast Asia Malaysia Malay	Mainland Southeast Asia Malaysia Malay	0.04	8.35		0.35	Mainland Southeast Asia Malaysia Malay	Mainland Southeast Asia Malaysia Malay	65.00		0.47	South Asia India Khasi	Mainland Southeast Asia Malaysia Malay
6	East Asia China Daur	0	1D	1D	0.01	0.81	1.00	1.00	17.13	12.89-22.03	0.37	South Asia India Khasi	East Asia China Oroqen	0.31	East Asia China Oroqen	East Asia China Oroqen	0.07	1.00		0.24	Mainland Southeast Asia Myanmar Burmese	East Asia China Oroqen	19.37		0.38	South Asia India Khasi	East Asia China Oroqen
6	East Asia China Daur	1	1D	1D	0.01	0.71	1.00	1.00	19.68	12.49-27.33	0.37	South Asia India Khasi	East Asia China Oroqen	0.26	East Asia China Oroqen	East Asia China Oroqen	0.05	1.00		0.14	South Asia India Khasi	East Asia China Oroqen	24.26		0.37	South Asia India Khasi	East Asia China Oroqen
6	East Asia China Han	0	MW	MW	0.01	0.69	0.97	0.99	6.24	3.85-9.04	0.40	Mainland Southeast Asia Myanmar Burmese	Mainland Southeast Asia Malaysia Malay	0.37	South Asia India Khasi	South Asia India Khasi	0.20	2.03		0.42	South Asia India Khasi	South Asia India Khasi	33.07		0.42	Mainland Southeast Asia Myanmar Burmese	Mainland Southeast Asia Malaysia Malay
6	East Asia China Han	1	1D	1D	0.01	0.24	0.99	0.99	26.61	1.04-49.12	0.24	Mainland Southeast Asia Malaysia Malay	South Asia India Khasi	0.48	South Asia India Khasi	South Asia India Khasi	0.07	1.00		0.40	Mainland Southeast Asia Myanmar Burmese	Mainland Southeast Asia Malaysia Malay	51.88		0.46	Mainland Southeast Asia Myanmar Burmese	Mainland Southeast Asia Malaysia Malay
6	East Asia China Han NChina	0	MW	MW	0.01	0.60	0.96	0.99	17.37	5.55-29.35	0.45	Mainland Southeast Asia Myanmar Burmese	South Asia India Khasi	0.27	South Asia India Khasi	Mainland Southeast Asia Myanmar Burmese	0.19	2.04		0.44	Mainland Southeast Asia Myanmar Burmese	South Asia India Khasi	36.54		0.46	Mainland Southeast Asia Malaysia Malay	Mainland Southeast Asia Myanmar Burmese
6	East Asia China Han NChina	1	U	U	0.01	0.34	0.97	0.98	34.22	6.08-45.92	0.45	Mainland Southeast Asia Myanmar Burmese	Mainland Southeast Asia Malaysia Malay	0.30	South Asia India Khasi	Mainland Southeast Asia Myanmar Burmese	0.06	1.00		0.45	Mainland Southeast Asia Myanmar Burmese	Mainland Southeast Asia Myanmar Burmese	40.27		0.45	Mainland Southeast Asia Malaysia Malay	Mainland Southeast Asia Myanmar Burmese

6	East Asia China Hezhen	0	1D	1D	0.01	0.83	1.00	1.00	7.02	4.04-9.61	0.48	South Asia India Khasi	East Asia China Oroqen	0.16	East Asia China Oroqen	East Asia China Oroqen	0.24	1.48		0.47	South Asia India Khasi	East Asia China Oroqen	24.81		0.42	Mainland Southeast Asia Myanmar Burmese	East Asia China Oroqen
6	East Asia China Hezhen	1	1D	1D	0.01	0.74	1.00	1.00	8.03	4.73-12.42	0.47	South Asia India Khasi	East Asia China Oroqen	0.24	East Asia China Oroqen	East Asia China Oroqen	0.12	2.00		0.42	South Asia India Khasi	East Asia China Oroqen	28.21		0.49	East Asia China Oroqen	Mainland Southeast Asia Myanmar Burmese
6	East Asia China Miao	0	1D	1D	0.01	0.44	1.00	1.00	11.76	3.15-44.05	0.44	South Asia India Khasi	Mainland Southeast Asia Malaysia Malay	0.44	Mainland Southeast Asia Malaysia Malay	South Asia India Khasi	0.18	2.09		0.42	South Asia India Khasi	Mainland Southeast Asia Malaysia Malay	47.23		0.48	Mainland Southeast Asia Myanmar Burmese	Mainland Southeast Asia Malaysia Malay
6	East Asia China Miao	1	1D	1D	0.01	0.23	0.99	1.00	36.28	6.35-55.15	0.48	Mainland Southeast Asia Myanmar Burmese	Mainland Southeast Asia Malaysia Malay	0.34	South Asia India Khasi	South Asia India Khasi	0.06	17.16		0.49	Mainland Southeast Asia Malaysia Malay	South Asia India Khasi	41.29		0.44	Mainland Southeast Asia Malaysia Malay	Mainland Southeast Asia Myanmar Burmese
6	East Asia China Mongolian	0	MW	MW	0.01	0.82	0.96	1.00	15.50	11.00-19.23	0.47	South Asia India Khasi	East Asia China Oroqen	0.11	Central Asia Afghanistan	Mainland Southeast Asia Myanmar Burmese	0.17	1.2		0.41	Mainland Southeast Asia Myanmar Burmese	East Asia China Oroqen	18.00		0.45	East Asia China Oroqen	South Asia India Khasi
6	East Asia China Mongolian	1	MW	MW	0.01	0.66	0.95	1.00	17.80	13.38-23.85	0.43	East Asia China Oroqen	South Asia India Khasi	0.15	Central Asia Uzbekstan Uzbek	Mainland Southeast Asia Myanmar Burmese	0.05	2.12		0.50	Mainland Southeast Asia Myanmar Burmese	Mainland Southeast Asia Myanmar Burmese	18.07		0.35	East Asia China Oroqen	Mainland Southeast Asia Myanmar Burmese
6	East Asia China Naxi	0	MW	MW	0.01	0.41	0.94	0.99	12.16	3.15-19.01	0.46	South Asia India Naga	South Asia India Khasi	0.30	Mainland Southeast Asia Myanmar Burmese	South Asia India Khasi	0.12	1.08		0.47	South Asia India Khasi	Mainland Southeast Asia Myanmar Burmese	16.67		0.48	Mainland Southeast Asia Malaysia Malay	South Asia India Tripuri
6	East Asia China Naxi	1	U	U	0.01	0.19	0.88	0.98	16.22	2.34-31.84	0.34	Mainland Southeast Asia Malaysia Malay	South Asia India Khasi	0.44	Mainland Southeast Asia Myanmar Burmese	South Asia India Khasi	0.04	1.00		0.47	South Asia India Tripuri	Mainland Southeast Asia Myanmar Burmese	20.43		0.49	South Asia India Tripuri	South Asia India Khasi
6	East Asia China S Han	0	1D	1D	0.01	0.66	0.98	1.00	6.52	2.93-10.26	0.50	Mainland Southeast Asia Malaysia Malay	South Asia India Khasi	0.46	South Asia India Khasi	South Asia India Khasi	0.29	2.62		0.39	Mainland Southeast Asia Malaysia Malay	South Asia India Khasi	61.52		0.48	Mainland Southeast Asia Malaysia Malay	Mainland Southeast Asia Myanmar Burmese
6	East Asia China S Han	1	1D	1D	0.01	0.33	1.00	1.00	36.70	2.59-69.04	0.38	Mainland Southeast Asia Malaysia Malay	South Asia India Khasi	0.45	South Asia India Khasi	South Asia India Khasi	0.05	1.00		0.42	Mainland Southeast Asia Malaysia Malay	South Asia India Khasi	52.97		0.37	Mainland Southeast Asia Malaysia Malay	South Asia India Khasi
6	East Asia China She	0	U	U	0.01	0.59	0.95	0.98	3.15	1.24-8.23	0.46	Mainland Southeast Asia Malaysia Malay	South Asia India Khasi	0.43	South Asia India Khasi	Mainland Southeast Asia Malaysia Malay	0.13	2.03		0.42	South Asia India Khasi	Mainland Southeast Asia Malaysia Malay	86.4		0.46	Mainland Southeast Asia Malaysia Malay	South Asia India Khasi
6	East Asia China She	1	U	U	0.01	0.05	0.96	0.98	98.52	3.78-145.04	0.46	Mainland Southeast Asia Myanmar Burmese	Mainland Southeast Asia Malaysia Malay	0.47	Island Southeast Asia Phillippines Filipino	Mainland Southeast Asia Malaysia Malay	0.04	1.1		0.40	South Asia India Khasi	Mainland Southeast Asia Malaysia Malay	90.86		0.49	Mainland Southeast Asia Myanmar Burmese	Mainland Southeast Asia Malaysia Malay
6	East Asia China Tu	0	U	U	0.01	0.90	0.91	0.98	26.79	19.46-33.42	0.21	Central Asia Uzbekstan Uzbek	South Asia India Khasi	0.40	South Asia India Khasi	Mainland Southeast Asia Myanmar Burmese	0.11	1.26		0.34	Mainland Southeast Asia Myanmar Burmese	Mainland Southeast Asia Myanmar Burmese	31.48		0.22	Central Asia Uzbekstan Uzbek	South Asia India Khasi
6	East Asia China Tu	1	U	U	0.01	0.77	0.93	0.98	30.68	23.26-39.92	0.23	Central Asia Uzbekstan Uzbek	South Asia India Khasi	0.44	South Asia India Khasi	Mainland Southeast Asia Myanmar Burmese	0.06	1.00		0.36	Mainland Southeast Asia Myanmar Burmese	Mainland Southeast Asia Myanmar Burmese	32.4		0.22	Central Asia Uzbekstan Uzbek	South Asia India Khasi
6	East Asia China Tujia	0	1D	1D	0.01	0.49	0.99	0.99	6.84	1.44-18.78	0.46	South Asia India Khasi	Mainland Southeast Asia Malaysia Malay	0.37	South Asia India Khasi	South Asia India Khasi	0.09	1.00		0.49	South Asia India Garo	Mainland Southeast Asia Malaysia Malay	25.43		0.42	South Asia India Khasi	Mainland Southeast Asia Malaysia Malay
6	East Asia China Tujia	1	1D	1D	0.01	0.15	0.98	0.99	13.85	2.61-54.46	0.42	Mainland Southeast Asia Malaysia Malay	South Asia India Khasi	0.49	South Asia India Khasi	South Asia India Khasi	0.04	1.00		0.46	Mainland Southeast Asia Malaysia Malay	South Asia India Khasi	43.84		0.44	Mainland Southeast Asia Myanmar Burmese	Mainland Southeast Asia Malaysia Malay
6	East Asia China Xibo	0	MW	MW	0.01	0.92	0.93	1.00	11.73	8.44-17.31	0.47	South Asia India Khasi	East Asia China Oroqen	0.13	Central Asia Uzbekstan Uzbek	Mainland Southeast Asia Myanmar Burmese	0.30	5.23		0.33	South Asia India Khasi	Mainland Southeast Asia Myanmar Burmese	30.73		0.45	South Asia India Khasi	East Asia China Oroqen
6	East Asia China Xibo	1	MW	MW	0.01	0.81	0.93	1.00	13.47	7.91-17.96	0.46	South Asia India Khasi	East Asia China Oroqen	0.14	Central Asia Uzbekstan Uzbek	Mainland Southeast Asia Myanmar Burmese	0.12	6.28		0.34	South Asia India Khasi	Mainland Southeast Asia Myanmar Burmese	32.39		0.48	South Asia India Khasi	East Asia China Oroqen
6	East Asia China Yi	0	U	U	0.01	0.54	0.91	0.98	16.22	7.60-27.69	0.28	Mainland Southeast Asia Malaysia Malay	Mainland Southeast Asia Myanmar Burmese	0.38	South Asia India Khasi	South Asia India Khasi	0.20	1.00		0.47	South Asia India Khasi	Mainland Southeast Asia Myanmar Burmese	22.21		0.32	Mainland Southeast Asia Malaysia Malay	Mainland Southeast Asia Myanmar Burmese
6	East Asia China Yi	1	U	U	0.01	0.34	0.86	0.98	23.01	16.94-34.22	0.24	Mainland Southeast Asia Malaysia Malay	Mainland Southeast Asia Myanmar Burmese	0.25	South Asia India Khasi	South Asia India Khasi	0.03	1.19		0.48	Mainland Southeast Asia Myanmar Burmese	South Asia India Khasi	17.24		0.24	Mainland Southeast Asia Malaysia Malay	Mainland Southeast Asia Myanmar Burmese
6	East Asia Japan Japanese	0	U	U	0.01	0.65	0.74	0.97	4.83	1.57-9.91	0.49	Mainland Southeast Asia Myanmar Burmese	South Asia India Khasi	0.43	Mainland Southeast Asia Myanmar Burmese	Mainland Southeast Asia Myanmar Burmese	0.13	1.00		0.42	South Asia India Khasi	Mainland Southeast Asia Myanmar Burmese	54.11		0.49	Mainland Southeast Asia Myanmar Burmese	Mainland Southeast Asia Malaysia Malay

6	East Asia Japan Japanese	1	U	U	0.01	0.11	0.93	0.98	60.08	3.57-93.64	0.46	Mainland Southeast Asia Myanmar Burmese	South Asia India Khasi	0.49	Mainland Southeast Asia Myanmar Burmese	South Asia India Khasi	0.06	1.03		0.48	Mainland Southeast Asia Myanmar Burmese	South Asia India Khasi	79.17		0.47	Mainland Southeast Asia Malaysia Malay	Mainland Southeast Asia Myanmar Burmese
6	Mainland Southeast Asia Cambodia Cambodian	0	1D	1D	0.01	0.88	0.99	1.00	22.06	14.81-28.10	0.15	East Asia China Uygur	Mainland Southeast Asia Malaysia Malay	0.43	Mainland Southeast Asia Malaysia Malay	Mainland Southeast Asia Malaysia Malay	0.11	4.22		0.40	Mainland Southeast Asia Malaysia Malay	Mainland Southeast Asia Malaysia Malay	29.86		0.19	East Asia China Uygur	Mainland Southeast Asia Malaysia Malay
6	Mainland Southeast Asia Cambodia Cambodian	1	1D	1D	0.01	0.79	0.99	1.00	29.17	19.68-39.26	0.17	South Asia India Bengali	Mainland Southeast Asia Malaysia Malay	0.40	Mainland Southeast Asia Malaysia Malay	Mainland Southeast Asia Malaysia Malay	0.05	1.00		0.42	Mainland Southeast Asia Malaysia Malay	Mainland Southeast Asia Malaysia Malay	31.79		0.17	South Asia India Bengali	Mainland Southeast Asia Malaysia Malay
6	Mainland Southeast Asia Vietnam Kinh	0	1D	1D	0.01	0.74	1.00	1.00	13.38	8.12-24.52	0.20	Mainland Southeast Asia Myanmar Burmese	Mainland Southeast Asia Malaysia Malay	0.48	Mainland Southeast Asia Malaysia Malay	Mainland Southeast Asia Malaysia Malay	0.27	4.26		0.22	Mainland Southeast Asia Malaysia Malay	Mainland Southeast Asia Malaysia Malay	48.02		0.47	South Asia India Khasi	Mainland Southeast Asia Malaysia Malay
6	Mainland Southeast Asia Vietnam Kinh	1	1D	1D	0.01	0.54	0.99	1.00	45.61	25.21-63.00	0.33	Mainland Southeast Asia Myanmar Burmese	Mainland Southeast Asia Malaysia Malay	0.31	Mainland Southeast Asia Malaysia Malay	Mainland Southeast Asia Malaysia Malay	0.05	1.00		0.39	Mainland Southeast Asia Malaysia Malay	Mainland Southeast Asia Malaysia Malay	51.59		0.35	Mainland Southeast Asia Myanmar Burmese	Mainland Southeast Asia Malaysia Malay
6	Mainland Southeast Asia Vietnam Vietnamese	0	1D	1D	0.01	0.59	0.99	1.00	23.51	5.75-34.83	0.46	South Asia India Khasi	Mainland Southeast Asia Malaysia Malay	0.42	Mainland Southeast Asia Malaysia Malay	Mainland Southeast Asia Malaysia Malay	0.23	1.29		0.49	Mainland Southeast Asia Malaysia Malay	Mainland Southeast Asia Malaysia Malay	42.36		0.44	South Asia India Khasi	Mainland Southeast Asia Malaysia Malay
6	Mainland Southeast Asia Vietnam Vietnamese	1	1D	1D	0.01	0.36	1.00	1.00	42.53	25.56-59.01	0.37	South Asia India Khasi	Mainland Southeast Asia Malaysia Malay	0.49	Mainland Southeast Asia Malaysia Malay	Mainland Southeast Asia Malaysia Malay	0.06	1.00		0.48	Mainland Southeast Asia Malaysia Malay	Mainland Southeast Asia Malaysia Malay	48.78		0.33	South Asia India Khasi	Mainland Southeast Asia Malaysia Malay
8	Mainland Southeast Asia Myanmar Burmese	0	MW	MW	0.01	0.98	0.95	1.00	20.37	17.39-24.97	0.18	South Asia India Brahmin	East Asia China Mongolian	0.36	East Asia China Tujia	East Asia China Mongolian	0.28	13.65		0.20	South Asia India Bengali	East Asia China Mongolian	53.49		0.17	East Asia China Uygur	East Asia China Mongolian
8	Mainland Southeast Asia Myanmar Burmese	1	MW	MW	0.01	0.97	0.94	1.00	21.77	19.15-25.36	0.19	South Asia India Brahmin	East Asia China Mongolian	0.37	East Asia China Tujia	East Asia China Mongolian	0.19	14.27		0.18	South Asia India West Bengal Brahmin	East Asia China Mongolian	60.87		0.19	South Asia India Brahmin	East Asia China Mongolian
8	South Asia India Garo	0	MW	MW	0.01	0.68	0.97	1.00	31.64	13.29-58.65	0.22	South Asia India Bengali	East Asia China Tujia	0.45	Mainland Southeast Asia Cambodia Cambodian	East Asia China Han NChina	0.06	11.63		0.36	Mainland Southeast Asia Cambodia Cambodian	East Asia China Tujia	69.99		0.21	South Asia India Bengali	East Asia China Tujia
8	South Asia India Garo	1	1D	1D	0.01	0.35	0.98	1.00	24.52	13.24-48.47	0.29	South Asia India Bengali	East Asia China Tujia	0.31	Mainland Southeast Asia Cambodia Cambodian	East Asia China Han	0.02	13.16		0.40	Mainland Southeast Asia Cambodia Cambodian	East Asia China Tujia	66.09		0.28	South Asia India Bengali	East Asia China Tujia
8	South Asia India Jamatia	0	1D	1D	0.01	0.96	1.00	1.00	31.89	26.30-40.36	0.25	South Asia India Bengali	East Asia China Tujia	0.48	Mainland Southeast Asia Cambodia Cambodian	East Asia China Tu	0.14	18.74		0.33	South Asia India Bengali	East Asia China Tujia	63.57		0.25	South Asia India Bengali	East Asia China Tujia
8	South Asia India Jamatia	1	1D	1D	0.01	0.93	1.00	1.00	33.27	28.42-40.69	0.24	South Asia Bangladesh Bengali	East Asia China Tujia	0.46	Mainland Southeast Asia Cambodia Cambodian	East Asia China Tu	0.09	22.74		0.22	South Asia India Bengali	East Asia China Tujia	83.06		0.29	South Asia India Bengali	East Asia China Tujia
8	South Asia India Khasi	0	1D	1D	0.01	0.89	1.00	1.00	15.40	10.98-20.49	0.38	South Asia Bangladesh Bengali	Mainland Southeast Asia Cambodia Cambodian	0.32	East Asia China Tu	Mainland Southeast Asia Cambodia Cambodian	0.19	1.00		0.48	Mainland Southeast Asia Cambodia Cambodian	South Asia India Bengali	25.38		0.4	South Asia India Bengali	Mainland Southeast Asia Cambodia Cambodian
8	South Asia India Khasi	1	1D	1D	0.01	0.67	1.00	1.00	14.76	9.44-20.96	0.38	South Asia Bangladesh Bengali	Mainland Southeast Asia Cambodia Cambodian	0.40	East Asia China Tu	Mainland Southeast Asia Cambodia Cambodian	0.06	1.00		0.45	East Asia China Tujia	South Asia India Bengali	23.67		0.41	South Asia India Bengali	Mainland Southeast Asia Cambodia Cambodian
8	South Asia India Manipuri Brahmin	0	2D	2D	0.01	0.99	1.00	1.00	13.29	11.18-15.30	0.48	East Asia China Tujia	South Asia India West Bengal Brahmin	0.41	Mainland Southeast Asia Cambodia Cambodian	East Asia China Uygur	0.43	11.46	2.00-29.46	0.46	East Asia China Tujia	South Asia Bangladesh Bengali	63.48	45.81-82.48	0.31	East Asia China Tujia	South Asia India Bengali
8	South Asia India Manipuri Brahmin	1	1D	1D	0.01	0.99	1.00	1.00	13.24	11.00-15.44	0.47	East Asia China Tujia	South Asia India West Bengal Brahmin	0.39	Mainland Southeast Asia Cambodia Cambodian	East Asia China Uygur	0.31	11.7		0.47	East Asia China Tujia	South Asia Bangladesh Bengali	63.91		0.28	East Asia China Tujia	South Asia India Bengali
8	South Asia India Tharu	0	2D	2D	0.01	0.94	1.00	1.00	41.69	32.38-56.27	0.48	South Asia Bangladesh Bengali	East Asia China Tu	0.39	East Asia China Uygur	East Asia China Han	0.58	4.79	1.67-29.12	0.22	East Asia China Tu	South Asia India Bengali	58.57	42.90-72.57	0.5	East Asia China Han NChina	South Asia Bangladesh Bengali
8	South Asia India Tharu	1	1D	1D	0.01	0.92	1.00	1.00	43.75	33.67-54.37	0.48	South Asia Bangladesh Bengali	East Asia China Tu	0.34	East Asia China Tu	East Asia China Tu	0.30	7.33		0.22	East Asia China Tu	South Asia India Bengali	63.69		0.5	South Asia Bangladesh Bengali	East Asia China Han NChina
8	South Asia India Tripuri	0	1D	1D	0.01	0.93	1.00	1.00	28.10	19.40-36.70	0.23	South Asia Bangladesh Bengali	East Asia China Tujia	0.47	Mainland Southeast Asia Cambodia Cambodian	East Asia China Tu	0.33	11.9		0.34	South Asia India Bengali	East Asia China Tujia	70.5		0.25	South Asia India Bengali	East Asia China Tujia
8	South Asia India Tripuri	1	1D	1D	0.01	0.92	1.00	1.00	29.54	20.66-37.23	0.22	South Asia Bangladesh Bengali	East Asia China Tujia	0.46	Mainland Southeast Asia Cambodia Cambodian	East Asia China Tu	0.18	14.62		0.32	South Asia India Bengali	East Asia China Tujia	77.82		0.26	South Asia India Bengali	East Asia China Tujia

7	Central Asia Afghanistan	0	2D	2D	0.01	0.97	1.00	1.00	19.63	15.87-23.46	0.22	East Asia China Mongolian	South Asia Pakistan Pathan	0.34	Middle East Turkey Turks	South Asia Pakistan Pathan	0.69	2.32	2.06-21.32	0.44	Mainland Southeast Asia Myanmar Burmese	South Asia Pakistan Pathan	29.53	17.86-38.86	0.22	East Asia China Mongolian	South Asia Pakistan Pathan
7	Central Asia Afghanistan	1	2D	2D	0.01	0.97	1.00	1.00	19.65	13.86-23.21	0.22	East Asia China Mongolian	South Asia Pakistan Pathan	0.34	Middle East Turkey Turks	South Asia Pakistan Pathan	0.60	1.99	1.23-30.99	0.44	Mainland Southeast Asia Myanmar Burmese	South Asia Pakistan Pathan	29.24	6.57-47.91	0.22	East Asia China Mongolian	South Asia Pakistan Pathan
7	Central Asia Uzbekstan Uzbek	0	1D	1D	0.01	0.99	1.00	1.00	19.86	15.73-23.40	0.42	East Asia China Mongolian	Middle East Turkey Turks	0.31	South Asia India Bengali	Middle East Turkey Turks	0.20	15.46		0.42	East Asia China Mongolian	Middle East Turkey Turks	47.83		0.5	East Asia China Mongolian	Middle East Turkey Turks
7	Central Asia Uzbekstan Uzbek	1	1D	1D	0.01	0.98	1.00	1.00	19.82	16.90-24.33	0.42	East Asia China Mongolian	Middle East Turkey Turks	0.32	South Asia India Bengali	Middle East Turkey Turks	0.15	16.4		0.40	East Asia China Mongolian	Middle East Turkey Turks	56.21		0.5	Mainland Southeast Asia Myanmar Burmese	Middle East Turkey Turks
7	East Asia China Oroqen	0	MW	MW	0.01	0.27	0.63	1.00	17.15	7.27-27.69	0.16	East Asia China Hezhen	East Asia China Daur	0.09	South Asia India Bengali	East Asia China Daur	0.03	6.71		0.43	East Asia China Mongolian	East Asia China Daur	14.72		0.36	East Asia China Hezhen	East Asia China Daur
7	East Asia China Oroqen	1	MW	MW	0.01	0.11	0.75	1.00	24.35	4.71-43.14	0.24	East Asia China Hezhen	East Asia China Daur	0.19	Mainland Southeast Asia Myanmar Burmese	East Asia China Daur	0.04	4.58		0.30	East Asia China Daur	East Asia China Daur	13.89		0.33	East Asia China Hezhen	East Asia China Daur
7	East Asia China Uygur	0	1D	1D	0.01	0.98	1.00	1.00	23.37	18.71-27.57	0.49	Middle East Turkey Turks	East Asia China Mongolian	0.42	South Asia India Bengali	East Asia China Mongolian	0.17	1.00		0.19	North America USA CEU	East Asia China Mongolian	25.17		0.49	Middle East Turkey Turks	East Asia China Mongolian
7	East Asia China Uygur	1	1D	1D	0.01	0.97	1.00	1.00	23.85	20.15-27.29	0.49	Middle East Turkey Turks	East Asia China Mongolian	0.41	South Asia India Bengali	East Asia China Mongolian	0.06	14.79		0.15	North America USA CEU	East Asia China Mongolian	27.12		0.49	Middle East Turkey Turks	East Asia China Mongolian
7	North Asia Russia Yakut	0	2D	2D	0.01	0.95	0.99	1.00	11.59	8.13-14.28	0.11	Caucasus Russia Lezgin	East Asia China Mongolian	0.23	East Asia China Hezhen	Mainland Southeast Asia Myanmar Burmese	0.49	3.52	1.34-22.52	0.07	South Asia India Gujarat Brahmin	East Asia China Mongolian	36.16	21.49-54.16	0.1	Caucasus Russia Lezgin	East Asia China Mongolian
7	North Asia Russia Yakut	1	2D	2D	0.01	0.93	1.00	1.00	15.18	10.03-20.96	0.11	Caucasus Russia Lezgin	East Asia China Mongolian	0.25	East Asia China Hezhen	Mainland Southeast Asia Myanmar Burmese	0.48	3.00	2.26-25.33	0.06	Caucasus Russia Lezgin	East Asia China Mongolian	38.59	25.26-58.59	0.12	Caucasus Russia Lezgin	East Asia China Daur
7	South Asia India Indian Telugu UK	0	1D	1D	0.01	0.94	0.98	1.00	9.27	7.50-16.01	0.28	South Asia India Khasi	South Asia India Maratha	0.32	South Asia India Maratha	South Asia India Bengali	0.29	4.31		0.22	South Asia India Khasi	South Asia India Maratha	57.61		0.39	South Asia India Khasi	South Asia India West Bengal Brahmin
7	South Asia India Indian Telugu UK	1	MW	MW	0.01	0.85	0.97	1.00	9.88	7.68-13.75	0.28	South Asia India Khasi	South Asia India Maratha	0.31	South Asia India Maratha	South Asia India Bengali	0.17	4.87		0.27	South Asia India Khasi	South Asia India Maratha	65.9		0.4	South Asia India Khasi	South Asia India West Bengal Brahmin
7	South Asia Pakistan Hazara	0	1D	1D	0.01	0.99	1.00	1.00	22.22	18.53-24.06	0.50	East Asia China Mongolian	Middle East Turkey Turks	0.48	East Asia China Mongolian	South Asia India Bengali	0.12	18.91		0.50	East Asia China Mongolian	South Asia Pakistan Pathan	69.1		0.37	Middle East Turkey Turks	East Asia China Mongolian
7	South Asia Pakistan Hazara	1	1D	1D	0.01	0.99	1.00	1.00	22.63	19.41-24.77	0.50	East Asia China Mongolian	Middle East Turkey Turks	0.48	East Asia China Mongolian	South Asia India Bengali	0.14	17.09		0.48	East Asia China Mongolian	South Asia Pakistan Pathan	26.58		0.5	East Asia China Mongolian	South Asia Pakistan Pathan
9	South Asia India Bonda	0	1D	1D	0.01	0.49	1.00	1.00	50.42	21.55-71.78	0.45	Mainland Southeast Asia Cambodia Cambodian	South Asia India Bengali	0.31	South Asia India Ho	South Asia India Khasi	0.15	1.00		0.42	South Asia India Khasi	South Asia India Ho	52.26		0.49	Mainland Southeast Asia Cambodia Cambodian	South Asia India Bengali
9	South Asia India Bonda	1	1D	1D	0.01	0.18	1.00	1.00	51.75	13.85-101.00	0.45	Mainland Southeast Asia Cambodia Cambodian	South Asia India Bengali	0.48	South Asia India Khasi	South Asia India Khasi	0.05	1.00		0.29	South Asia India Palian	South Asia India Khasi	59.19		0.45	Mainland Southeast Asia Cambodia Cambodian	South Asia India Bengali
9	South Asia India Gond	0	2D	2D	0.01	0.83	0.98	1.00	32.28	26.26-43.90	0.26	South Asia India Khasi	South Asia India Maratha	0.44	South Asia India Maratha	South Asia India Bengali	0.40	4.43	2.00-17.43	0.37	South Asia India Maratha	South Asia India Bengali	54.26	32.59-71.93	0.25	Mainland Southeast Asia Cambodia Cambodian	South Asia India Maratha
9	South Asia India Gond	1	1D	1D	0.01	0.78	0.99	1.00	39.26	34.63-52.76	0.26	South Asia India Khasi	South Asia India Maratha	0.24	South Asia India Maratha	South Asia India Bengali	0.12	10.22		0.44	South Asia India Maratha	South Asia India Bengali	67.01		0.25	Mainland Southeast Asia Cambodia Cambodian	South Asia India Maratha
9	South Asia India Jarawa	0	U	U	0.01	0.32	0.93	0.97	6.01	6.14-57.23	0.41	South Asia India Khasi	South Asia India Khasi	0.43	South Asia India Khasi	South Asia India Khasi	0.16	1.00		0.39	South Asia India Khasi	South Asia India Khasi	106.08		0.48	Mainland Southeast Asia Cambodia Cambodian	South Asia India Bengali
9	South Asia India Jarawa	1	U	U	0.01	0.08	0.94	0.96	216.17	7.15-264.31	0.37	Mainland Southeast Asia Cambodia Cambodian	South Asia India Bengali	0.28	South Asia India Ho	South Asia India Khasi	0.06	1.00		0.40	South Asia India Khasi	South Asia India Khasi	28.97		0.5	South Asia India Khasi	South Asia India Khasi
9	South Asia India Onge	0	U	U	0.01	0.24	0.94	0.98	31.45	5.55-42.82	0.44	South Asia India Bengali	South Asia India Khasi	0.36	South Asia India Khasi	South Asia India Khasi	0.16	1.00		0.33	South Asia India Khasi	South Asia India Khasi	78.05		0.49	South Asia India Khasi	South Asia India Bengali
9	South Asia India Onge	1	U	U	0.01	0.079	0.89	0.91	42.82	2.89-157.28	0.41	South Asia India Bengali	South Asia India Khasi	0.5	South Asia India Khasi	South Asia India Khasi	0.054	1.02		0.43	South Asia India Khasi	South Asia India Khasi	42.95		0.48	South Asia India Khasi	South Asia India Khasi
9	South Asia India Paniya	0	U	1D	0.01	0.52	1.00	1.00	17.47	11.13-22.52	0.41	South Asia India Palian	South Asia India Kadar	0.45	South Asia India Kadar	South Asia India Kadar	0.23	2.54		0.40	South Asia India Kadar	South Asia India Kadar	29.43		0.45	South Asia India Kadar	South Asia India Palian

9	South Asia India Paniya	1	U	1D	0.01	0.46	1.00	1.00	57.94	35.86-91.25	0.33	South Asia India Kadar	South Asia India Palian	0.40	South Asia India Irula	South Asia India Kadar	0.06	1.00		0.39	South Asia India Kadar	South Asia India Kadar	48.74		0.4	South Asia India Kadar	South Asia India Palian
9	South Asia India Pulliyar	0	1D	1D	0.01	0.57	1.00	1.00	27.15	18.29-36.70	0.50	South Asia India Kadar	South Asia India Maratha	0.29	South Asia India Kadar	South Asia India Kadar	0.19	1.00		0.49	South Asia India Kadar	South Asia India Kadar	29.81		0.47	South Asia India Kadar	South Asia India Palian
9	South Asia India Pulliyar	1	1D	1D	0.01	0.39	1.00	1.00	31.84	17.47-44.98	0.47	South Asia India Maratha	South Asia India Kadar	0.49	South Asia India Kadar	South Asia India Kadar	0.04	11.8		0.46	South Asia India Kadar	South Asia India Kadar	32.64		0.49	South Asia India Palian	South Asia India Kadar
10	South Asia India Birhor	0	1D	1D	0.01	0.70	1.00	1.00	59.54	41.25-74.97	0.42	Mainland Southeast Asia Cambodia Cambodian	South Asia India Maratha	0.48	South Asia India Chamar	South Asia India Gond	0.15	1.00		0.42	South Asia India Maratha	South Asia India Gond	75.57		0.4	Mainland Southeast Asia Cambodia Cambodian	South Asia India Maratha
10	South Asia India Birhor	1	1D	1D	0.01	0.56	1.00	1.00	63.74	45.92-85.99	0.47	South Asia India Khasi	South Asia India Maratha	0.47	South Asia India Chamar	South Asia India Gond	0.07	1.00		0.35	South Asia India Chamar	South Asia India Gond	75.77		0.42	Mainland Southeast Asia Cambodia Cambodian	South Asia India Maratha
10	South Asia India Ho	0	1D	1D	0.01	0.88	1.00	1.00	58.12	44.83-69.82	0.40	Mainland Southeast Asia Cambodia Cambodian	South Asia India Maratha	0.43	South Asia India Gond	South Asia India Bengali	0.16	4.03		0.40	South Asia India Gond	South Asia India Khasi	65.26		0.42	Mainland Southeast Asia Cambodia Cambodian	South Asia India Maratha
10	South Asia India Ho	1	1D	1D	0.01	0.81	1.00	1.00	67.41	57.15-78.39	0.42	Mainland Southeast Asia Cambodia Cambodian	South Asia India Maratha	0.45	South Asia India Gond	South Asia India Bengali	0.06	1.00		0.42	South Asia India Gond	South Asia India Khasi	72.44		0.41	Mainland Southeast Asia Cambodia Cambodian	South Asia India Maratha
10	South Asia India Irula	0	1D	1D	0.01	0.51	0.99	1.00	11.06	4.91-26.26	0.44	South Asia India Palian	South Asia India Paniya	0.44	South Asia India Palian	South Asia India Palian	0.13	2.64		0.19	South Asia India Paniya	South Asia India Palian	48.3		0.4	South Asia India Paniya	South Asia India Palian
10	South Asia India Irula	1	1D	1D	0.01	0.30	1.00	1.00	59.01	26.26-90.07	0.35	South Asia India Paniya	South Asia India Palian	0.30	South Asia India Paniya	South Asia India Palian	0.08	1.00		0.31	South Asia India Paniya	South Asia India Palian	65.54		0.3	South Asia India Paniya	South Asia India Palian
10	South Asia India Kadar	0	U	MW	0.01	0.55	0.90	1.00	10.14	4.24-16.22	0.27	South Asia India Palian	South Asia India Paniya	0.48	South Asia India Palian	South Asia India Paniya	0.17	6.09		0.33	South Asia India Palian	South Asia India Paniya	67.06		0.26	South Asia India Paniya	South Asia India Palian
10	South Asia India Kadar	1	U	MW	0.01	0.38	0.94	1.00	63.74	31.84-96.06	0.37	South Asia India Paniya	South Asia India Palian	0.39	South Asia India Paniya	South Asia India Palian	0.06	1.00		0.31	South Asia India Paniya	South Asia India Palian	71.6		0.34	South Asia India Paniya	South Asia India Palian
10	South Asia India Korva	0	1D	1D	0.01	0.79	1.00	1.00	60.08	45.29-75.37	0.46	Mainland Southeast Asia Cambodia Cambodian	South Asia India Maratha	0.44	South Asia India Bengali	South Asia India Gond	0.10	3.4		0.27	South Asia India Gond	South Asia India Gond	71.32		0.48	Mainland Southeast Asia Cambodia Cambodian	South Asia India Chamar
10	South Asia India Korva	1	1D	1D	0.01	0.66	1.00	1.00	63.00	48.31-78.39	0.50	South Asia India Maratha	South Asia India Khasi	0.31	South Asia India Bengali	South Asia India Gond	0.04	1.00		0.32	South Asia India Gond	South Asia India Gond	58.95		0.48	South Asia India Maratha	South Asia India Khasi
10	South Asia India Santal	0	1D	1D	0.01	0.80	1.00	1.00	63.74	49.44-73.16	0.34	Mainland Southeast Asia Cambodia Cambodian	South Asia India Maratha	0.42	South Asia India Gond	South Asia India Bengali	0.14	1.00		0.43	South Asia India Khasi	South Asia India Bengali	78.96		0.34	Mainland Southeast Asia Cambodia Cambodian	South Asia India Maratha
10	South Asia India Santal	1	1D	1D	0.01	0.70	1.00	1.00	71.19	58.29-89.48	0.36	Mainland Southeast Asia Cambodia Cambodian	South Asia India Maratha	0.44	South Asia India Gond	South Asia India Bengali	0.09	1.00		0.26	South Asia India Gond	South Asia India Bengali	79.25		0.35	Mainland Southeast Asia Cambodia Cambodian	South Asia India Maratha
11	South Asia Bangladesh Bengali	0	1D	1D	0.01	0.77	1.00	1.00	47.58	36.91-57.94	0.17	South Asia India Khasi	South Asia India Kshatriya	0.46	South Asia India Gujarat Brahmin	South Asia India Muslim	0.06	1.65		0.39	South Asia India Muslim	South Asia India Muslim	44.59		0.17	South Asia India Khasi	South Asia India Kshatriya
11	South Asia Bangladesh Bengali	1	1D	1D	0.01	0.56	1.00	1.00	45.92	35.04-62.26	0.16	Mainland Southeast Asia Cambodia Cambodian	South Asia India Kshatriya	0.42	South Asia India Muslim	South Asia India West Bengal Brahmin	0.06	2.11		0.35	South Asia India West Bengal Brahmin	South Asia India Muslim	37.51		0.15	Mainland Southeast Asia Cambodia Cambodian	South Asia India Kshatriya
11	South Asia India Bengali	0	1D	1D	0.01	0.92	1.00	1.00	43.44	34.53-45.14	0.19	South Asia India Khasi	South Asia India Kshatriya	0.40	South Asia India West Bengal Brahmin	South Asia India Muslim	0.26	1.00		0.30	South Asia India West Bengal Brahmin	South Asia India Muslim	52.17		0.21	South Asia India Khasi	South Asia India Kshatriya
11	South Asia India Bengali	1	1D	1D	0.01	0.89	1.00	1.00	46.87	40.52-54.12	0.20	South Asia India Khasi	South Asia India Kshatriya	0.48	South Asia India Muslim	South Asia India West Bengal Brahmin	0.08	1.00		0.43	South Asia India West Bengal Brahmin	South Asia India Muslim	51.71		0.21	South Asia India Khasi	South Asia India Kshatriya
11	South Asia India Chamar	0	U	U	0.01	0.34	0.95	0.96	22.03	10.03-57.94	0.36	South Asia India Uttar Pradesh Scheduled Caste	South Asia India Kshatriya	0.48	South Asia India Muslim	South Asia India Uttar Pradesh Scheduled Caste	0.08	1.65		0.47	South Asia India Muslim	South Asia India Muslim	14.95		0.35	South Asia India Gond	South Asia India Kshatriya
11	South Asia India Chamar	1	U	U	0.01	0.11	0.78	0.86	13.47	3.42-38.40	0.38	South Asia India Uttar Pradesh Scheduled Caste	South Asia India West Bengal Brahmin	0.48	South Asia India Kshatriya	South Asia India Muslim	0.03	1.85		0.42	South Asia India Muslim	South Asia India Muslim	8.34		0.48	South Asia India Uttar Pradesh Scheduled Caste	South Asia India Kshatriya
11	South Asia India Chenchus	0	U	U	0.01	0.65	0.75	0.89	15.60	7.70-32.82	0.26	South Asia India Khasi	South Asia India Kshatriya	0.40	South Asia India West Bengal Brahmin	South Asia India Punjabi	0.11	1.1		0.50	South Asia India Kshatriya	South Asia India Muslim	36.79		0.42	South Asia India Gond	South Asia India Punjabi

11	South Asia India Chenchus	1	U	U	0.01	0.09	0.70	0.82	3.15	1.15-53.10	0.49	South Asia India Muslim	South Asia India Punjabi	0.48	South Asia India Punjabi	South Asia India Kshatriya	0.08	1.00		0.45	South Asia India Muslim	South Asia India Punjabi	58.1		0.35	South Asia Pakistan Punjabi	South Asia India Muslim
11	South Asia India Hakkipikki	0	U	U	0.01	0.25	0.94	0.96	18.56	4.49-43.44	0.27	South Asia India North Kannadi	South Asia India Kshatriya	0.42	South Asia India Gond	South Asia India Kshatriya	0.08	1.00		0.49	South Asia India Kshatriya	South Asia India Muslim	38.1		0.38	South Asia India North Kannadi	South Asia India Kshatriya
11	South Asia India Hakkipikki	1	U	U	0.01	0.06	0.86	0.88	1.00	1.00-64.48	0.31	South Asia India North Kannadi	South Asia India Kshatriya	0.41	South Asia India Muslim	South Asia India Kshatriya	0.05	1.00		0.22	South Asia India North Kannadi	South Asia India Kshatriya	45.16		0.29	South Asia India North Kannadi	South Asia India Muslim
11	South Asia India Kol	0	U	U	0.01	0.64	0.91	0.96	38.61	31.06-44.05	0.47	South Asia India Muslim	South Asia India Gujarat Brahmin	0.25	South Asia India West Bengal Brahmin	South Asia India Muslim	0.17	1.31		0.32	South Asia India West Bengal Brahmin	South Asia India Muslim	55.48		0.43	South Asia India Gujarat Brahmin	South Asia India Muslim
11	South Asia India Kol	1	U	U	0.01	0.48	0.88	0.92	54.12	43.75-79.77	0.50	South Asia India Muslim	South Asia India Gujarat Brahmin	0.29	South Asia India West Bengal Brahmin	South Asia India Muslim	0.06	1.00		0.29	South Asia India Muslim	South Asia India Kshatriya	63.94		0.45	South Asia Pakistan Pathan	South Asia India Muslim
11	South Asia India Maratha	0	U	U	0.01	0.33	0.93	0.95	48.47	13.10-84.84	0.50	South Asia India Gond	South Asia India Brahmin	0.34	South Asia India Muslim	South Asia India West Bengal Brahmin	0.07	1.00		0.32	South Asia India Kshatriya	South Asia India Muslim	62.64		0.49	South Asia India Gond	South Asia India Brahmin
11	South Asia India Maratha	1	U	U	0.01	0.16	0.84	0.87	76.47	29.54-99.75	0.45	South Asia India Gond	South Asia India Brahmin	0.41	South Asia India West Bengal Brahmin	South Asia India Muslim	0.04	1.00		0.35	South Asia India Kshatriya	South Asia India Muslim	76.27		0.42	South Asia India Gond	South Asia India Brahmin
11	South Asia India Palian	0	U	U	0.01	0.51	0.85	0.92	58.65	33.02-62.63	0.48	South Asia India Gujarat Brahmin	South Asia India Gond	0.44	South Asia India West Bengal Brahmin	South Asia India Muslim	0.32	1.00		0.49	South Asia India Muslim	South Asia India Muslim	73.13		0.48	South Asia India Gujarat Brahmin	South Asia India Gond
11	South Asia India Palian	1	U	U	0.01	0.36	0.95	0.97	97.29	82.56-119.95	0.49	South Asia India Gujarat Brahmin	South Asia India Gond	0.50	South Asia India Kshatriya	South Asia India Muslim	0.05	1.00		0.49	South Asia India Muslim	South Asia India Muslim	70.57		0.46	South Asia India Gujarat Brahmin	South Asia India Gond
11	South Asia India Piramalai Kallar	0	U	U	0.01	0.26	0.63	0.81	38.40	1.78-88.89	0.44	South Asia India Muslim	South Asia India Kshatriya	0.47	South Asia India Muslim	South Asia India Kshatriya	0.15	1.00		0.41	South Asia India Muslim	South Asia India Kshatriya	50.24		0.44	South Asia India Muslim	South Asia India Kshatriya
11	South Asia India Piramalai Kallar	1	U	U	0.01	0.08	0.61	0.70	34.63	3.42-75.37	0.34	South Asia India Gond	South Asia India Kshatriya	0.49	South Asia India Muslim	South Asia India Kshatriya	0.03	1.00		0.43	South Asia India Gujarat Brahmin	South Asia India Muslim	24.63		0.42	South Asia India Gond	South Asia India Kshatriya
11	South Asia India Sakilli	0	U	U	0.01	0.27	0.75	0.81	12.29	2.66-60.80	0.48	South Asia India Gond	South Asia India Kshatriya	0.39	South Asia India Muslim	South Asia India Muslim	0.12	1.00		0.46	South Asia India Muslim	South Asia India Kshatriya	69.43		0.47	South Asia India Gond	South Asia India Gujarat Brahmin
11	South Asia India Sakilli	1	U	U	0.01	0.05	0.86	0.88	115.22	1.42-161.49	0.29	South Asia Pakistan Balochi	South Asia India Gond	0.35	South Asia India Kshatriya	South Asia India Muslim	0.04	1.00		0.31	South Asia India Gujarat Brahmin	South Asia India Muslim	124.54		0.44	South Asia India Gond	South Asia India Gujarat Brahmin
11	South Asia India Velama	0	MW	MW	0.01	0.25	0.97	1.00	31.06	11.25-42.83	0.43	South Asia India Indian	South Asia India Kshatriya	0.36	South Asia India Gond	South Asia India Gujarat Brahmin	0.06	1.00		0.49	South Asia India Muslim	South Asia India Kshatriya	36.97		0.48	South Asia India Indian	South Asia India Iyer
11	South Asia India Velama	1	1D	1D	0.01	0.18	0.98	1.00	37.54	7.20-55.84	0.50	South Asia India Indian	South Asia India Iyer	0.38	South Asia India Gond	South Asia India Gujarat Brahmin	0.05	1.00		0.49	South Asia India Kshatriya	South Asia India Gujarat Brahmin	38.26		0.44	South Asia India Iyer	South Asia India Indian
11	South Asia SriLanka Sri Lanka UK	0	U	U	0.01	0.53	0.81	0.98	63.00	29.53-77.47	0.30	South Asia India Gond	South Asia India Kshatriya	0.48	South Asia India Kshatriya	South Asia India Muslim	0.56	1.00		0.17	South Asia India Gond	South Asia India Muslim	95.14		0.38	South Asia India Gond	South Asia India Gujarat Brahmin
11	South Asia SriLanka Sri Lanka UK	1	U	U	0.01	0.48	0.91	0.98	75.93	53.10-99.75	0.39	South Asia India Gond	South Asia India Gujarat Brahmin	0.42	South Asia India Muslim	South Asia India Kshatriya	0.18	1.00		0.34	South Asia India Muslim	South Asia India Kshatriya	100.88		0.42	South Asia India Gond	South Asia India Gujarat Brahmin
12	South Asia India Brahmin	0	1D	1D	0.01	0.78	1.00	1.00	57.76	47.34-67.31	0.37	Middle East Turkey Turks	South Asia India Maratha	0.30	South Asia India Bengali	South Asia India Maratha	0.08	1.00		0.45	South Asia India Maratha	South Asia India Bengali	59.34		0.39	Middle East Turkey Turks	South Asia India Maratha
12	South Asia India Brahmin	1	1D	1D	0.01	0.72	1.00	1.00	58.12	46.08-72.57	0.37	Middle East Turkey Turks	South Asia India Maratha	0.45	South Asia India Bengali	South Asia India Maratha	0.04	1.00		0.46	South Asia India Maratha	South Asia India Bengali	58.19		0.37	Middle East Turkey Turks	South Asia India Maratha
12	South Asia India Brahmins from Uttar Pradesh	0	1D	1D	0.01	0.46	1.00	1.00	66.93	43.14-97.29	0.43	Middle East Turkey Turks	South Asia India Maratha	0.37	South Asia India Bengali	South Asia India Maratha	0.08	1.00		0.49	South Asia Bangladesh Bengali	South Asia India Maratha	67.35		0.44	Middle East Turkey Turks	South Asia India Maratha
12	South Asia India Brahmins from Uttar Pradesh	1	1D	1D	0.01	0.29	0.99	1.00	61.17	37.97-99.75	0.47	South Asia Pakistan Pathan	South Asia India Maratha	0.42	South Asia India Bengali	South Asia India Maratha	0.05	1.00		0.49	South Asia India Maratha	South Asia Pakistan Pathan	64.27		0.46	Middle East Turkey Turks	South Asia India Maratha
12	South Asia India Cochin Jews	0	1D	1D	0.01	0.81	1.00	1.00	16.11	8.55-25.47	0.22	Middle East Turkey Sephardic Jews	South Asia India Maratha	0.34	South Asia India Bengali	South Asia India Maratha	0.21	1.19		0.23	Middle East Turkey Sephardic Jews	South Asia India Maratha	34.39		0.25	Middle East Turkey Sephardic Jews	South Asia India Maratha
12	South Asia India Cochin Jews	1	1D	1D	0.01	0.53	0.99	1.00	13.03	7.57-20.14	0.25	Middle East Lebanon Lebanese	South Asia India Maratha	0.27	South Asia India Bengali	South Asia India Maratha	0.07	3.01		0.28	Middle East Turkey Turks	South Asia India Maratha	43.67		0.3	Middle East Turkey Sephardic Jews	South Asia India Palian
12	South Asia India Dharkar	0	1D	1D	0.01	0.67	0.99	1.00	39.70	24.52-55.84	0.46	South Asia Pakistan Pathan	South Asia India Maratha	0.39	South Asia India Bengali	South Asia India Maratha	0.11	1.9		0.39	South Asia Pakistan Sindhi	South Asia India Bengali	53.98		0.46	South Asia Pakistan Pathan	South Asia India Maratha
12	South Asia India Dharkar	1	1D	1D	0.01	0.58	0.99	1.00	43.44	25.38-56.53	0.43	South Asia Pakistan Pathan	South Asia India Maratha	0.40	South Asia India Bengali	South Asia India Maratha	0.09	2.7		0.38	South Asia Pakistan Sindhi	South Asia India Chamar	60.42		0.45	South Asia Pakistan Pathan	South Asia India Maratha
12	South Asia India Gujarat Brahmin	0	1D	1D	0.01	0.67	1.00	1.00	68.27	51.25-83.70	0.40	Middle East Turkey Turks	South Asia India Maratha	0.50	South Asia Pakistan Sindhi	South Asia India Bengali	0.08	1.00		0.36	South Asia India Maratha	South Asia Pakistan Pathan	72.43		0.42	Middle East Turkey Turks	South Asia India Maratha
12	South Asia India Gujarat Brahmin	1	1D	1D	0.01	0.58	1.00	1.00	67.51	54.46-88.31	0.40	Middle East Turkey Turks	South Asia India Maratha	0.47	South Asia India Bengali	South Asia Pakistan Sindhi	0.04	1.00		0.48	South Asia India Maratha	South Asia Pakistan Sindhi	67.32		0.4	Middle East Turkey Turks	South Asia India Maratha

12	South Asia India Gujarati	0	1D	1D	0.01	0.72	0.99	1.00	60.08	44.83-70.60	0.33	Caucasus Russia Lezgin	South Asia India Maratha	0.45	South Asia Pakistan Sindhi	South Asia India Bengali	0.05	1.00		0.48	South Asia India Maratha	South Asia India Maratha	65.53		0.34	Caucasus Russia Lezgin	South Asia India Chamar
12	South Asia India Gujarati	1	1D	1D	0.01	0.63	0.98	1.00	67.51	53.43-75.12	0.35	Caucasus Russia Lezgin	South Asia India Chamar	0.49	South Asia India Maratha	South Asia India Bengali	0.03	24.1		0.39	South Asia India Maratha	South Asia India Maratha	62.73		0.32	Caucasus Russia Lezgin	South Asia India Maratha
12	South Asia India Iyer	0	1D	1D	0.01	0.62	1.00	1.00	92.44	68.66-126.86	0.34	North America USA CEU	South Asia India Palian	0.45	South Asia India Bengali	South Asia India Maratha	0.09	1.00		0.41	South Asia India Maratha	South Asia India Maratha	89.00		0.34	North America USA CEU	South Asia India Palian
12	South Asia India Iyer	1	1D	1D	0.01	0.54	1.00	1.00	102.88	77.56-120.63	0.36	North America USA CEU	South Asia India Palian	0.41	South Asia India Gond	South Asia India Maratha	0.05	1.00		0.44	South Asia India Maratha	South Asia India Maratha	95.09		0.35	North America USA CEU	South Asia India Palian
12	South Asia India Kanjars	0	1D	1D	0.01	0.60	0.99	0.99	38.40	24.52-58.29	0.35	South Asia Pakistan Pathan	South Asia India Maratha	0.48	South Asia India Chamar	South Asia India Maratha	0.09	1.00		0.44	South Asia India Bengali	South Asia India Maratha	36.07		0.33	South Asia Pakistan Pathan	South Asia India Maratha
12	South Asia India Kanjars	1	1D	1D	0.01	0.36	0.98	0.99	39.26	22.27-55.84	0.32	South Asia Pakistan Pathan	South Asia India Maratha	0.42	South Asia India Maratha	South Asia India Bengali	0.03	1.00		0.47	South Asia Bangladesh Bengali	South Asia India Maratha	46.17		0.29	Middle East Turkey Turks	South Asia India Maratha
12	South Asia India Kshatriya	0	1D	1D	0.01	0.46	0.99	1.00	49.28	22.40-75.66	0.42	South Asia Pakistan Pathan	South Asia India Maratha	0.48	South Asia India Maratha	South Asia India Bengali	0.13	4.05		0.33	South Asia Pakistan Pathan	South Asia India Bengali	63.73		0.41	Middle East Turkey Turks	South Asia India Maratha
12	South Asia India Kshatriya	1	1D	1D	0.01	0.22	0.99	1.00	50.75	26.26-90.66	0.45	South Asia Pakistan Pathan	South Asia India Maratha	0.40	South Asia India Maratha	South Asia India Bengali	0.04	5.2		0.40	South Asia Pakistan Pathan	South Asia India Bengali	74.26		0.41	Middle East Turkey Turks	South Asia India Maratha
12	South Asia India Mumbai Jews	0	1D	1D	0.01	0.86	1.00	1.00	26.08	19.97-32.47	0.44	Middle East Lebanon Lebanese	South Asia India Maratha	0.37	South Asia India Maratha	South Asia Pakistan Pathan	0.06	23.98		0.45	Middle East Lebanon Lebanese	South Asia India Maratha	3.22		0.41	South Asia India Maratha	South Asia Pakistan Pathan
12	South Asia India Mumbai Jews	1	1D	1D	0.01	0.57	1.00	1.00	27.51	20.96-35.04	0.44	Middle East Lebanon Lebanese	South Asia India Maratha	0.47	South Asia India Maratha	South Asia Pakistan Pathan	0.05	2.48		0.44	South Asia India Maratha	South Asia India Maratha	23.53		0.45	Middle East Lebanon Lebanese	South Asia India Maratha
12	South Asia India Muslim	0	1D	1D	0.01	0.56	1.00	1.00	36.07	14.61-59.36	0.26	Middle East Turkey Turks	South Asia India Chamar	0.44	South Asia India Maratha	South Asia India Chamar	0.08	7.89		0.38	South Asia India Chamar	South Asia Bangladesh Bengali	57.8		0.25	Middle East Turkey Turks	South Asia India Chamar
12	South Asia India Muslim	1	1D	1D	0.01	0.23	0.98	0.99	18.34	9.33-60.08	0.26	South Asia Pakistan Pathan	South Asia India Chamar	0.36	South Asia India Bengali	South Asia India Chamar	0.04	7.75		0.43	South Asia India Chamar	South Asia India Bengali	79.25		0.32	Middle East Turkey Turks	South Asia India Maratha
12	South Asia India Punjabi	0	1D	1D	0.01	0.81	0.99	1.00	35.86	22.27-43.29	0.41	Middle East Turkey Turks	South Asia India Maratha	0.43	South Asia India Bengali	South Asia Pakistan Sindhi	0.21	8.94		0.38	South Asia Pakistan Pathan	South Asia India Maratha	58.01		0.47	Middle East Turkey Turks	South Asia India Maratha
12	South Asia India Punjabi	1	1D	1D	0.01	0.76	0.99	1.00	35.97	27.24-48.63	0.42	South Asia Pakistan Pathan	South Asia India Maratha	0.41	South Asia India Bengali	South Asia Pakistan Sindhi	0.13	8.71		0.42	South Asia Pakistan Pathan	South Asia India Maratha	53.35		0.47	South Asia Pakistan Pathan	South Asia India Maratha
12	South Asia India Uttar Pradesh Scheduled Caste	0	U	U	0.01	0.34	0.86	0.97	51.75	1.68-79.77	0.38	South Asia Pakistan Pathan	South Asia India Bengali	0.43	South Asia India Bengali	South Asia India Chamar	0.21	1.01		0.35	South Asia India Bengali	South Asia India Maratha	40.52		0.41	South Asia Pakistan Pathan	South Asia India Maratha
12	South Asia India Uttar Pradesh Scheduled Caste	1	U	U	0.01	0.11	0.87	0.92	47.18	8.66-86.56	0.45	South Asia Pakistan Pathan	South Asia India Maratha	0.48	South Asia India Maratha	South Asia India Chamar	0.07	1.00		0.35	South Asia India Bengali	South Asia India Chamar	50.67		0.42	South Asia Pakistan Pathan	South Asia India Maratha
12	South Asia India West Bengal Brahmin	0	MW	MW	0.01	0.80	0.90	1.00	48.47	33.42-58.29	0.50	South Asia India Bengali	South Asia Pakistan Pathan	0.25	East Asia China Uygur	South Asia India Maratha	0.21	1.00		0.41	South Asia India Bengali	South Asia India Maratha	51.78		0.46	South Asia Pakistan Pathan	South Asia India Bengali
12	South Asia India West Bengal Brahmin	1	MW	MW	0.01	0.70	0.92	1.00	50.42	37.44-68.27	0.49	South Asia Pakistan Pathan	South Asia India Bengali	0.29	Central Asia Afghanistan	South Asia India Maratha	0.05	1.00		0.47	South Asia Pakistan Pathan	South Asia India Bengali	52.13		0.48	South Asia Pakistan Pathan	South Asia India Bengali
12	South Asia Pakistan Punjabi	0	1D	1D	0.01	0.68	0.99	1.00	25.91	13.03-39.26	0.39	South Asia Pakistan Pathan	South Asia India Maratha	0.36	South Asia India Maratha	South Asia India Maratha	0.13	3.82		0.43	South Asia Pakistan Pathan	South Asia India Maratha	46.94		0.41	South Asia Pakistan Pathan	South Asia India Maratha
12	South Asia Pakistan Punjabi	1	1D	1D	0.01	0.46	1.00	1.00	29.17	15.60-44.05	0.44	South Asia Pakistan Pathan	South Asia India Maratha	0.38	South Asia India Bengali	South Asia India Maratha	0.06	2.83		0.24	South Asia India Maratha	South Asia Pakistan Pathan	32.82		0.4	South Asia Pakistan Pathan	South Asia India Maratha
13	South Asia India Khatri	0	1D	1D	0.01	0.66	1.00	1.00	68.85	29.54-107.67	0.36	Middle East Turkey Turks	South Asia India Kshatriya	0.25	Central Asia Afghanistan	South Asia India Brahmin	0.10	4.59		0.32	South Asia India Kshatriya	South Asia India Brahmin	94.3		0.38	Middle East Turkey Turks	South Asia India Maratha
13	South Asia India Khatri	1	1D	1D	0.01	0.56	1.00	1.00	65.23	37.97-93.64	0.36	Middle East Turkey Turks	South Asia India Kshatriya	0.40	South Asia India Brahmin	South Asia India Gujarat Brahmin	0.05	1.00		0.44	South Asia India Gujarat Brahmin	South Asia India Brahmin	71.85		0.35	Middle East Turkey Turks	South Asia India Kshatriya
13	South Asia Pakistan Balochi	0	MW	MW	0.01	0.96	0.94	1.00	23.01	17.72-27.02	0.08	South Africa South African Bantu Soweto	South Asia India Brahmin	0.41	South Asia India Gujarat Brahmin	Middle East Turkey Turks	0.25	16.13		0.04	East Africa Kenya BantuKenya	South Asia India Brahmin	51.83		0.44	Middle East Turkey Turks	South Asia India Kshatriya
13	South Asia Pakistan Balochi	1	MW	MW	0.01	0.94	0.93	1.00	23.72	18.18-27.69	0.08	East Africa Kenya BantuKenya	South Asia India Brahmin	0.37	South Asia India Gujarat Brahmin	Middle East Turkey Turks	0.19	14.13		0.05	East Africa Kenya BantuKenya	South Asia India Brahmin	43.67		0.44	Middle East Turkey Turks	South Asia India Kshatriya
13	South Asia Pakistan Brahui	0	2D	2D	0.01	0.97	0.93	1.00	17.47	10.42-22.03	0.07	East Africa Kenya BantuKenya	South Asia India Brahmin	0.43	South Asia India Gujarat Brahmin	Middle East Turkey Turks	0.38	10.19	2.33-37.52	0.04	East Africa Kenya BantuKenya	South Asia India Brahmin	52.01	30.34-60.34	0.39	Middle East Turkey Turks	South Asia India Kshatriya
13	South Asia Pakistan Brahui	1	MW	MW	0.01	0.96	0.94	1.00	17.85	12.29-21.28	0.08	East Africa Kenya BantuKenya	South Asia India Brahmin	0.42	South Asia India Gujarat Brahmin	Middle East Turkey Turks	0.29	10.43		0.04	East Africa Kenya BantuKenya	South Asia India Brahmin	52.06		0.38	Middle East Lebanon Lebanese	South Asia India Kshatriya
13	South Asia Pakistan Burusho	0	1D	1D	0.01	0.93	0.98	1.00	40.25	33.22-49.52	0.12	East Asia China Tu	South Asia India Brahmin	0.32	Middle East Turkey Turks	South Asia India Bengali	0.22	6.56		0.04	East Asia China Han NChina	South Asia India Brahmin	57.38		0.12	East Asia China Tu	South Asia India Brahmin

13	South Asia Pakistan Burusho	1	MW	MW	0.01	0.90	0.97	1.00	43.52	34.43-52.42	0.12	East Asia China Tu	South Asia India Brahmin	0.30	Middle East Turkey Turks	South Asia India Bengali	0.17	5.02		0.29	South Asia India West Bengal Brahmin	South Asia India Brahmin	59.29		0.13	East Asia China Xibo	South Asia India Brahmin
13	South Asia Pakistan Kalash	0	1D	1D	0.01	0.49	1.00	1.00	54.80	28.42-101.00	0.32	Middle East Turkey Turks	South Asia India Brahmin	0.39	South Asia India Brahmin	South Asia India Brahmin	0.10	1.00		0.26	Middle East Turkey Turks	South Asia India Brahmin	76.01		0.35	Middle East Turkey Turks	South Asia India Kshatriya
13	South Asia Pakistan Kalash	1	1D	1D	0.01	0.35	1.00	1.00	65.23	38.18-98.52	0.31	Middle East Turkey Turks	South Asia India Brahmin	0.36	South Asia India Brahmin	South Asia India Brahmin	0.03	1.00		0.28	North America USA CEU	South Asia India Brahmin	72.83		0.32	Middle East Turkey Turks	South Asia India Brahmins from Uttar Pradesh
13	South Asia Pakistan Makrani	0	2D	2D	0.01	0.99	0.99	1.00	15.72	12.38-17.39	0.07	East Africa Kenya BantuKenya	South Asia India Brahmin	0.33	Middle East Lebanon Lebanese	South Asia India Gujarat Brahmin	0.37	7.53	2.14-16.20	0.11	East Africa Kenya BantuKenya	South Asia India Brahmin	26.67	9.00-45.67	0.09	South Africa South African Bantu Soweto	South Asia India Brahmin
13	South Asia Pakistan Makrani	1	2D	2D	0.01	0.99	0.99	1.00	16.01	11.91-17.74	0.07	East Africa Kenya BantuKenya	South Asia India Brahmin	0.31	Middle East Lebanon Lebanese	South Asia India Gujarat Brahmin	0.39	11.65	2.33-23.65	0.09	East Africa Kenya BantuKenya	South Asia India Brahmin	38.98	29.65-59.98	0.2	South Africa South Africa ColouredWellington	South Asia India Brahmin
13	South Asia Pakistan Pathan	0	1D	1D	0.01	0.71	1.00	1.00	23.85	15.30-33.12	0.49	Central Asia Afghanistan	South Asia India Gujarat Brahmin	0.37	Middle East Turkey Turks	South Asia India Brahmin	0.17	9.69		0.50	South Asia India Brahmin	Central Asia Afghanistan	70.67		0.49	Central Asia Afghanistan	South Asia India Kshatriya
13	South Asia Pakistan Pathan	1	1D	1D	0.01	0.63	1.00	1.00	24.95	15.20-37.54	0.48	Central Asia Afghanistan	South Asia India Kshatriya	0.37	Middle East Turkey Turks	South Asia India Brahmin	0.11	9.08		0.40	South Asia India Brahmin	Central Asia Afghanistan	71.69		0.49	South Asia India Kshatriya	Central Asia Afghanistan
13	South Asia Pakistan Sindhi	0	2D	2D	0.01	0.98	1.00	1.00	6.32	4.54-9.89	0.12	North Africa Egypt Egyptian	South Asia India Gujarat Brahmin	0.37	Middle East Turkey Turks	South Asia India Gujarat Brahmin	0.46	5.39	1.33-22.06	0.12	North Africa Egypt Egyptian	South Asia India Gujarat Brahmin	64.72	56.05-74.72	0.25	Middle East Turkey Turks	South Asia India Kshatriya
13	South Asia Pakistan Sindhi	1	2D	2D	0.01	0.97	1.00	1.00	6.26	3.94-9.83	0.11	North Africa Egypt Egyptian	South Asia India Gujarat Brahmin	0.40	South Asia India Brahmin	South Asia India Gujarat Brahmin	0.37	4.53	1.67-29.20	0.10	East Africa Kenya BantuKenya	South Asia India Gujarat Brahmin	41.84	27.84-63.51	0.25	Middle East Turkey Turks	South Asia India Kshatriya
14	Middle East Israel Bedouin	0	1D	1D	0.01	0.94	1.00	1.00	27.49	21.55-34.53	0.43	East Africa Sudan Arab	Middle East Turkey Sephardic Jews	0.36	East Africa Ethiopia Ethiopians	East Africa Sudan Arab	0.27	9.26		0.26	East Africa Sudan Arab	Middle East Turkey Sephardic Jews	46.82		0.45	East Africa Sudan Arab	Middle East Turkey Sephardic Jews
14	Middle East Israel Bedouin	1	1D	1D	0.01	0.93	1.00	1.00	27.40	21.58-33.92	0.44	East Africa Sudan Arab	Middle East Turkey Sephardic Jews	0.30	East Africa Ethiopia Ethiopians	Middle East Turkey Sephardic Jews	0.21	11.28		0.34	East Africa Sudan Arab	Middle East Turkey Sephardic Jews	51.89		0.44	East Africa Sudan Arab	Middle East Turkey Sephardic Jews
14	Middle East Israel Palestinian	0	2D	2D	0.01	0.98	1.00	1.00	27.56	21.18-34.83	0.09	West Africa Nigeria Yoruba	Middle East Turkey Sephardic Jews	0.40	Middle East Turkey Turks	Middle East Turkey Sephardic Jews	0.39	14.32	1.67-21.65	0.04	West Africa Nigeria Yoruba	Middle East Turkey Sephardic Jews	48.55	31.88-69.88	0.1	West Africa Nigeria Yoruba	Middle East Turkey Sephardic Jews
14	Middle East Israel Palestinian	1	1D	1D	0.01	0.98	1.00	1.00	27.17	20.50-30.98	0.09	West Africa Nigeria Yoruba	Middle East Turkey Sephardic Jews	0.40	Middle East Turkey Turks	Middle East Turkey Sephardic Jews	0.35	14.81		0.04	West Africa Nigeria Yoruba	Middle East Turkey Sephardic Jews	49.76		0.1	West Africa Nigeria Yoruba	Middle East Turkey Sephardic Jews
14	Middle East Jordania Jordanian	0	1D	1D	0.01	0.98	1.00	1.00	23.16	18.73-28.19	0.10	East Africa Kenya Turkana	Middle East Turkey Sephardic Jews	0.40	North America USA CEU	Middle East Turkey Sephardic Jews	0.29	3.6		0.18	Middle East Turkey Sephardic Jews	Middle East Turkey Sephardic Jews	27.89		0.1	West Africa Nigeria Yoruba	Middle East Turkey Sephardic Jews
14	Middle East Jordania Jordanian	1	1D	1D	0.01	0.98	1.00	1.00	23.35	19.58-25.84	0.10	West Africa Nigeria Yoruba	Middle East Turkey Sephardic Jews	0.42	North America USA CEU	Middle East Turkey Sephardic Jews	0.25	1.7		0.18	East Africa Sudan Nubian	Middle East Turkey Turks	27.16		0.1	West Africa Nigeria Yoruba	Middle East Turkey Sephardic Jews
14	Middle East Lebanon Lebanese	0	1D	1D	0.01	0.93	0.99	1.00	33.92	21.08-44.75	0.06	West Africa Nigeria Yoruba	Middle East Turkey Turks	0.36	Middle East Turkey Turks	Middle East Turkey Sephardic Jews	0.10	11.73		0.15	Middle East Turkey Sephardic Jews	Middle East Turkey Turks	49.13		0.06	West Africa Nigeria Yoruba	Middle East Turkey Turks
14	Middle East Lebanon Lebanese	1	1D	1D	0.01	0.86	0.99	1.00	33.37	22.82-49.93	0.05	West Africa Nigeria Yoruba	Middle East Turkey Turks	0.41	Middle East Turkey Turks	Middle East Turkey Sephardic Jews	0.08	7.37		0.46	Middle East Turkey Sephardic Jews	Middle East Turkey Turks	44.46		0.05	West Africa Nigeria Yoruba	Middle East Turkey Turks
14	Middle East Saudi Arabia Saudi	0	1D	1D	0.01	0.90	1.00	1.00	17.29	12.57-23.32	0.48	Middle East Turkey Turks	East Africa Sudan Arab	0.33	Middle East Turkey Sephardic Jews	Middle East Turkey Sephardic Jews	0.18	4.77		0.44	East Africa Sudan Arab	Middle East Turkey Sephardic Jews	35.3		0.5	Middle East Turkey Turks	East Africa Sudan Arab
14	Middle East Saudi Arabia Saudi	1	1D	1D	0.01	0.87	1.00	1.00	17.88	10.95-26.79	0.48	Middle East Turkey Turks	East Africa Sudan Arab	0.39	Middle East Turkey Sephardic Jews	Middle East Turkey Sephardic Jews	0.17	3.21		0.40	Middle East Turkey Sephardic Jews	East Africa Sudan Arab	34.29		0.5	Middle East Turkey Turks	East Africa Sudan Arab
14	Middle East Syria Syrians	0	1D	1D	0.01	0.97	0.99	1.00	26.79	19.94-32.92	0.09	West Africa Nigeria Yoruba	Middle East Turkey Turks	0.38	Middle East Turkey Turks	Middle East Turkey Sephardic Jews	0.13	12.92		0.04	Central Africa Gabon Nzebi	Middle East Turkey Turks	41.46		0.1	East Africa Ethiopia Ethiopians	Middle East Turkey Turks
14	Middle East Syria Syrians	1	1D	1D	0.01	0.96	0.99	1.00	27.29	23.10-33.74	0.09	East Africa Kenya Turkana	Middle East Turkey Turks	0.35	Middle East Turkey Turks	Middle East Turkey Sephardic Jews	0.11	16.45		0.03	Central Africa Gabon Nzebi	Middle East Turkey Turks	43.18		0.11	East Africa Ethiopia Ethiopians	Middle East Turkey Turks
14	Middle East Yemen Yemeni	0	2D	2D	0.01	0.96	0.98	1.00	14.25	10.32-19.19	0.21	East Africa Kenya BantuKenya	Middle East Turkey Sephardic Jews	0.48	East Africa Ethiopia Ethiopians	Middle East Turkey Sephardic Jews	0.53	3.66	2.00-27.99	0.21	East Africa Kenya BantuKenya	Middle East Turkey Sephardic Jews	28.38	20.38-51.71	0.23	East Africa Kenya BantuKenya	Middle East Turkey Sephardic Jews
14	Middle East Yemen Yemeni	1	2D	2D	0.01	0.95	0.98	1.00	14.91	11.44-19.02	0.20	East Africa Kenya BantuKenya	Middle East Turkey Sephardic Jews	0.49	East Africa Ethiopia Ethiopians	Middle East Turkey Sephardic Jews	0.42	3.78	1.13-18.78	0.21	East Africa Kenya BantuKenya	Middle East Turkey Sephardic Jews	29.16	7.49-40.16	0.24	East Africa Kenya BantuKenya	Middle East Turkey Sephardic Jews
14	Middle East Yemen Yemenite Jews	0	1D	1D	0.01	0.75	1.00	1.00	37.70	26.08-54.29	0.40	East Africa Ethiopia Ethiopians	Middle East Turkey Sephardic Jews	0.35	Caucasus Georgia Georgian Jews	Middle East Turkey Sephardic Jews	0.14	1.00		0.45	Middle East Turkey Sephardic Jews	East Africa Ethiopia Ethiopians	47.74		0.33	East Africa Ethiopia Ethiopians	Middle East Turkey Sephardic Jews

14	Middle East Yemen Yemenite Jews	1	1D	1D	0.01	0.65	1.00	1.00	40.36	28.98-56.53	0.42	East Africa Ethiopia Ethiopians	Middle East Turkey Sephardic Jews	0.42	Caucasus Georgia Georgian Jews	Middle East Turkey Sephardic Jews	0.07	1.00		0.45	Middle East Turkey Sephardic Jews	Middle East Turkey Sephardic Jews	46.67		0.41	East Africa Ethiopia Ethiopians	Middle East Turkey Sephardic Jews
14	North Africa Egypt Egyptian	0	2D	2D	0.01	0.98	1.00	1.00	22.35	17.31-26.88	0.16	West Africa Nigeria Yoruba	Middle East Turkey Sephardic Jews	0.33	North America USA CEU	East Africa Ethiopia Ethiopians	0.39	8.2	1.67-20.20	0.08	East Africa Kenya Turkana	Middle East Turkey Sephardic Jews	36.51	25.18-51.18	0.15	West Africa Nigeria Yoruba	Middle East Turkey Sephardic Jews
14	North Africa Egypt Egyptian	1	1D	1D	0.01	0.97	1.00	1.00	22.52	16.76-28.29	0.16	West Africa Nigeria Yoruba	Middle East Turkey Sephardic Jews	0.34	North America USA CEU	East Africa Ethiopia Ethiopians	0.30	8.32		0.07	East Africa Kenya Turkana	Middle East Turkey Sephardic Jews	36.98		0.15	West Africa Nigeria Yoruba	Middle East Turkey Sephardic Jews
15	Caucasus Armenia Armenian	0	MW	MW	0.01	0.34	0.96	1.00	78.11	29.54-170.05	0.32	Central Asia Afghanistan	Middle East Lebanon Lebanese	0.26	Middle East Lebanon Lebanese	Middle East Lebanon Lebanese	0.04	1.00		0.45	Middle East Lebanon Lebanese	Middle East Lebanon Lebanese	80.91		0.4	Central Asia Afghanistan	Middle East Lebanon Lebanese
15	Caucasus Armenia Armenian	1	MW	MW	0.01	0.18	0.95	1.00	80.88	21.08-204.12	0.31	Central Asia Afghanistan	Middle East Lebanon Lebanese	0.31	Middle East Lebanon Lebanese	Middle East Lebanon Lebanese	0.04	10.84		0.48	Middle East Lebanon Lebanese	Middle East Lebanon Lebanese	94.56		0.34	South Asia India Brahmin	Middle East Lebanon Lebanese
15	Caucasus Azerbaijan Azerbaijani Jews	0	1D	1D	0.01	0.29	0.99	1.00	60.08	8.44-132.53	0.45	North Africa Egypt Egyptian	Central Asia Afghanistan	0.31	Middle East Lebanon Lebanese	Middle East Lebanon Lebanese	0.05	1.00		0.43	Middle East Lebanon Lebanese	Middle East Lebanon Lebanese	49.45		0.23	North Africa Egypt Egyptian	Middle East Lebanon Lebanese
15	Caucasus Azerbaijan Azerbaijani Jews	1	1D	1D	0.01	0.12	0.98	0.99	105.42	47.82-170.05	0.39	North Africa Egypt Egyptian	South Asia Pakistan Pathan	0.48	Middle East Lebanon Lebanese	Middle East Syria Syrians	0.05	1.00		0.46	Middle East Lebanon Lebanese	Middle East Lebanon Lebanese	77.01		0.26	North Africa Egypt Egyptian	Middle East Lebanon Lebanese
15	Caucasus Georgia Georgian	0	MW	MW	0.01	0.31	0.96	1.00	63.74	1.47-121.31	0.32	Central Asia Afghanistan	Middle East Lebanon Lebanese	0.32	Middle East Lebanon Lebanese	Middle East Lebanon Lebanese	0.05	1.00		0.40	Middle East Lebanon Lebanese	Middle East Lebanon Lebanese	71.76		0.33	Central Asia Afghanistan	Middle East Lebanon Lebanese
15	Caucasus Georgia Georgian	1	MW	MW	0.01	0.16	0.93	0.99	94.85	7.20-238.74	0.25	Central Asia Afghanistan	Middle East Lebanon Lebanese	0.21	Middle East Lebanon Lebanese	Middle East Lebanon Lebanese	0.03	1.00		0.44	Middle East Lebanon Lebanese	Middle East Lebanon Lebanese	79.14		0.24	Central Asia Afghanistan	Middle East Lebanon Lebanese
15	Caucasus Georgia Georgian Jews	0	MW	MW	0.01	0.15	0.88	1.00	53.10	2.93-106.70	0.22	Middle East Yemen Yemenite Jews	Middle East Lebanon Lebanese	0.35	South Asia Pakistan Pathan	Middle East Lebanon Lebanese	0.09	1.01		0.19	Middle East Yemen Yemenite Jews	Middle East Lebanon Lebanese	63.39		0.3	South Asia Pakistan Pathan	Middle East Lebanon Lebanese
15	Caucasus Georgia Georgian Jews	1	U	U	0.01	0.05	0.89	0.98	65.99	1.41-206.50	0.48	Middle East Yemen Yemenite Jews	Middle East Lebanon Lebanese	0.25	North Africa Egypt Egyptian	Middle East Lebanon Lebanese	0.04	1.94		0.34	Middle East Yemen Yemenite Jews	Middle East Lebanon Lebanese	12.45		0.21	South Asia Pakistan Pathan	Middle East Lebanon Lebanese
15	Caucasus Georgia Georgians	0	MW	MW	0.01	0.53	0.91	1.00	38.40	12.16-86.56	0.18	Central Asia Uzbekstan Uzbek	Middle East Lebanon Lebanese	0.29	Middle East Lebanon Lebanese	Middle East Lebanon Lebanese	0.04	17.44		0.13	Central Asia Uzbekstan Uzbek	Middle East Lebanon Lebanese	89.67		0.25	Middle East Jordania Jordanian	Central Asia Afghanistan
15	Caucasus Georgia Georgians	1	MW	MW	0.01	0.29	0.92	0.99	60.08	26.61-161.49	0.32	Central Asia Afghanistan	Middle East Lebanon Lebanese	0.28	Middle East Lebanon Lebanese	Middle East Lebanon Lebanese	0.04	7.49		0.38	Middle East Lebanon Lebanese	Middle East Lebanon Lebanese	33.64		0.39	Central Asia Afghanistan	Middle East Lebanon Lebanese
15	Caucasus Russia Lezgin	0	MW	MW	0.01	0.60	0.81	0.99	36.70	9.33-167.89	0.18	Central Asia Afghanistan	Middle East Lebanon Lebanese	0.37	Middle East Lebanon Lebanese	South Asia Pakistan Pathan	0.07	1.00		0.29	South Asia India Brahmin	Middle East Lebanon Lebanese	25.98		0.27	Central Asia Afghanistan	Middle East Lebanon Lebanese
15	Caucasus Russia Lezgin	1	MW	MW	0.01	0.35	0.87	1.00	33.42	12.42-185.56	0.09	East Asia China Uygur	Middle East Lebanon Lebanese	0.42	South Asia Pakistan Pathan	Middle East Lebanon Lebanese	0.03	16.7		0.17	Central Asia Afghanistan	Middle East Lebanon Lebanese	101.31		0.41	Central Asia Afghanistan	Middle East Lebanon Lebanese
15	Central Asia Iran Iranian	0	MW	MW	0.01	0.96	0.88	0.99	19.57	12.27-24.35	0.13	North Africa Egypt Egyptian	Middle East Lebanon Lebanese	0.28	Central Asia Afghanistan	Middle East Lebanon Lebanese	0.30	10.78		0.11	North Africa Egypt Egyptian	Middle East Lebanon Lebanese	57.96		0.27	North Africa Egypt Egyptian	South Asia Pakistan Pathan
15	Central Asia Iran Iranian	1	MW	MW	0.01	0.94	0.89	0.99	18.95	11.63-24.95	0.13	North Africa Egypt Egyptian	Middle East Lebanon Lebanese	0.28	Central Asia Afghanistan	Middle East Lebanon Lebanese	0.21	10.53		0.11	North Africa Egypt Egyptian	Middle East Lebanon Lebanese	59.4		0.28	North Africa Egypt Egyptian	Central Asia Afghanistan
15	Central Asia Iran Iranian Jew	0	MW	MW	0.01	0.18	0.96	0.99	79.77	48.14-123.38	0.27	North Africa Egypt Egyptian	Middle East Lebanon Lebanese	0.31	Middle East Lebanon Lebanese	Middle East Lebanon Lebanese	0.03	19.34		0.50	Middle East Lebanon Lebanese	Middle East Lebanon Lebanese	69.67		0.35	North Africa Egypt Egyptian	South Asia Pakistan Pathan
15	Central Asia Iran Iranian Jew	1	U	U	0.01	0.05	0.89	0.94	75.37	4.21-101.00	0.47	North Africa Egypt Egyptian	South Asia Pakistan Pathan	0.39	Middle East Lebanon Lebanese	Middle East Lebanon Lebanese	0.04	1.00		0.45	Middle East Lebanon Lebanese	Middle East Lebanon Lebanese	78.78		0.34	Middle East Yemen Yemenite Jews	South Asia Pakistan Pathan
15	Middle East Iraq Iraqi Jews	0	1D	1D	0.01	0.36	0.99	1.00	40.36	9.91-74.56	0.36	North Africa Egypt Egyptian	Middle East Lebanon Lebanese	0.43	Middle East Lebanon Lebanese	Middle East Lebanon Lebanese	0.08	1.00		0.46	Middle East Syria Syrians	Middle East Lebanon Lebanese	51.73		0.28	North Africa Egypt Egyptian	Middle East Lebanon Lebanese
15	Middle East Iraq Iraqi Jews	1	1D	1D	0.01	0.20	0.99	1.00	36.28	1.87-77.02	0.48	North Africa Egypt Egyptian	Middle East Lebanon Lebanese	0.32	Middle East Lebanon Lebanese	Middle East Lebanon Lebanese	0.04	1.00		0.46	Middle East Syria Syrians	Middle East Lebanon Lebanese	51.18		0.38	North Africa Egypt Egyptian	Middle East Lebanon Lebanese
15	Middle East Israel Druze	0	1D	1D	0.01	0.78	0.98	1.00	46.95	36.49-63.74	0.36	North Africa Egypt Egyptian	Middle East Lebanon Lebanese	0.32	Middle East Lebanon Lebanese	Middle East Lebanon Lebanese	0.07	18.49		0.40	North Africa Egypt Egyptian	Middle East Lebanon Lebanese	74.31		0.22	North Africa Egypt Egyptian	Middle East Lebanon Lebanese
15	Middle East Israel Druze	1	1D	1D	0.01	0.68	0.98	1.00	45.92	34.43-60.44	0.37	North Africa Egypt Egyptian	Middle East Lebanon Lebanese	0.29	Middle East Lebanon Lebanese	Middle East Lebanon Lebanese	0.08	9.35		0.24	North Africa Egypt Egyptian	Middle East Lebanon Lebanese	63.22		0.36	North Africa Egypt Egyptian	Middle East Lebanon Lebanese
15	Middle East Turkey Sephardic Jews	0	1D	1D	0.01	0.47	0.98	1.00	52.93	28.24-73.36	0.34	North Africa Egypt Egyptian	Middle East Lebanon Lebanese	0.39	Middle East Lebanon Lebanese	Middle East Lebanon Lebanese	0.08	6.17		0.22	Middle East Lebanon Lebanese	Middle East Lebanon Lebanese	64.11		0.16	North Africa Egypt Egyptian	Middle East Lebanon Lebanese
15	Middle East Turkey Sephardic Jews	1	U	U	0.01	0.19	0.95	0.98	36.28	8.01-108.64	0.31	Central Asia Afghanistan	Middle East Lebanon Lebanese	0.37	Middle East Lebanon Lebanese	Middle East Lebanon Lebanese	0.04	7.28		0.32	Middle East Lebanon Lebanese	Middle East Lebanon Lebanese	81.29		0.49	North Africa Egypt Egyptian	South Asia Pakistan Pathan

15	Middle East Turkey Turks	0	1D	1D	0.01	0.95	0.99	1.00	24.87	20.58-30.01	0.12	East Asia China Mongolian	Middle East Lebanon Lebanese	0.33	South Asia Pakistan Pathan	Middle East Lebanon Lebanese	0.14	1.00		0.29	Middle East Lebanon Lebanese	Middle East Lebanon Lebanese	27.99		0.13	East Asia China Mongolian	Middle East Lebanon Lebanese
15	Middle East Turkey Turks	1	1D	1D	0.01	0.93	0.99	1.00	24.35	20.40-29.54	0.13	East Asia China Mongolian	Middle East Lebanon Lebanese	0.34	South Asia Pakistan Pathan	Middle East Lebanon Lebanese	0.09	12.76		0.09	East Asia China Uygur	Middle East Lebanon Lebanese	36.98		0.12	East Asia China Mongolian	Middle East Lebanon Lebanese
15	North America USA CEU	0	MW	MW	0.01	0.32	0.97	0.99	68.66	44.05-161.49	0.34	South Asia Pakistan Pathan	Middle East Lebanon Lebanese	0.36	Middle East Lebanon Lebanese	Middle East Lebanon Lebanese	0.04	13.27		0.41	Middle East Lebanon Lebanese	Middle East Lebanon Lebanese	43.05		0.29	South Asia Pakistan Pathan	Middle East Lebanon Lebanese
15	North America USA CEU	1	MW	MW	0.01	0.21	0.97	1.00	88.31	31.45-216.17	0.38	South Asia Pakistan Pathan	Middle East Lebanon Lebanese	0.49	Middle East Lebanon Lebanese	Middle East Lebanon Lebanese	0.03	13.98		0.49	Middle East Lebanon Lebanese	Middle East Lebanon Lebanese	42.96		0.3	South Asia Pakistan Pathan	Middle East Lebanon Lebanese
16	East Africa Malagasy Mikea	0	1D	1D	0.01	0.99	1.00	1.00	27.46	24.93-30.64	0.33	Island Southeast Asia Indonesia Banjar	South Africa South African Bantu Soweto	0.24	East Africa Kenya BantuKenya	South Africa South African Bantu Soweto	0.16	18.57		0.39	Island Southeast Asia Indonesia Banjar	South Africa South African Bantu Soweto	42.00		0.3	Island Southeast Asia Indonesia Banjar	South Africa South African Bantu Soweto
16	East Africa Malagasy Mikea	1	1D	1D	0.01	0.99	1.00	1.00	27.89	23.23-30.49	0.33	Island Southeast Asia Indonesia Banjar	South Africa South African Bantu Soweto	0.42	South Africa South African Bantu Soweto	East Africa Kenya BantuKenya	0.22	15.69		0.47	Island Southeast Asia Indonesia Banjar	South Africa South African Bantu Soweto	38.66		0.33	Island Southeast Asia Indonesia Banjar	South Africa South African Bantu Soweto
16	East Africa Malagasy Temoro	0	1D	1D	0.01	1.00	1.00	1.00	29.95	27.34-32.96	0.35	Island Southeast Asia Indonesia Banjar	South Africa South African Bantu Soweto	0.20	East Africa Kenya BantuKenya	South Africa South African Bantu Soweto	0.26	4.24		0.28	East Africa Kenya BantuKenya	South Africa South African Bantu Soweto	30.87		0.36	Island Southeast Asia Indonesia Banjar	South Africa South African Bantu Soweto
16	East Africa Malagasy Temoro	1	1D	1D	0.01	0.99	1.00	1.00	30.63	28.48-33.57	0.35	Island Southeast Asia Indonesia Banjar	South Africa South African Bantu Soweto	0.24	East Africa Kenya BantuKenya	South Africa South African Bantu Soweto	0.15	27.56		0.39	Island Southeast Asia Indonesia Banjar	South Africa South African Bantu Soweto	43.06		0.48	South Africa South African Bantu Soweto	Island Southeast Asia Indonesia Banjar
16	East Africa Malagasy Vezo	0	1D	1D	0.01	0.99	1.00	1.00	25.61	22.30-28.19	0.36	Island Southeast Asia Indonesia Banjar	South Africa South African Bantu Soweto	0.22	East Africa Kenya BantuKenya	South Africa South African Bantu Soweto	0.18	22.8		0.36	Island Southeast Asia Indonesia Banjar	South Africa South African Bantu Soweto	58.51		0.21	Island Southeast Asia Indonesia Banjar	South Africa South African Bantu Soweto
16	East Africa Malagasy Vezo	1	1D	1D	0.01	0.99	1.00	1.00	25.84	23.26-28.72	0.36	Island Southeast Asia Indonesia Banjar	South Africa South African Bantu Soweto	0.25	East Africa Kenya BantuKenya	South Africa South African Bantu Soweto	0.15	22.98		0.36	Island Southeast Asia Indonesia Banjar	South Africa South African Bantu Soweto	59.19		0.3	Island Southeast Asia Indonesia Banjar	South Africa South African Bantu Soweto
16	South Africa South Africa ColouredColesberg	0	2D	2D	0.01	1.00	0.93	1.00	6.17	4.50-6.85	0.30	Caucasus Russia Lezgin	South Africa South Africa Karretjie	0.43	South Africa South African Bantu Soweto	South Africa South Africa Karretjie	0.41	5.76	1.33-16.09	0.28	North America USA CEU	South Africa South Africa Karretjie	9.03	1.52-27.36	0.48	South Africa South Africa Karretjie	South Africa South African Bantu Soweto
16	South Africa South Africa ColouredColesberg	1	MW	MW	0.01	1.00	0.94	1.00	6.26	3.99-7.29	0.30	Caucasus Russia Lezgin	South Africa South Africa Karretjie	0.43	South Africa South African Bantu Soweto	South Africa South Africa Karretjie	0.32	5.81		0.28	North America USA CEU	South Africa South Africa Karretjie	10.41		0.49	South Africa South Africa Karretjie	South Africa South African Bantu Soweto
16	South Africa South Africa ColouredWellington	0	2D	2D	0.01	1.00	0.98	0.99	5.04	4.45-5.52	0.45	South Africa South African Bantu Soweto	South Asia India West Bengal Brahmin	0.45	South Africa Namibia Nama	South Africa South African Bantu Soweto	0.64	4.17	2.67-21.84	0.46	South Africa South African Bantu Soweto	South Asia India Brahmin	13.92	1.59-27.59	0.41	Mainland Southeast Asia Malaysia Malay	South Africa South Africa Karretjie
16	South Africa South Africa ColouredWellington	1	2D	2D	0.01	1.00	0.98	0.99	5.11	4.44-5.92	0.45	South Africa South African Bantu Soweto	South Asia India West Bengal Brahmin	0.44	South Africa Namibia Nama	South Africa South African Bantu Soweto	0.55	4.31	2.00-15.98	0.45	South Africa South African Bantu Soweto	South Asia India Brahmin	16.67	3.67-36.67	0.46	South Asia India Bengali	South Africa South Africa SEBantu
17	Central Africa CAR Biaka Pygmy	0	1D	1D	0.01	0.81	1.00	1.00	27.83	20.72-32.82	0.49	Central Africa Gabon Nzebi	Central Africa Gabon Nzebi	0.49	Central Africa Gabon Nzebi	Central Africa Gabon Nzebi	0.06	1.00		0.48	West Africa Nigeria Yoruba	Central Africa Gabon Nzebi	29.98		0.45	Central Africa Gabon Nzebi	Central Africa Gabon Nzebi
17	Central Africa CAR Biaka Pygmy	1	1D	1D	0.01	0.75	1.00	1.00	51.75	42.38-62.91	0.40	South Africa South Africa ColouredWellington	Central Africa Gabon Nzebi	0.35	West Africa Nigeria Yoruba	Central Africa Gabon Nzebi	0.09	5.47		0.48	Central Africa Gabon Nzebi	Central Africa Gabon Nzebi	44.25		0.39	West Africa Nigeria Yoruba	Central Africa Gabon Nzebi
17	Central Africa DR Congo Mbuti Pygmy	0	MW	MW	0.01	0.71	0.80	0.99	22.76	15.45-29.73	0.35	East Africa Kenya Luhya	East Africa Ethiopia Anuak	0.36	West Africa Nigeria Yoruba	East Africa Kenya Luhya	0.13	1.25		0.33	West Africa Nigeria Yoruba	East Africa Kenya Luhya	28.14		0.32	East Africa Kenya Luhya	East Africa Ethiopia Anuak
17	Central Africa DR Congo Mbuti Pygmy	1	MW	MW	0.01	0.64	0.88	1.00	31.25	21.49-39.92	0.31	East Africa Kenya Luhya	East Africa Ethiopia Anuak	0.34	East Africa Kenya BantuKenya	East Africa Kenya Luhya	0.02	1.17		0.35	East Africa Kenya BantuKenya	East Africa Kenya Luhya	27.47		0.3	West Africa Nigeria Yoruba	East Africa Ethiopia Anuak
17	Central Africa Gabon Baka	0	1D	1D	0.01	0.74	1.00	1.00	14.11	8.77-19.46	0.48	Central Africa Gabon Nzebi	Central Africa Gabon Nzebi	0.30	West Africa Nigeria Yoruba	Central Africa Gabon Nzebi	0.17	7.98		0.49	Central Africa Gabon Nzebi	Central Africa Gabon Nzebi	33.84		0.47	Central Africa Gabon Nzebi	Central Africa Gabon Nzebi
17	Central Africa Gabon Baka	1	1D	1D	0.01	0.60	1.00	1.00	33.12	25.21-45.29	0.46	Central Africa Gabon Nzebi	Central Africa Gabon Nzebi	0.33	West Africa Nigeria Yoruba	Central Africa Gabon Nzebi	0.04	7.09		0.36	Central Africa Gabon Nzebi	Central Africa Gabon Nzebi	47.9		0.43	Central Africa Gabon Nzebi	Central Africa Gabon Nzebi
17	Central Africa Gabon Bongo	0	1D	1D	0.01	0.85	1.00	1.00	14.47	10.72-18.26	0.30	Central Africa Gabon Nzebi	Central Africa Gabon Nzebi	0.35	Central Africa Gabon Nzebi	Central Africa Gabon Nzebi	0.16	6.74		0.39	Central Africa Gabon Nzebi	Central Africa Gabon Nzebi	29.44		0.18	South Africa South Africa ColouredColesberg	Central Africa Gabon Nzebi
17	Central Africa Gabon Bongo	1	1D	1D	0.01	0.83	1.00	1.00	30.77	23.63-39.15	0.23	South Africa South Africa ColouredWellington	Central Africa Gabon Nzebi	0.35	Central Africa Gabon Nzebi	Central Africa Gabon Nzebi	0.14	13.23		0.46	Central Africa Gabon Nzebi	Central Africa Gabon Nzebi	68.25		0.18	South Africa South Africa ColouredColesberg	Central Africa Gabon Nzebi
17	East Africa Tanzania Sandawe	0	1D	1D	0.01	0.96	1.00	1.00	25.73	21.33-29.45	0.43	East Africa Kenya BantuKenya	East Africa Ethiopia Wolayta	0.47	East Africa Kenya BantuKenya	East Africa Kenya Luhya	0.25	2.32		0.40	East Africa Kenya BantuKenya	East Africa Kenya Turkana	27.44		0.43	East Africa Kenya BantuKenya	East Africa Ethiopia Wolayta
17	East Africa Tanzania	1	1D	1D	0.01	0.95	1.00	1.00	29.42	25.32-33.72	0.42	East Africa Kenya	East Africa Ethiopia	0.48	East Africa Kenya BantuKenya	East Africa Kenya Luhya	0.17	3.69		0.37	East Africa Kenya BantuKenya	East Africa Kenya Turkana	34.81		0.44	East Africa Kenya	East Africa Ethiopia

	Sandawe											BantuKenya	Wolayta													BantuKenya	Wolayta
17	East Africa Uganda Batwa	0	1D	1D	0.01	0.90	0.99	1.00	18.70	14.61-22.76	0.38	East Africa Kenya Turkana	East Africa Kenya BantuKenya	0.22	East Africa Uganda Bakiga	East Africa Kenya BantuKenya	0.23	1.00		0.30	East Africa Kenya BantuKenya	East Africa Kenya BantuKenya	26.74		0.39	East Africa Kenya Turkana	East Africa Kenya BantuKenya
17	East Africa Uganda Batwa	1	1D	1D	0.01	0.90	0.99	1.00	26.26	22.58-30.63	0.37	East Africa Kenya Turkana	East Africa Kenya BantuKenya	0.22	East Africa Uganda Bakiga	East Africa Kenya BantuKenya	0.17	1.00		0.35	East Africa Kenya BantuKenya	East Africa Kenya BantuKenya	35.16		0.36	East Africa Kenya Turkana	East Africa Kenya BantuKenya
18	Central Africa Gabon Nzebi	0	U	MW	0.01	0.59	0.92	0.99	2.66	1.78-6.28	0.29	Central Africa Gabon Bongo	South Africa South Africa BantuSA	0.13	South Africa South Africa BantuSA	South Africa South Africa BantuSA	0.16	3.1		0.35	South Africa South Africa BantuSA	Central Africa Gabon Bongo	30.57		0.32	Central Africa Gabon Bongo	South Africa South Africa BantuSA
18	Central Africa Gabon Nzebi	1	U	MW	0.01	0.27	0.95	0.99	91.25	10.75-153.15	0.26	Central Africa Gabon Bongo	South Africa South Africa BantuSA	0.26	South Africa South Africa BantuSA	South Africa South Africa BantuSA	0.09	7.06		0.24	Central Africa Gabon Bongo	South Africa South Africa BantuSA	129.84		0.38	Central Africa Gabon Bongo	South Africa South Africa BantuSA
18	East Africa Kenya BantuKenya	0	1D	1D	0.01	0.91	1.00	1.00	18.12	14.28-24.31	0.38	East Africa Ethiopia Anuak	South Africa South Africa BantuSA	0.39	South Africa South Africa BantuSA	South Africa South Africa BantuSA	0.10	3.15		0.46	South Africa South Africa BantuSA	South Africa South Africa BantuSA	20.1		0.32	East Africa Ethiopia Anuak	South Africa South Africa BantuSA
18	East Africa Kenya BantuKenya	1	1D	1D	0.01	0.89	1.00	1.00	21.49	15.69-25.95	0.37	East Africa Ethiopia Anuak	South Africa South Africa BantuSA	0.42	South Africa South Africa BantuSA	South Africa South Africa BantuSA	0.09	13.96		0.41	East Africa Ethiopia Anuak	South Africa South Africa BantuSA	44.96		0.33	East Africa Kenya Turkana	South Africa South Africa BantuSA
18	East Africa Kenya Luhya	0	1D	1D	0.01	0.97	1.00	1.00	18.81	15.55-21.16	0.38	East Africa Ethiopia Anuak	South Africa South Africa BantuSA	0.47	South Africa South Africa BantuSA	South Africa South Africa BantuSA	0.22	2.73		0.36	East Africa Kenya Turkana	South Africa South Africa BantuSA	24.78		0.37	East Africa Ethiopia Anuak	South Africa South Africa BantuSA
18	East Africa Kenya Luhya	1	1D	1D	0.01	0.97	1.00	1.00	21.85	18.48-26.26	0.39	East Africa Ethiopia Anuak	South Africa South Africa BantuSA	0.47	South Africa South Africa BantuSA	South Africa South Africa BantuSA	0.22	15.42		0.35	East Africa Ethiopia Anuak	South Africa South Africa BantuSA	60.71		0.42	East Africa Kenya Turkana	South Africa South Africa BantuSA
18	East Africa Uganda Bakiga	0	MW	MW	0.01	0.95	0.97	1.00	18.77	15.23-21.85	0.24	East Africa Kenya Turkana	South Africa South Africa BantuSA	0.47	South Africa South Africa BantuSA	South Africa South Africa BantuSA	0.32	1.00		0.37	East Africa Kenya Turkana	South Africa South Africa BantuSA	27.25		0.24	East Africa Kenya Turkana	South Africa South Africa BantuSA
18	East Africa Uganda Bakiga	1	MW	MW	0.01	0.95	0.97	1.00	22.43	18.15-26.68	0.25	East Africa Kenya Turkana	South Africa South Africa BantuSA	0.50	South Africa South Africa BantuSA	South Africa South Africa BantuSA	0.33	1.72		0.31	East Africa Kenya Turkana	South Africa South Africa BantuSA	32.75		0.25	East Africa Kenya Turkana	South Africa South Africa BantuSA
18	South Africa South Africa SWBantu	0	MW	MW	0.01	0.91	0.92	1.00	1.00	1.00-2.68	0.28	South Africa Namibia Nama	South Africa South Africa BantuSA	0.34	South Africa Namibia Nama	South Africa South Africa BantuSA	0.27	1.00		0.26	South Africa Namibia Nama	South Africa South Africa BantuSA	130.26		0.35	South Africa Namibia Nama	South Africa South Africa BantuSA
18	South Africa South Africa SWBantu	1	MW	MW	0.01	0.86	0.90	1.00	1.00	1.00-2.67	0.26	South Africa Namibia Nama	South Africa South Africa BantuSA	0.33	South Africa Namibia Nama	South Africa South Africa BantuSA	0.25	1.00		0.24	South Africa Namibia Nama	South Africa South Africa BantuSA	49.34		0.31	South Africa Namibia Nama	South Africa South Africa BantuSA
18	West Africa Nigeria Yoruba	0	U	U	0.01	0.84	0.96	0.98	5.68	2.96-8.23	0.24	South Africa South Africa BantuSA	South Africa South Africa BantuSA	0.50	South Africa South Africa BantuSA	South Africa South Africa BantuSA	0.13	1.94		0.31	South Africa South Africa BantuSA	South Africa South Africa BantuSA	48.22		0.48	South Africa South Africa BantuSA	South Africa South Africa BantuSA
18	West Africa Nigeria Yoruba	1	U	U	0.01	0.19	0.93	0.96	167.89	17.05-266.12	0.49	East Africa Kenya Turkana	South Africa South Africa BantuSA	0.47	South Africa South Africa BantuSA	South Africa South Africa BantuSA	0.03	7.79		0.42	South Africa South Africa BantuSA	South Africa South Africa BantuSA	183.48		0.48	East Africa Kenya Turkana	South Africa South Africa BantuSA
19	South Africa South Africa BantuSA	0	1D	1D	0.01	0.84	1.00	1.00	31.64	25.69-45.64	0.45	South Africa Namibia Nama	Central Africa Gabon Nzebi	0.46	South Africa South Africa ColouredWellington	South Africa South Africa ColouredWellington	0.09	6.1		0.14	Central Africa Gabon Nzebi	South Africa South Africa ColouredWellington	41.73		0.43	South Africa Namibia Nama	Central Africa Gabon Nzebi
19	South Africa South Africa BantuSA	1	1D	1D	0.01	0.73	1.00	1.00	31.45	21.49-42.45	0.46	South Africa Namibia Nama	Central Africa Gabon Nzebi	0.36	West Africa Nigeria Yoruba	South Africa South Africa ColouredWellington	0.06	9.87		0.16	Central Africa Gabon Nzebi	South Africa South Africa ColouredWellington	49.73		0.44	South Africa Namibia Nama	Central Africa Gabon Nzebi
19	South Africa South Africa SEBantu	0	1D	1D	0.01	0.96	1.00	1.00	28.06	23.63-32.62	0.35	South Africa South Africa Karretjie	East Africa Kenya BantuKenya	0.48	South Africa South Africa ColouredColesberg	South Africa South Africa ColouredWellington	0.22	20.87		0.37	South Africa South Africa ColouredColesberg	East Africa Kenya BantuKenya	99.41		0.36	South Africa South Africa Karretjie	Central Africa Gabon Nzebi
19	South Africa South Africa SEBantu	1	1D	1D	0.01	0.95	1.00	1.00	27.99	24.08-32.94	0.35	South Africa South Africa Karretjie	East Africa Kenya BantuKenya	0.45	South Africa South Africa ColouredColesberg	South Africa South Africa ColouredWellington	0.16	21.07		0.40	South Africa South Africa ColouredColesberg	East Africa Kenya BantuKenya	101.57		0.37	South Africa South Africa Karretjie	Central Africa Gabon Nzebi
19	South Africa South African Bantu Soweto	0	1D	1D	0.01	0.96	1.00	1.00	28.91	23.87-33.42	0.32	South Africa South Africa Karretjie	East Africa Kenya BantuKenya	0.39	Central Africa Gabon Nzebi	South Africa South Africa ColouredWellington	0.29	2.48		0.31	South Africa South Africa ColouredColesberg	East Africa Kenya BantuKenya	36.7		0.33	South Africa South Africa Karretjie	Central Africa Gabon Nzebi
19	South Africa South African Bantu Soweto	1	1D	1D	0.01	0.96	1.00	1.00	29.75	25.78-34.27	0.32	South Africa South Africa Karretjie	East Africa Kenya BantuKenya	0.32	Central Africa Gabon Nzebi	South Africa South Africa ColouredWellington	0.16	17.41		0.29	South Africa South Africa ColouredColesberg	Central Africa Gabon Nzebi	57.81		0.35	South Africa South Africa Karretjie	East Africa Kenya BantuKenya
20	East Africa Tanzania SAN NB	0	1D	1D	0.01	0.49	1.00	1.00	25.73	9.10-41.92	0.49	South Africa South African Bantu Soweto	South Africa South African Bantu Soweto	0.23	South Africa South African Bantu Soweto	South Africa South African Bantu Soweto	0.09	1.00		0.33	South Africa South African Bantu Soweto	South Africa South African Bantu Soweto	42.75		0.31	South Africa South African Bantu Soweto	South Africa South African Bantu Soweto
20	East Africa Tanzania SAN NB	1	1D	1D	0.01	0.16	1.00	1.00	55.49	25.73-79.77	0.29	South Africa South African Bantu Soweto	South Africa South African Bantu Soweto	0.34	South Africa South African Bantu Soweto	South Africa South African Bantu Soweto	0.02	1.00		0.25	South Africa South African Bantu Soweto	South Africa South African Bantu Soweto	53.65		0.25	East Africa Kenya Turkana	South Africa South African Bantu Soweto
20	South Africa Botswana GuiGhanaKgal	0	1D	1D	0.01	0.86	1.00	1.00	9.48	6.59-13.43	0.26	South Africa South African Bantu Soweto	South Africa South African Bantu Soweto	0.08	South Africa South African Bantu Soweto	South Africa South Africa SEBantu	0.14	1.08		0.44	South Africa South African Bantu Soweto	South Africa South Africa SEBantu	21.58		0.13	South Africa South African Bantu Soweto	South Africa South Africa SEBantu
20	South Africa Botswana GuiGhanaKgal	1	1D	1D	0.01	0.74	1.00	1.00	21.02	11.70-32.23	0.12	South Africa South African Bantu Soweto	South Africa South Africa SEBantu	0.15	South Africa South African Bantu Soweto	South Africa South Africa SEBantu	0.17	1.00		0.40	South Africa South Africa SEBantu	South Africa South African Bantu Soweto	35.83		0.12	East Africa Kenya Turkana	South Africa South Africa SEBantu
20	South Africa Namibia Juhoansi	0	U	1D	0.01	0.73	1.00	1.00	23.51	14.61-36.70	0.49	South Africa South African Bantu Soweto	South Africa South African Bantu Soweto	0.09	South Africa South African Bantu Soweto	South Africa South African Bantu Soweto	0.21	3.11		0.44	South Africa South African Bantu Soweto	South Africa South African Bantu Soweto	53.18		0.17	East Africa Kenya Turkana	South Africa South African Bantu Soweto

20	South Africa Namibia Juhoansi	1	U	1D	0.01	0.52	1.00	1.00	61.17	43.44-80.04	0.16	East Africa Kenya Turkana	South Africa South African Bantu Soweto	0.44	South Africa South African Bantu Soweto	South Africa South African Bantu Soweto	0.03	1.00		0.37	South Africa South African Bantu Soweto	South Africa South African Bantu Soweto	59.78		0.18	East Africa Kenya Turkana	South Africa South African Bantu Soweto
20	South Africa Namibia Nama	0	2D	2D	0.01	0.98	1.00	1.00	6.91	4.09-9.73	0.43	South Africa South African Bantu Soweto	South Africa South African Bantu Soweto	0.17	South Africa South Africa BantuSA	South Africa South Africa SEBantu	0.70	2.87	1.67-30.20	0.39	South Africa South African Bantu Soweto	South Africa South African Soweto	38.28	28.61-60.28	0.22	South Africa South Africa BantuSA	South Africa South African Bantu Soweto
20	South Africa Namibia Nama	1	2D	2D	0.01	0.98	1.00	1.00	6.04	3.25-8.28	0.44	South Africa South African Bantu Soweto	South Africa South African Bantu Soweto	0.16	South Africa South Africa BantuSA	South Africa South Africa SEBantu	0.69	2.49	1.13-26.16	0.38	South Africa South Africa SEBantu	South Africa South African Soweto	47.96	38.29-67.63	0.22	East Africa Kenya Turkana	South Africa South African Bantu Soweto
20	South Africa South Africa/Namibia Khwe	0	U	MW	0.01	0.88	0.81	1.00	13.41	8.71-16.37	0.27	South Africa South African Bantu Soweto	South Africa South African Bantu Soweto	0.44	Central Africa Gabon Nzebi	South Africa South African Bantu Soweto	0.26	5.25		0.50	South Africa South African Bantu Soweto	South Africa South African Bantu Soweto	27.19		0.31	Central Africa Gabon Nzebi	South Africa South African Bantu Soweto
20	South Africa South Africa/Namibia Khwe	1	U	MW	0.01	0.81	0.91	1.00	27.11	21.13-34.12	0.41	South Africa South African Bantu Soweto	Central Africa Gabon Nzebi	0.49	Central Africa Gabon Nzebi	South Africa South African Bantu Soweto	0.16	8.56		0.41	Central Africa Gabon Nzebi	South Africa South African Bantu Soweto	45.29		0.27	East Africa Kenya BantuKenya	South Africa South African Bantu Soweto
20	South Africa South Africa/Namibia Xun	0	1D	1D	0.01	0.76	0.99	1.00	16.16	8.60-35.66	0.50	South Africa South African Bantu Soweto	South Africa South African Bantu Soweto	0.09	Central Africa Gabon Nzebi	South Africa South African Bantu Soweto	0.35	2.17		0.29	South Africa South African Bantu Soweto	South Africa South African Bantu Soweto	64.94		0.17	East Africa Kenya Turkana	South Africa South African Bantu Soweto
20	South Africa South Africa/Namibia Xun	1	1D	1D	0.01	0.62	0.98	1.00	58.47	30.30-90.36	0.25	East Africa Kenya Turkana	South Africa South African Bantu Soweto	0.13	Central Africa Gabon Nzebi	South Africa South African Bantu Soweto	0.11	5.55		0.25	South Africa South African Bantu Soweto	South Africa South African Bantu Soweto	87.85		0.19	East Africa Ethiopia Wolayta	South Africa South African Bantu Soweto
20	South Africa South Africa Karretjie	0	1D	1D	0.01	1.00	1.00	1.00	5.16	3.97-6.40	0.25	North America USA CEU	South Africa South Africa SEBantu	0.08	Central Africa DR Congo Mbuti Pygmy	South Africa South Africa SEBantu	0.12	4.58		0.26	South Africa South Africa SEBantu	South Africa South Africa SEBantu	21.19		0.03	East Africa Ethiopia Ari Cultivator	South Africa South Africa SEBantu
20	South Africa South Africa Karretjie	1	1D	1D	0.01	1.00	1.00	1.00	5.13	3.81-6.08	0.26	South Africa South Africa SEBantu	South Africa South Africa SEBantu	0.09	Central Africa DR Congo Mbuti Pygmy	South Africa South Africa SEBantu	0.10	4.68		0.26	South Africa South Africa SEBantu	South Africa South Africa SEBantu	58.87		0.06	East Africa Ethiopia Ari Cultivator	South Africa South Africa SEBantu
20	South Africa South Africa Khomani	0	2D	2D	0.01	1.00	1.00	1.00	6.00	4.90-7.91	0.22	North America USA CEU	South Africa South Africa SEBantu	0.16	East Africa Tanzania Sandawe	South Africa South Africa SEBantu	0.53	5.3	1.33-33.63	0.41	South Africa South Africa SEBantu	South Africa South Africa SEBantu	53.21	36.54-74.21	0.16	East Africa Ethiopia Wolayta	South Africa South Africa SEBantu
20	South Africa South Africa Khomani	1	2D	2D	0.01	1.00	1.00	1.00	6.08	4.79-7.29	0.26	North America USA CEU	South Africa South Africa SEBantu	0.16	East Africa Tanzania Sandawe	South Africa South Africa SEBantu	0.49	5.36	2.33-35.69	0.42	South Africa South Africa SEBantu	South Africa South Africa SEBantu	57.81	40.81-75.81	0.16	East Africa Ethiopia Wolayta	South Africa South Africa SEBantu
20	South Africa South Africa San	0	2D	2D	0.01	0.99	1.00	1.00	6.16	4.80-7.15	0.17	North America USA CEU	South Africa South Africa SEBantu	0.15	East Africa Tanzania Sandawe	South Africa South Africa SEBantu	0.44	5.29	1.46-16.62	0.20	North America USA CEU	South Africa South Africa SEBantu	65.55	58.55-86.22	0.12	East Africa Ethiopia Wolayta	South Africa South Africa SEBantu
20	South Africa South Africa San	1	2D	2D	0.01	0.99	1.00	1.00	6.15	4.49-7.91	0.19	North America USA CEU	South Africa South Africa SEBantu	0.15	East Africa Tanzania Sandawe	South Africa South Africa SEBantu	0.43	5.19	2.05-36.19	0.26	South Africa South Africa SEBantu	South Africa South Africa SEBantu	70.09	46.76-84.42	0.12	East Africa Ethiopia Wolayta	South Africa South Africa SEBantu
21	East Africa Ethiopia Afar	0	2D	2D	0.01	0.92	1.00	1.00	45.61	35.19-54.46	0.43	North Africa Egypt Egyptian	East Africa Kenya Turkana	0.42	East Africa Ethiopia Ari Cultivator	East Africa Kenya Turkana	0.47	4.92	1.78-26.59	0.38	North Africa Egypt Egyptian	East Africa Kenya Turkana	58.41	42.74-75.41	0.5	North Africa Egypt Egyptian	East Africa Kenya Turkana
21	East Africa Ethiopia Afar	1	1D	1D	0.01	0.86	1.00	1.00	49.03	37.23-61.35	0.43	North Africa Egypt Egyptian	East Africa Kenya Turkana	0.31	East Africa Ethiopia Ari Cultivator	East Africa Kenya Turkana	0.08	12.55		0.22	North Africa Egypt Egyptian	East Africa Kenya Turkana	75.35		0.48	East Africa Kenya Turkana	North Africa Egypt Egyptian
21	East Africa Ethiopia Amhara	0	2D	2D	0.01	0.94	0.99	1.00	53.60	43.90-65.14	0.50	North Africa Egypt Egyptian	East Africa Kenya Turkana	0.36	East Africa Ethiopia Ari Cultivator	East Africa Kenya Turkana	0.64	6.53	1.33-30.53	0.44	North Africa Egypt Egyptian	East Africa Kenya Turkana	68.57	47.57-80.57	0.47	East Africa Kenya Turkana	North Africa Egypt Egyptian
21	East Africa Ethiopia Amhara	1	1D	1D	0.01	0.92	0.99	1.00	60.62	54.12-71.68	0.49	North Africa Egypt Egyptian	East Africa Kenya Turkana	0.39	East Africa Ethiopia Ari Cultivator	East Africa Kenya Turkana	0.20	18.99		0.46	North Africa Egypt Egyptian	East Africa Kenya Turkana	85.66		0.42	East Africa Kenya Turkana	North Africa Egypt Egyptian
21	East Africa Ethiopia Ethiopian Jews	0	2D	2D	0.01	0.87	0.99	1.00	51.25	40.14-64.39	0.49	North Africa Egypt Egyptian	East Africa Kenya Turkana	0.30	East Africa Ethiopia Ari Cultivator	East Africa Kenya Turkana	0.47	4.2	2.43-15.20	0.27	East Africa Kenya Turkana	East Africa Kenya Turkana	67.25	55.92-90.58	0.49	East Africa Kenya Turkana	North Africa Egypt Egyptian
21	East Africa Ethiopia Ethiopian Jews	1	1D	1D	0.01	0.83	0.99	1.00	53.77	41.86-65.42	0.45	North Africa Egypt Egyptian	East Africa Kenya Turkana	0.35	East Africa Ethiopia Ari Cultivator	East Africa Kenya Turkana	0.23	4.33		0.23	East Africa Kenya Turkana	East Africa Kenya Turkana	69.83		0.5	North Africa Egypt Egyptian	East Africa Kenya Turkana
21	East Africa Ethiopia Ethiopians	0	1D	1D	0.01	0.85	0.99	1.00	53.94	33.22-70.21	0.44	East Africa Kenya Turkana	North Africa Egypt Egyptian	0.33	East Africa Ethiopia Ari Cultivator	East Africa Kenya Turkana	0.24	5.67		0.39	North Africa Egypt Egyptian	East Africa Kenya Turkana	77.92		0.4	East Africa Kenya Turkana	North Africa Egypt Egyptian
21	East Africa Ethiopia Ethiopians	1	1D	1D	0.01	0.68	0.99	1.00	54.80	36.91-73.96	0.42	East Africa Kenya Turkana	North Africa Egypt Egyptian	0.38	East Africa Ethiopia Ari Cultivator	East Africa Kenya Turkana	0.07	14.49		0.35	North Africa Egypt Egyptian	East Africa Kenya Turkana	87.65		0.34	East Africa Kenya Turkana	North Africa Egypt Egyptian
21	East Africa Ethiopia Oromo	0	2D	2D	0.01	0.91	0.92	1.00	35.92	27.87-43.90	0.44	North Africa Egypt Egyptian	East Africa Kenya Turkana	0.39	East Africa Ethiopia Ari Cultivator	East Africa Kenya Turkana	0.58	8.12	1.72-23.12	0.48	East Africa Kenya Turkana	East Africa Ethiopia Ari Cultivator	71.5	58.50-82.83	0.38	North Africa Egypt Egyptian	East Africa Kenya Turkana
21	East Africa Ethiopia Oromo	1	MW	MW	0.01	0.90	0.92	1.00	44.59	35.86-53.26	0.43	North Africa Egypt Egyptian	East Africa Kenya Turkana	0.44	East Africa Ethiopia Ari Cultivator	East Africa Kenya Turkana	0.30	15.98		0.49	East Africa Ethiopia Ari Cultivator	East Africa Kenya Turkana	98.21		0.46	North Africa Egypt Egyptian	East Africa Kenya Turkana
21	East Africa Ethiopia Somali	0	2D	2D	0.01	0.87	0.97	1.00	46.31	28.89-59.09	0.27	North Africa Egypt Egyptian	East Africa Kenya Turkana	0.14	East Africa Ethiopia Ari Cultivator	East Africa Kenya Turkana	0.50	7.88	2.67-29.88	0.09	North Africa Egypt Egyptian	East Africa Kenya Turkana	85.44	63.44-104.11	0.42	North Africa Egypt Egyptian	East Africa Kenya Turkana
21	East Africa Ethiopia Somali	1	1D	1D	0.01	0.85	0.98	1.00	57.59	43.75-75.80	0.30	North Africa Egypt Egyptian	East Africa Kenya Turkana	0.24	East Africa Ethiopia Ari Cultivator	East Africa Kenya Turkana	0.14	11.08		0.30	East Africa Kenya Turkana	East Africa Kenya Turkana	86.04		0.41	North Africa Egypt Egyptian	East Africa Kenya Turkana
21	East Africa Ethiopia Tygray	0	2D	2D	0.01	0.92	0.99	1.00	56.75	44.98-67.12	0.47	East Africa Kenya Turkana	North Africa Egypt Egyptian	0.35	East Africa Ethiopia Ari Cultivator	East Africa Kenya Turkana	0.60	9.56	1.72-38.56	0.47	East Africa Kenya Turkana	North Africa Egypt Egyptian	83.34	75.67-105.34	0.39	East Africa Kenya Turkana	North Africa Egypt Egyptian

21	East Africa Ethiopia Tygray	1	1D	1D	0.01	0.91	0.99	1.00	65.14	54.80-75.26	0.44	East Africa Kenya Turkana	North Africa Egypt Egyptian	0.43	East Africa Ethiopia Ari Cultivator	East Africa Kenya Turkana	0.16	12.47		0.41	North Africa Egypt Egyptian	East Africa Kenya Turkana	87.16		0.38	East Africa Kenya Turkana	North Africa Egypt Egyptian
21	East Africa Ethiopia Wolayta	0	1D	1D	0.01	0.88	0.98	1.00	42.76	32.43-53.60	0.42	East Africa Ethiopia Ari Cultivator	North Africa Egypt Egyptian	0.46	East Africa Kenya Turkana	East Africa Ethiopia Ari Cultivator	0.31	2.36		0.21	East Africa Ethiopia Ari Cultivator	East Africa Kenya Turkana	52.97		0.49	East Africa Ethiopia Ari Cultivator	North Africa Egypt Egyptian
21	East Africa Ethiopia Wolayta	1	MW	MW	0.01	0.80	0.97	1.00	48.14	37.23-63.65	0.46	East Africa Ethiopia Ari Cultivator	North Africa Egypt Egyptian	0.37	East Africa Kenya Turkana	East Africa Ethiopia Ari Cultivator	0.13	1.2		0.36	East Africa Ethiopia Ari Cultivator	East Africa Kenya Turkana	55.01		0.43	North Africa Egypt Egyptian	East Africa Ethiopia Ari Cultivator
21	East Africa Kenya Samburu	0	2D	2D	0.01	0.80	0.95	0.99	20.96	13.13-32.52	0.34	East Africa Ethiopia Anuak	East Africa Kenya Turkana	0.22	East Africa Ethiopia Ari Cultivator	East Africa Kenya Turkana	0.38	5.00	1.14-33.00	0.23	East Africa Kenya Turkana	East Africa Kenya Turkana	67.75	53.75-84.75	0.45	East Africa Ethiopia Anuak	North Africa Egypt Egyptian
21	East Africa Kenya Samburu	1	MW	MW	0.01	0.72	0.97	0.99	31.64	16.42-54.29	0.36	East Africa Ethiopia Anuak	East Africa Kenya Turkana	0.29	East Africa Kenya Turkana	East Africa Kenya Turkana	0.27	6.7		0.17	East Africa Kenya Turkana	East Africa Kenya Turkana	66.42		0.44	East Africa Ethiopia Anuak	North Africa Egypt Egyptian
21	East Africa Somalia Somali	0	2D	2D	0.01	0.89	0.96	1.00	37.01	26.93-46.71	0.27	North Africa Egypt Egyptian	East Africa Kenya Turkana	0.19	East Africa Ethiopia Ari Cultivator	East Africa Kenya Turkana	0.45	3.3	1.67-31.97	0.37	East Africa Kenya Turkana	East Africa Kenya Turkana	57.44	45.77-71.11	0.31	North Africa Egypt Egyptian	East Africa Kenya Turkana
21	East Africa Somalia Somali	1	1D	1D	0.01	0.88	0.98	1.00	40.36	26.39-47.18	0.28	North Africa Egypt Egyptian	East Africa Kenya Turkana	0.40	East Africa Kenya Turkana	East Africa Kenya Turkana	0.25	6.04		0.08	East Africa Kenya BantuKenya	East Africa Kenya Turkana	61.18		0.32	North Africa Egypt Egyptian	East Africa Kenya Turkana
21	East Africa Sudan Arab	0	2D	2D	0.01	0.97	1.00	1.00	23.54	20.14-27.12	0.45	East Africa Ethiopia Anuak	Middle East Saudi Arabia Saudi	0.45	East Africa Kenya Turkana	East Africa Kenya Turkana	0.45	15.48	2.67-41.81	0.45	Middle East Saudi Arabia Saudi	East Africa Kenya Turkana	66.4	50.07-89.07	0.47	North Africa Egypt Egyptian	East Africa Kenya Turkana
21	East Africa Sudan Arab	1	2D	2D	0.01	0.97	1.00	1.00	23.33	20.50-27.60	0.46	East Africa Kenya Turkana	Middle East Saudi Arabia Saudi	0.48	East Africa Kenya Turkana	East Africa Kenya Turkana	0.36	16.5	2.27-31.83	0.46	Middle East Saudi Arabia Saudi	East Africa Kenya Turkana	72.56	60.56-80.56	0.48	North Africa Egypt Egyptian	East Africa Kenya Turkana
21	East Africa Sudan Nubian	0	2D	2D	0.01	0.97	1.00	1.00	30.06	25.55-35.79	0.33	East Africa Ethiopia Anuak	North Africa Egypt Egyptian	0.40	North Africa Egypt Egyptian	East Africa Kenya Turkana	0.47	5.83	1.82-22.50	0.46	East Africa Kenya Turkana	North Africa Egypt Egyptian	43.95	29.28-61.62	0.34	East Africa Ethiopia Anuak	North Africa Egypt Egyptian
21	East Africa Sudan Nubian	1	2D	2D	0.01	0.96	1.00	1.00	29.72	23.01-36.07	0.32	East Africa Ethiopia Anuak	North Africa Egypt Egyptian	0.37	North Africa Egypt Egyptian	East Africa Kenya Turkana	0.46	7.23	1.28-24.23	0.43	East Africa Kenya Turkana	North Africa Egypt Egyptian	48.44	27.77-62.11	0.34	East Africa Ethiopia Anuak	North Africa Egypt Egyptian
22	East Africa Ethiopia Anuak	0	1D	1D	0.01	0.64	1.00	1.00	29.17	20.20-38.94	0.35	West Africa Nigeria Yoruba	East Africa Ethiopia Wolayta	0.28	West Africa Nigeria Yoruba	East Africa Kenya Luhya	0.11	1.17		0.47	East Africa Kenya Luhya	East Africa Kenya Luhya	28.87		0.49	East Africa Ethiopia Wolayta	East Africa Kenya Luhya
22	East Africa Ethiopia Anuak	1	1D	1D	0.01	0.53	1.00	1.00	32.92	22.09-45.29	0.41	West Africa Nigeria Yoruba	East Africa Ethiopia Wolayta	0.16	West Africa Nigeria Yoruba	East Africa Kenya Luhya	0.06	1.78		0.43	East Africa Kenya Luhya	East Africa Kenya Luhya	25.56		0.49	East Africa Ethiopia Wolayta	East Africa Kenya Luhya
22	East Africa Ethiopia Ari Blacksmith	0	1D	1D	0.01	0.31	1.00	1.00	29.54	14.81-64.48	0.04	East Africa Kenya Luhya	East Africa Ethiopia Wolayta	0.22	East Africa Ethiopia Oromo	East Africa Ethiopia Wolayta	0.02	20.37		0.05	East Africa Kenya BantuKenya	East Africa Ethiopia Wolayta	186.98		0.05	South Africa South African Bantu Soweto	East Africa Ethiopia Wolayta
22	East Africa Ethiopia Ari Blacksmith	1	1D	1D	0.01	0.19	0.99	0.99	44.36	16.42-185.56	0.04	East Africa Kenya Luhya	East Africa Ethiopia Wolayta	0.33	East Africa Ethiopia Wolayta	East Africa Ethiopia Wolayta	0.04	20.69		0.15	East Africa Ethiopia Wolayta	East Africa Ethiopia Wolayta	178.78		0.07	South Africa South African Bantu Soweto	East Africa Ethiopia Wolayta
22	East Africa Ethiopia Ari Cultivator	0	1D	1D	0.01	0.58	1.00	1.00	26.79	8.33-53.10	0.08	West Africa Nigeria Yoruba	East Africa Ethiopia Wolayta	0.47	East Africa Ethiopia Wolayta	East Africa Ethiopia Wolayta	0.14	5.5		0.15	East Africa Ethiopia Wolayta	East Africa Ethiopia Wolayta	85.49		0.07	East Africa Kenya Luhya	East Africa Ethiopia Wolayta
22	East Africa Ethiopia Ari Cultivator	1	1D	1D	0.01	0.41	1.00	1.00	80.88	42.82-131.10	0.07	West Africa Nigeria Yoruba	East Africa Ethiopia Wolayta	0.48	East Africa Ethiopia Wolayta	East Africa Ethiopia Wolayta	0.03	35.76		0.34	East Africa Ethiopia Wolayta	East Africa Ethiopia Wolayta	122.32		0.1	West Africa Nigeria Yoruba	East Africa Ethiopia Wolayta
22	East Africa Ethiopia Gumuz	0	1D	1D	0.01	0.60	0.98	0.99	6.38	3.19-17.47	0.35	East Africa Kenya Luhya	East Africa Ethiopia Wolayta	0.48	East Africa Ethiopia Wolayta	East Africa Ethiopia Wolayta	0.15	1.36		0.32	East Africa Kenya Luhya	East Africa Ethiopia Wolayta	68.54		0.44	East Africa Ethiopia Wolayta	East Africa Kenya Luhya
22	East Africa Ethiopia Gumuz	1	1D	1D	0.01	0.30	0.99	1.00	45.29	2.14-93.64	0.42	East Africa Kenya Luhya	East Africa Ethiopia Wolayta	0.37	East Africa Ethiopia Wolayta	East Africa Ethiopia Wolayta	0.06	4.3		0.32	East Africa Kenya Luhya	East Africa Ethiopia Wolayta	76.62		0.39	East Africa Kenya Luhya	East Africa Ethiopia Wolayta
22	East Africa Kenya Turkana	0	MW	MW	0.01	0.90	0.95	1.00	9.77	2.23-14.86	0.33	East Africa Kenya Luhya	East Africa Ethiopia Oromo	0.44	East Africa Ethiopia Oromo	East Africa Kenya Luhya	0.11	3.81		0.46	East Africa Kenya Luhya	East Africa Ethiopia Oromo	24.09		0.47	East Africa Kenya Luhya	East Africa Ethiopia Oromo
22	East Africa Kenya Turkana	1	MW	MW	0.01	0.81	0.97	1.00	10.63	3.35-16.01	0.30	East Africa Kenya Luhya	East Africa Ethiopia Oromo	0.41	East Africa Ethiopia Oromo	East Africa Ethiopia Wolayta	0.09	1.71		0.41	East Africa Kenya Luhya	East Africa Ethiopia Oromo	21.65		0.47	East Africa Ethiopia Oromo	East Africa Kenya Luhya
22	East Africa Sudan Sudanese	0	1D	1D	0.01	0.60	1.00	1.00	14.76	8.55-24.52	0.25	East Africa Kenya Luhya	East Africa Kenya Luhya	0.50	West Africa Nigeria Yoruba	East Africa Kenya Luhya	0.08	8.07		0.12	West Africa Nigeria Yoruba	East Africa Kenya Luhya	118.15		0.49	East Africa Kenya Luhya	East Africa Ethiopia Wolayta
22	East Africa Sudan Sudanese	1	1D	1D	0.01	0.52	1.00	1.00	14.32	5.18-53.43	0.23	West Africa Nigeria Yoruba	East Africa Kenya Luhya	0.47	East Africa Kenya Luhya	East Africa Kenya Luhya	0.08	6.06		0.14	West Africa Nigeria Yoruba	East Africa Kenya Luhya	107.8		0.45	East Africa Kenya Luhya	East Africa Ethiopia Wolayta
22	East Africa Tanzania Hadza	0	MW	MW	0.01	0.81	0.97	1.00	10.97	5.73-17.74	0.44	East Africa Kenya BantuKenya	East Africa Ethiopia Wolayta	0.44	East Africa Kenya Luhya	East Africa Kenya BantuKenya	0.18	1.64		0.43	East Africa Kenya BantuKenya	East Africa Tanzania Sandawe	26.29		0.41	East Africa Ethiopia Wolayta	East Africa Kenya BantuKenya
22	East Africa Tanzania Hadza	1	1D	1D	0.01	0.67	0.99	1.00	10.35	4.80-21.61	0.46	East Africa Kenya BantuKenya	East Africa Ethiopia Wolayta	0.33	East Africa Kenya BantuKenya	East Africa Kenya Luhya	0.08	1.00		0.45	East Africa Tanzania Sandawe	East Africa Kenya BantuKenya	21.87		0.44	East Africa Ethiopia Wolayta	East Africa Kenya BantuKenya

Supplementary table 3 – MALDER[27] analysis for each studied population using the parental populations inferred from GLOBETROTTER[26]. For each estimated admixture date (t0 and t1), the amplitude of the weighted LD curve (Amp) and the time in generation (Gen) are given with their repective standard error and Z-score.

Target	Source 1	Source 2	Amp_{t0} (+/- SE)	Z_{ampt0}	Gen_0 (+/- SE)	Z_{gent0}	$Ampt_1$ (+/- SE)	Z_{ampt1}	Gen_{t1} (+/- SE)	Z_{gent1}
Central Asia Afghanistan	East Asia China Mongolian	South Asia Pakistan Pathan	1.43E-04 +/- 1.17E-05	12.26	15.22 +/- 1.70	8.93				
Central Asia Afghanistan	Mainland Southeast Asia Myanmar Burmese	South Asia Pakistan Pathan	1.03E-04 +/- 9.59E-06	10.74	14.99 +/- 1.84	8.13				
Central Asia Uzbekstan Uzbek	East Asia China Mongolian	Middle East Turkey Turks	2.74E-04 +/- 2.03E-05	13.51	18.62 +/- 1.82	10.23				
East Africa Ethiopia Amhara	East Africa Kenya Turkana	North Africa Egypt Egyptian	7.26E-05 +/- 2.31E-05	3.15	51.32 +/- 12.20	4.20				
East Africa Ethiopia Amhara	North Africa Egypt Egyptian	East Africa Kenya Turkana	7.26E-05 +/- 2.31E-05	3.15	51.32 +/- 12.20	4.20				
East Africa Ethiopia Ari Blacksmith	East Africa Kenya Luhya	East Africa Ethiopia Wolayta	6.85E-05 +/- 2.07E-05	3.31	123.95 +/- 20.08	6.17				
East Africa Ethiopia Ethiopian Jews	East Africa Kenya Turkana	North Africa Egypt Egyptian	9.91E-05 +/- 1.93E-05	5.14	57.96 +/- 7.76	7.47				
East Africa Ethiopia Oromo	East Africa Kenya Turkana	East Africa Ethiopia Ari Cultivator	1.05E-05 +/- 1.59E-06	6.62	10.17 +/- 2.36	4.32				
East Africa Ethiopia Wolayta	East Africa Ethiopia Ari Cultivator	North Africa Egypt Egyptian	1.43E-04 +/- 2.56E-05	5.59	34.71 +/- 6.29	5.51				
East Africa Kenya BantuKenya	East Africa Ethiopia Anuak	South Africa South Africa BantuSA	1.90E-05 +/- 2.56E-06	7.44	17.65 +/- 3.18	5.55				
East Africa Kenya Luhya	East Africa Ethiopia Anuak	South Africa South Africa BantuSA	1.89E-05 +/- 1.90E-06	9.99	20.37 +/- 2.58	7.91				
East Africa Kenya Turkana	East Africa Ethiopia Oromo	East Africa Kenya Luhya	3.00E-05 +/- 5.19E-06	5.77	10.04 +/- 2.56	3.92				
East Africa Kenya Turkana	East Africa Kenya Luhya	East Africa Ethiopia Oromo	3.00E-05 +/- 5.19E-06	5.77	10.04 +/- 2.56	3.92				
East Africa Malagasy Mikea	Island Southeast Asia Indonesia Banjar	South Africa South African Bantu Soweto	1.07E-03 +/- 7.03E-05	15.16	23.66 +/- 1.70	13.94				
East Africa Malagasy Temoro	Island Southeast Asia Indonesia Banjar	South Africa South African Bantu Soweto	1.21E-03 +/- 4.84E-05	25.04	29.13 +/- 1.26	23.16				
East Africa Malagasy Vezo	Island Southeast Asia Indonesia Banjar	South Africa South African Bantu Soweto	1.15E-03 +/- 6.30E-05	18.32	23.14 +/- 1.40	16.48				
East Africa Somalia Somali	North Africa Egypt Egyptian	East Africa Kenya Turkana	4.81E-05 +/- 1.60E-05	3.00	56.29 +/- 14.45	3.89				
East Africa Sudan Arab	Middle East Saudi Arabia Saudi	East Africa Kenya Turkana	1.58E-04 +/- 1.21E-05	13.15	19.87 +/- 1.76	11.30				
East Africa Sudan Arab	North Africa Egypt Egyptian	East Africa Kenya Turkana	1.05E-04 +/- 8.91E-06	11.79	20.77 +/- 2.02	10.27				
East Africa Sudan Nubian	East Africa Ethiopia Anuak	North Africa Egypt Egyptian	4.87E-04 +/- 1.59E-04	3.07	78.21 +/- 22.08	3.54	1.73E-04 +/- 3.34E-05	5.17	12.27 +/- 1.85	6.62
East Africa Sudan Nubian	East Africa Kenya Turkana	North Africa Egypt Egyptian	8.80E-05 +/- 6.65E-06	13.23	21.93 +/- 2.17	10.13				
East Africa Tanzania Sandawe	East Africa Kenya BantuKenya	East Africa Ethiopia Wolayta	4.67E-05 +/- 7.31E-06	6.39	29.87 +/- 5.47	5.46				
East Africa Uganda Bakiga	East Africa Kenya Turkana	South Africa South Africa BantuSA	4.81E-05 +/- 9.99E-06	4.82	23.43 +/- 5.80	4.04				
East Asia China Oroqen	South Asia India Bengali	East Asia China Daur	2.66E-05 +/- 8.10E-06	3.28	27.92 +/- 9.03	3.09				
East Asia China Shor	Mainland Southeast Asia Myanmar Burmese	Middle East Turkey Turks	2.44E-04 +/- 3.76E-05	6.50	67.10 +/- 8.45	7.94	3.20E-05 +/- 7.26E-06	4.41	8.82 +/- 1.83	4.82
East Asia China Shor	North America USA CEU	East Asia China Mongolian	3.06E-04 +/- 3.99E-05	7.66	59.45 +/- 8.08	7.36	4.36E-05 +/- 1.48E-05	2.95	8.58 +/- 2.59	3.31
East Asia China Uygur	Middle East Turkey Turks	East Asia China Mongolian	2.84E-04 +/- 2.11E-05	13.41	19.25 +/- 1.93	9.95				
East Asia China Xibo	Central Asia Uzbekstan Uzbek	Mainland Southeast Asia Myanmar Burmese	1.81E-05 +/- 1.98E-06	9.13	9.39 +/- 1.66	5.66				
East Asia China Xibo	South Asia India Khasi	East Asia China Oroqen	1.99E-05 +/- 4.03E-06	4.95	12.27 +/- 3.72	3.30				
East Asia China Yi	Mainland Southeast Asia Malaysia Malay	Mainland Southeast Asia Myanmar Burmese	0.00E+00 +/- 0.00E+00	nan	17.79 +/- 4.60	3.87	4.74E-06 +/- 5.57E-07	8.51	25.35 +/- 3.93	6.45
Island Southeast Asia Indonesia Bajo	Melanesia Papua New Guinea PNG Highlander	Mainland Southeast Asia Malaysia Malay	5.74E-04 +/- 9.82E-05	5.85	83.50 +/- 11.98	6.97	7.71E-05 +/- 1.91E-05	4.05	8.33 +/- 2.20	3.78
Island Southeast Asia Indonesia Sumba	Melanesia Papua New Guinea PNG Highlander	Mainland Southeast Asia Malaysia Malay	8.25E-04 +/- 1.15E-04	7.19	82.78 +/- 5.55	14.92				
Island Southeast Asia Philippines Aeta	South Asia India Maratha	Island Southeast Asia Philippines Zambales	5.23E-05 +/- 1.01E-05	5.16	13.63 +/- 3.92	3.48				
Island Southeast Asia Philippines Batak	Melanesia Papua New Guinea 5	Island Southeast Asia Phillippines Filipino	2.56E-04 +/- 6.86E-05	3.73	73.51 +/- 10.93	6.73				
Island Southeast Asia Philippines Batak	South Asia India Bengali	Island Southeast Asia Phillippines Filipino	5.56E-05 +/- 1.27E-05	4.36	42.64 +/- 8.00	5.33				
Mainland Southeast Asia Cambodia Cambodian	East Asia China Uygur	Mainland Southeast Asia Malaysia Malay	1.20E-05 +/- 1.54E-06	7.84	18.41 +/- 2.83	6.51				
Mainland Southeast Asia Malaysia Bateq	Mainland Southeast Asia Malaysia Temuan	Mainland Southeast Asia Malaysia CheWong	8.06E-05 +/- 2.11E-05	3.83	43.54 +/- 9.89	4.40				
Mainland Southeast Asia Malaysia Kintaq	Mainland Southeast Asia Cambodia Cambodian	Mainland Southeast Asia Malaysia CheWong	1.04E-04 +/- 3.41E-05	3.06	47.93 +/- 11.49	4.17				
Mainland Southeast Asia Malaysia MahMeri	Mainland Southeast Asia Malaysia Mendriq	Mainland Southeast Asia Malaysia Malay	9.86E-06 +/- 2.64E-06	3.74	16.80 +/- 5.16	3.26				

Mainland Southeast Asia Myanmar Burmese	South Asia India Brahmin	East Asia China Mongolian	9.58E-05 +/- 5.99E-06	15.9 9	24.09 +/- 1.66	14.5 1				
Middle East Israel Bedouin	East Africa Sudan Arab	Middle East Turkey Sephardic Jews	4.77E-05 +/- 4.09E-06	11.6 6	26.33 +/- 2.36	11.1 4				
Middle East Jordania Jordanian	East Africa Kenya Turkana	Middle East Turkey Sephardic Jews	7.43E-05 +/- 6.81E-06	10.9 2	24.65 +/- 2.92	8.44				
Middle East Saudi Arabia Saudi	Middle East Turkey Turks	East Africa Sudan Arab	3.55E-05 +/- 8.92E-06	3.98	17.51 +/- 5.42	3.23				
Middle East Turkey Turks	East Asia China Mongolian	Middle East Lebanon Lebanese	1.25E-04 +/- 1.06E-05	11.8 1	28.88 +/- 3.30	8.76				
Middle East Yemen Yemeni	East Africa Kenya BantuKenya	Middle East Turkey Sephardic Jews	3.42E-04 +/- 4.25E-05	8.04	10.79 +/- 2.22	4.87				
Middle East Yemen Yemenite Jews	East Africa Ethiopia Ethiopians	Middle East Turkey Sephardic Jews	2.92E-05 +/- 4.74E-06	6.16	39.42 +/- 8.31	4.75				
North Africa Egypt Egyptian	East Africa Kenya Turkana	Middle East Turkey Sephardic Jews	1.30E-04 +/- 9.39E-06	13.8 3	21.44 +/- 2.33	9.20				
North Asia Russia Yakut	Caucasus Russia Lezgin	East Asia China Mongolian	6.21E-05 +/- 1.00E-05	6.20	6.18 +/- 1.06	5.83	1.24E-04 +/- 1.83E-05	6.81	51.33 +/- 7.51	6.83
North Asia Russia Yakut	South Asia India Gujarat Brahmin	East Asia China Mongolian	3.59E-05 +/- 6.82E-06	5.27	6.15 +/- 1.19	5.17	7.61E-05 +/- 1.20E-05	6.32	52.22 +/- 11.47	4.55
South Africa Namibia Juhoansi	East Africa Kenya Turkana	South Africa South African Bantu Soweto	4.32E-05 +/- 9.21E-06	4.69	61.57 +/- 11.54	5.34				
South Africa Namibia Nama	South Africa South Africa BantuSA	South Africa South African Bantu Soweto	1.71E-06 +/- 4.66E-07	3.67	10.27 +/- 2.59	3.96				
South Africa South Africa BantuSA	South Africa Namibia Nama	Central Africa Gabon Nzebi	7.34E-05 +/- 1.71E-05	4.30	23.24 +/- 5.91	3.93				
South Africa South Africa ColouredColesberg	North America USA CEU	South Africa South Africa Karretjie	9.92E-04 +/- 3.78E-05	26.2 7	5.90 +/- 0.40	14.6 0				
South Africa South Africa ColouredColesberg	South Africa South Africa Karretjie	South Africa South African Bantu Soweto	8.63E-05 +/- 1.37E-05	6.32	5.46 +/- 0.80	6.86	6.70E-05 +/- 1.88E-05	3.56	28.40 +/- 5.83	4.87
South Africa South Africa ColouredWellington	Mainland Southeast Asia Malaysia Malay	South Africa South Africa Karretjie	4.12E-04 +/- 6.16E-04	0.67	6.44 +/- 0.45	14.1 8	3.52E-04 +/- 6.13E-04	0.57	7.08 +/- 0.94	7.52
h Africa South Africa ColouredWellington	South Africa South African Bantu Soweto	South Asia India Brahmin	6.84E-04 +/- 2.41E-05	28.3 3	6.31 +/- 0.37	17.2 7				
South Africa South Africa Karretjie	North America USA CEU	South Africa South Africa SEBantu	4.55E-04 +/- 2.23E-05	20.4 1	4.50 +/- 0.40	11.3 1				
South Africa South Africa Khomani	East Africa Ethiopia Wolayta	South Africa South Africa SEBantu	8.96E-05 +/- 4.53E-06	19.7 9	5.46 +/- 0.48	11.4 4				
South Africa South Africa San	East Africa Ethiopia Wolayta	South Africa South Africa SEBantu	8.08E-05 +/- 4.97E-06	16.2 6	6.12 +/- 0.64	9.61				
South Africa South Africa San	North America USA CEU	South Africa South Africa SEBantu	3.69E-04 +/- 1.96E-05	18.8 1	5.27 +/- 0.50	10.4 8				
South Africa South Africa SEBantu	South Africa South Africa Karretjie	East Africa Kenya BantuKenya	1.37E-04 +/- 8.22E-06	16.7 0	20.47 +/- 1.38	14.8 5				
South Africa South Africa SWBantu	South Africa Namibia Nama	South Africa South Africa BantuSA	2.81E-05 +/- 5.88E-06	4.78	10.28 +/- 2.99	3.44				
South Africa South African Bantu Soweto	South Africa South Africa Karretjie	East Africa Kenya BantuKenya	1.42E-04 +/- 8.78E-06	16.2 2	24.23 +/- 1.78	13.6 1				
South Asia Bangladesh Bengali	South Asia India Khasi	South Asia India Kshatriya	6.24E-05 +/- 1.56E-05	4.00	61.69 +/- 17.84	3.46				
South Asia India Bengali	South Asia India Khasi	South Asia India Kshatriya	4.44E-05 +/- 6.91E-06	6.43	40.15 +/- 6.53	6.15				
South Asia India Birhor	Mainland Southeast Asia Cambodia Cambodian	South Asia India Maratha	7.52E-05 +/- 1.84E-05	4.10	69.37 +/- 17.62	3.94				
South Asia India Brahmin	Middle East Turkey Turks	South Asia India Maratha	7.20E-05 +/- 1.87E-05	3.85	69.31 +/- 13.21	5.25				
South Asia India Cochin Jews	Middle East Turkey Sephardic Jews	South Asia India Maratha	6.42E-05 +/- 9.23E-06	6.96	22.09 +/- 4.54	4.86				
South Asia India Gujarat Brahmin	Middle East Turkey Turks	South Asia India Maratha	1.14E-04 +/- 2.51E-05	4.54	91.61 +/- 12.34	7.42				
South Asia India Gujarati	Caucasus Russia Lezgin	South Asia India Maratha	1.05E-04 +/- 3.28E-05	3.21	96.62 +/- 18.74	5.16				
South Asia India Ho	Mainland Southeast Asia Cambodia Cambodian	South Asia India Maratha	1.01E-04 +/- 1.98E-05	5.11	81.59 +/- 14.05	5.81				
South Asia India Iyer	North America USA CEU	South Asia India Palian	1.47E-04 +/- 5.23E-05	2.81	101.55 +/- 20.15	5.04				
South Asia India Jamatia	South Asia India Bengali	East Asia China Tujia	6.81E-05 +/- 7.16E-06	9.52	29.82 +/- 3.70	8.06				
South Asia India Korva	Mainland Southeast Asia Cambodia Cambodian	South Asia India Maratha	8.76E-05 +/- 2.30E-05	3.80	78.70 +/- 18.15	4.34				
South Asia India Manipuri Brahmin	East Asia China Tujia	South Asia Bangladesh Bengali	1.42E-04 +/- 7.31E-06	19.4 1	11.56 +/- 0.97	11.8 9				
South Asia India Mumbai Jews	Middle East Lebanon Lebanese	South Asia India Maratha	1.03E-04 +/- 1.58E-05	6.50	27.89 +/- 6.00	4.65				
South Asia India Punjabi	Middle East Turkey Turks	South Asia India Maratha	4.36E-05 +/- 1.19E-05	3.67	41.22 +/- 11.52	3.58				
South Asia India Santal	Mainland Southeast Asia Cambodia Cambodian	South Asia India Maratha	1.06E-04 +/- 2.32E-05	4.56	89.53 +/- 16.86	5.31				
South Asia India Tharu	East Asia China Han NChina	South Asia Bangladesh Bengali	9.31E-05 +/- 7.81E-06	11.9 2	37.29 +/- 3.80	9.81				
South Asia India Tharu	East Asia China Tu	South Asia India Bengali	7.10E-05 +/- 1.66E-05	4.27	37.56 +/- 8.52	4.41				
South Asia India Tripuri	South Asia Bangladesh Bengali	East Asia China Tujia	5.71E-05 +/- 6.94E-06	8.24	20.89 +/- 3.66	5.71				
South Asia Pakistan Balochi	South Africa South African Bantu Soweto	South Asia India Brahmin	6.18E-05 +/- 1.08E-05	5.71	10.48 +/- 2.98	3.52				
South Asia Pakistan Brahui	East Africa Kenya BantuKenya	South Asia India Brahmin	5.71E-05 +/- 6.58E-06	8.68	8.68 +/- 1.69	5.13				

South Asia Pakistan Brahui	Middle East Turkey Turks	South Asia India Kshatriya	1.62E-05 +/- 3.74E-06	4.34	36.09 +/- 8.70	4.15				
South Asia Pakistan Burusho	East Asia China Tu	South Asia India Brahmin	7.50E-05 +/- 1.17E-05	6.43	46.49 +/- 7.27	6.40				
South Asia Pakistan Hazara	East Asia China Mongolian	Middle East Turkey Turks	2.88E-04 +/- 1.45E-05	19.91	19.50 +/- 1.22	16.03				
South Asia Pakistan Kalash	Middle East Turkey Turks	South Asia India Brahmin	1.53E-05 +/- 5.60E-06	2.74	61.37 +/- 19.19	3.20				
South Asia Pakistan Makrani	East Africa Kenya BantuKenya	South Asia India Brahmin	1.68E-04 +/- 1.25E-05	13.51	12.84 +/- 1.43	8.98				
South Asia Pakistan Makrani	South Africa South African Bantu Soweto	South Asia India Brahmin	1.88E-04 +/- 1.48E-05	12.69	12.66 +/- 1.50	8.41				
South Asia Pakistan Sindhi	Middle East Turkey Turks	South Asia India Kshatriya	3.48E-05 +/- 8.54E-06	4.07	70.79 +/- 11.84	5.98				
South Asia Pakistan Sindhi	North Africa Egypt Egyptian	South Asia India Gujarat Brahmin	5.22E-06 +/- 6.12E-07	8.54	6.07 +/- 1.18	5.14				

Supplementary table 4 – Summary table of the number of migrations per century estimated by GLOBETROTTER[26] and MALDER[27].

Century	MALDER	GLOBETROTTER
2th	4	2
3th	6	18
4th	0	14
5th	8	14
6th	4	12
7th	6	14
8th	4	12
9th	4	5
10th	6	18
11th	8	14
12th	0	14
13th	15	32
14th	17	36
15th	22	22
16th	12	38
17th	26	44
18th	20	48

Supplementary table 5 – (A) Correlation tests between the volume of trade or time (century) and the number of migrations, the average distance of migration and its variance, based on GLOBETROTTER[26] (#) and MALDER[27] (##) results. Significant tests based on GLOBETROTTER[26] were corrected by the number of tests (n=6) and are indicated in column P (Bonferroni multiple testing correction). P_n corresponds to nominal p-values. As MALDER[27] analyses were based on specific hypotheses driven by GLOBETROTTER[26] results, we report only nominal p-values. (B) Table of the F-test between the linear regressions of the number of migrations using time (century) or the volume of trade. SSV: Sum of Squares of Residuals (Model Volume of Trade); SSC: Sum of Squares of Residuals (Model Century).
A.

	Century			Volume of trade		
	r^2	P_n	P	r^2	P_n	P
Number of migrations #	0.82	0.00006	0.00036	0.89	0.000002	0.000012
Number of migrations ##	0.79	0.0001	-	0.76	0.0003	-
Average distance of migration #	0.06	0.72	-	-0.04	0.98	-
Variance of distance of migration #	0.31	0.06	-	0.43	0.08	-

B.

Sum of Squares of Residuals (Model Century)	Sum of Squares of Residuals (Model Volume of Trade)	F (SSV/SSC)	*P*
2108.77	580.97	3.63	0.009

Supplementary table 6 – ANOVA (F) and t-tests (t) of the number of migrations, the average distance of migration and its variance between the four phases described by historical data, based on GLOBETROTTER[26] results. ANOVA p-values were corrected by the number of tests (n=3) and are indicated in column P (Bonferroni multiple testing correction). Given the ANOVA results, t-tests were based on specific hypotheses, so only nominal p-value (P_n) are reported.

	Four Phases		Phases I-II		Phases II-III		Phases III-IV	
	F	P	t	P_n	t	P_n	t	P_n
Number of migrations	10.39	0.0006	1.17	0.27	-2.25	0.043	-2.39	0.03
Average distance of migration	5.34	0.012	2.92	0.01	-3.36	0.004	1.42	0.18
Variance of distance of migration	4.33	0.03	2.34	0.03	-3.43	0.009	1.77	0.11

Supplementary table 7 – *f*3-statistics and F_{ST} distances matrix between populations in the same clusters defined by the fineSTRUCTURE[1] analysis. The "*f*3" column identifies populations showing only positive values ("+") or at least one negative value ("-"). The "ID" row and column identify each population by a numerical code. *: Populations identified as outliers according to F_{ST} distances that are one standard deviation beyond the mean of its cluster, have a positive *f*3 value, and no characterized recent history of admixture in previous papers.

Cluster 1	*f*3	ID	138	148	154	151
Mainland Southeast Asia Malaysia Malay	-	138				
Island Southeast Asia Indonesia Banjar	-	148	0.003			
Island Southeast Asia Indonesia SKDayak	-	154	0.004	0.002		
Island Southeast Asia Indonesia Ma'anyan *	+	151	0.01	0.007	0.006	

Cluster 2		ID	149	152	153	147	155	159	161	162	163
Island Southeast Asia Indonesia Dusun	+	149									
Island Southeast Asia Indonesia Mandar	-	152	0.016								
Island Southeast Asia Indonesia Murut	+	153	0.022	0.021							
Island Southeast Asia Indonesia Bajo	-	147	0.022	0.011	0.027						
Island Southeast Asia Indonesia Sumba	-	155	0.025	0.012	0.031	0.01					
Island Southeast Asia Philippines Casigurans	-	159	0.014	0.007	0.019	0.014	0.017				
Island Southeast Asia Philippines Zambales	-	161	0.014	0.007	0.018	0.014	0.016	0.003			
Island Southeast Asia Phillippines Filipino	-	162	0.013	0.006	0.018	0.012	0.015	0.001	0.001		
Island Southeast Asia Phillippines Igorot *	+	163	0.039	0.033	0.043	0.042	0.045	0.025	0.026	0.026	

Cluster 3		ID	133	137	156	157	140	134	141	150	158	160
Mainland Southeast Asia Malaysia CheWong	+	133										
Mainland Southeast Asia Malaysia MahMeri	+	137	0.089									
Island Southeast Asia Philippines Aeta	+	156	0.109	0.106								
Island Southeast Asia Philippines Agta	+	157	0.112	0.109	0.07							
Mainland Southeast Asia Malaysia Seletar	+	140	0.13	0.123	0.136	0.139						
Mainland Southeast Asia Malaysia Jakun	+	134	0.062	0.059	0.081	0.083	0.097					
Mainland Southeast Asia Malaysia Temuan	+	141	0.055	0.038	0.074	0.076	0.089	0.024				
Island Southeast Asia Indonesia Lebbo	+	150	0.084	0.077	0.085	0.09	0.11	0.051	0.042			
Island Southeast Asia Philippines Batak	+	158	0.09	0.083	0.069	0.071	0.114	0.058	0.051	0.06		

Island Southeast Asia Philippines Tagbanua	-	160	0.074	0.066	0.058	0.061	0.097	0.041	0.033	0.041	0.018

Cluster 4		ID	132	135	136	139
Mainland Southeast Asia Malaysia Bateq	+	132				
Mainland Southeast Asia Malaysia Jehai	+	135	0.047			
Mainland Southeast Asia Malaysia Kintaq	+	136	0.046	0.029		
Mainland Southeast Asia Malaysia Mendriq	-	139	0.027	0.027	0.028	

Cluster 5		ID	164	165	166	167	168
Melanesia Papua New Guinea Bougainville	+	164					
Melanesia Papua New Guinea Koinambe	+	165	0.107				
Melanesia Papua New Guinea Kosipe	+	166	0.113	0.05			
Melanesia Papua New Guinea	+	167	0.094	0.021	0.038		
Melanesia Papua New Guinea PNG Highlander	+	168	0.097	0.022	0.038	0.008	

Cluster 6		ID	107	174	175	177	180	143	145	146	169	170	173	176	179	171	172	182	183	185	186	187
South Asia India Naga *	+	107																				
East Asia China Lahu *	+	174	0.039																			
East Asia China Miao	+	175	0.025	0.024																		
East Asia China Naxi	+	177	0.02	0.027	0.013																	
East Asia China She	+	180	0.031	0.03	0.012	0.017																
Mainland Southeast Asia Cambodia Cambodian	-	143	0.032	0.022	0.013	0.019	0.018															
Mainland Southeast Asia Vietnam Kinh	-	145	0.029	0.019	0.007	0.014	0.011	0.005														
Mainland Southeast Asia Vietnam Vietnamese	-	146	0.028	0.02	0.007	0.014	0.011	0.006	0													
East Asia China Dai	-	169	0.031	0.021	0.008	0.016	0.012	0.007	0.002	0.002												
East Asia China Daur	-	170	0.025	0.033	0.015	0.014	0.02	0.023	0.017	0.017	0.02											
East Asia China Hezhen	-	173	0.024	0.03	0.013	0.012	0.017	0.021	0.014	0.014	0.017	0.002										
East Asia China Mongolian	-	176	0.02	0.026	0.009	0.009	0.012	0.016	0.01	0.01	0.013	0.003	0.003									
East Asia China S Han	-	179	0.023	0.022	0.005	0.01	0.008	0.011	0.003	0.003	0.005	0.011	0.009	0.005								
East Asia China Han	-	171	0.02	0.023	0.006	0.008	0.009	0.013	0.006	0.005	0.008	0.009	0.006	0.003	0.001							
East Asia China Han NChina	-	172	0.018	0.024	0.007	0.007	0.011	0.014	0.008	0.008	0.011	0.008	0.005	0.002	0.002	0.001						
East Asia China Tu	-	182	0.016	0.025	0.009	0.007	0.013	0.015	0.011	0.011	0.014	0.007	0.006	0.002	0.005	0.003	0.002					
East Asia China Tujia	-	183	0.021	0.022	0.004	0.008	0.009	0.012	0.005	0.004	0.007	0.011	0.008	0.004	0.001	0.001	0.002	0.004				

East Asia China Xibo	-	185	0.02	0.027	0.011	0.01	0.014	0.017	0.012	0.011	0.015	0.003	0.003	0.001	0.006	0.004	0.002	0.003	0.005		
East Asia China Yi	-	186	0.017	0.024	0.01	0.006	0.016	0.017	0.012	0.012	0.014	0.012	0.01	0.007	0.007	0.006	0.005	0.004	0.007	0.008	
East Asia Japan Japanese	+	187	0.026	0.031	0.014	0.015	0.017	0.021	0.014	0.014	0.017	0.01	0.008	0.006	0.009	0.007	0.007	0.009	0.009	0.006	0.013
Cluster 7		ID	69	72	125	184	178	188													
Central Asia Afghanistan	-	69																			
Central Asia Uzbekstan Uzbek	-	72	0.002																		
South Asia Pakistan Hazara	-	125	0.005	0.005																	
East Asia China Uygur	-	184	0.004	0.003	0.004																
East Asia China Oroqen	-	178	0.044	0.036	0.031	0.028															
North Asia Russia Yakut	-	188	0.039	0.032	0.029	0.026	0.01														
Cluster 8		ID	84	93	97	103	117	118	99												
South Asia India Garo	-	84																			
South Asia India Jamatia	-	93	0.015																		
South Asia India Khasi	-	97	0.017	0.015																	
South Asia India Manipuri Brahmin	-	103	0.02	0.016	0.012																
South Asia India Tharu	-	117	0.022	0.019	0.017	0.015															
South Asia India Tripuri	-	118	0.01	0.005	0.01	0.011	0.015														
South Asia India Kol	-	99	0.047	0.043	0.037	0.031	0.035	0.037													
Cluster 9		ID	76	80	85	94	109	111	113												
South Asia India Bonda	+	76																			
South Asia India Chenchus	+	80	0.055																		
South Asia India Gond	-	85	0.016	0.037																	
South Asia India Jarawa	+	94	0.119	0.144	0.112																
South Asia India Onge	+	109	0.135	0.161	0.129	0.025															
South Asia India Paniya	+	111	0.054	0.07	0.038	0.144	0.16														
South Asia India Pulliyar	+	113	0.068	0.082	0.05	0.158	0.176	0.077													
Cluster 10		ID	75	89	91	95	100	116													
South Asia India Birhor	+	75																			
South Asia India Ho	-	89	0.028																		

South Asia India Irula	+	91	0.058	0.034														
South Asia India Kadar	+	95	0.055	0.031	0.025													
South Asia India Korva	+	100	0.037	0.017	0.048	0.044												
South Asia India Santal	+	116	0.028	0.004	0.035	0.032	0.017											
Cluster 11		ID	73	74	79	80	90	99	131	104	110	120	88	112	115	108	102	
South Asia Bangladesh Bengali	-	73																
South Asia India Bengali	-	74	0															
South Asia India Chamar	-	79	0.004	0.003														
South Asia India Chenchus	+	80	0.005	0.004	0.006													
South Asia India Indian	-	90	0.002	0.002	0.004	0.004												
South Asia India Kol	-	99	0.006	0.007	0.005	0.009	0.007											
South Asia SriLanka Sri Lanka UK	-	131	0.002	0.002	0.004	0.004	0.001	0.008										
South Asia India Maratha	-	104	0.002	0.001	0.003	0.003	0.001	0.006	0.001									
South Asia India Palian	-	110	0.003	0.003	0.004	0.005	0.002	0.008	0.002	0.001								
South Asia India Velama	-	120	0.008	0.008	0.01	0.01	0.005	0.014	0.007	0.007	0.008							
South Asia India Hakkipikki	+	88	0.011	0.012	0.012	0.013	0.01	0.016	0.01	0.008	0.01	0.017						
South Asia India Piramalai Kallar	-	112	0.01	0.01	0.012	0.012	0.009	0.015	0.009	0.008	0.009	0.014	0.018					
South Asia India Sakilli	+	115	0.015	0.015	0.016	0.016	0.013	0.019	0.013	0.011	0.013	0.019	0.02	0.021				
South Asia India North Kannadi *	+	108	0.017	0.016	0.017	0.017	0.015	0.02	0.015	0.013	0.015	0.022	0.02	0.023	0.025			
South Asia India Kurumba *	+	102	0.02	0.019	0.021	0.022	0.018	0.025	0.019	0.018	0.02	0.024	0.028	0.025	0.03	0.032		
Cluster 12		ID	77	78	81	82	83	86	87	92	96	101	105	106	114	119	121	129
South Asia India Brahmin	-	77																
South Asia India Brahmins from Uttar Pradesh	-	78	0.000															
South Asia India Cochin Jews	+	81	0.019	0.019														
South Asia India Dharkar	-	82	0.011	0.011	0.029													
South Asia India Dusadh *	+	83	0.052	0.051	0.070	0.061												
South Asia India Gujarat Brahmin	-	86	0.001	0.001	0.019	0.011	0.052											
South Asia India Gujarati	-	87	0.003	0.003	0.021	0.012	0.053	0.002										
South Asia India Iyer	-	92	0.002	0.001	0.019	0.012	0.052	0.002	0.003									
South Asia India Kanjars	-	96	0.009	0.008	0.027	-0.000	0.057	0.009	0.010	0.010								

South Asia India Kshatriya	-	101	0.001	0.000	0.018	0.011	0.052	0.001	0.002	0.002	0.009						
South Asia India Mumbai Jews	+	105	0.026	0.026	0.042	0.037	0.078	0.025	0.029	0.027	0.035	0.026					
South Asia India Muslim	-	106	0.002	0.002	0.018	0.011	0.051	0.002	0.003	0.002	0.008	0.001	0.027				
South Asia India Punjabi	-	114	0.002	0.002	0.019	0.012	0.052	0.002	0.003	0.002	0.009	0.002	0.027	0.002			
South Asia India Uttar Pradesh Scheduled Caste	-	119	0.007	0.007	0.026	0.016	0.056	0.007	0.008	0.008	0.014	0.007	0.034	0.007	0.008		
South Asia India West Bengal Brahmin	-	121	0.001	0.000	0.019	0.011	0.051	0.001	0.003	0.002	0.009	0.001	0.027	0.001	0.002	0.007	
South Asia Pakistan Punjabi	-	129	0.005	0.005	0.023	0.014	0.054	0.005	0.006	0.005	0.011	0.005	0.031	0.004	0.002	0.010	0.005
Cluster 13		ID	98	122	123	124	126	127	128	130							
South Asia India Khatri	-	98															
South Asia Pakistan Balochi	-	122	0.007														
South Asia Pakistan Brahui	-	123	0.009	0.003													
South Asia Pakistan Burusho	-	124	0.008	0.011	0.013												
South Asia Pakistan Kalash	+	126	0.031	0.032	0.033	0.032											
South Asia Pakistan Makrani	-	127	0.009	0.002	0.004	0.012	0.033										
South Asia Pakistan Pathan	-	128	0.004	0.005	0.006	0.006	0.028	0.006									
South Asia Pakistan Sindhi	-	130	0.003	0.004	0.006	0.008	0.03	0.005	0.003								
Cluster 14		ID	49	51	53	55	56	57	58	61	62						
North Africa Egypt Egyptian	-	49															
Middle East Israel Bedouin	-	51	0.005														
Middle East Israel Palestinian	-	53	0.004	0.008													
Middle East Jordania Jordanian	-	55	0.002	0.006	0.003												
Middle East Lebanon Lebanese	-	56	0.004	0.008	0.005	0.003											
Middle East Saudi Arabia Saudi	-	57	0.006	0.005	0.008	0.006	0.008										
Middle East Syria Syrians	-	58	0.004	0.007	0.005	0.003	0.005	0.006									
Middle East Yemen Yemeni	-	61	0.007	0.011	0.01	0.009	0.01	0.01	0.01								
Middle East Yemen Yemenite Jews	+	62	0.008	0.01	0.01	0.008	0.01	0.008	0.009	0.014							
Cluster 15		ID	50	52	59	60	63	64	65	66	67	68	70	71	189	54	
Middle East Iraq Iraqi Jews	+	50															
Middle East Israel Druze	+	52	0.012														
Middle East Turkey Sephardic Jews	-	59	0.008	0.006													

Middle East Turkey Turks	-	60	0.008	0.007	0.003										
Caucasus Armenia Armenian	-	63	0.008	0.007	0.003	0.001									
Caucasus Azerbaijan Azerbaijani Jews	+	64	0.017	0.017	0.013	0.013	0.012								
Caucasus Georgia Georgian	+	65	0.012	0.01	0.007	0.004	0.003	0.015							
Caucasus Georgia Georgian Jews	+	66	0.016	0.016	0.012	0.011	0.011	0.02	0.013						
Caucasus Georgia Georgians	-	67	0.011	0.01	0.006	0.003	0.002	0.015	0	0.013					
Caucasus Russia Lezgin	-	68	0.014	0.013	0.009	0.005	0.005	0.017	0.007	0.016	0.005				
Central Asia Iran Iranian	-	70	0.009	0.009	0.006	0.003	0.003	0.014	0.006	0.013	0.005	0.006			
Central Asia Iran Iranian Jew	+	71	0.013	0.015	0.012	0.011	0.011	0.018	0.014	0.017	0.014	0.016	0.012		
North America USA CEU	+	189	0.019	0.016	0.008	0.009	0.009	0.022	0.014	0.021	0.011	0.01	0.013	0.022	
Middle East Israel Samaritians *	+	54	0.064	0.063	0.058	0.06	0.06	0.071	0.064	0.068	0.064	0.067	0.061	0.066	0.07
Cluster 16		ID	13	14	37	38	39								
South Africa South Africa ColouredColesberg	-	13													
South Africa South Africa ColouredWellington	-	14	0.004												
East Africa Malagasy Mikea	-	37	0.027	0.025											
East Africa Malagasy Temoro	-	38	0.021	0.019	0.008										
East Africa Malagasy Vezo	-	39	0.022	0.019	0.007	0.004									
Cluster 17		ID	2	3	4	5	46	48							
Central Africa CAR Biaka Pygmy	+	2													
Central Africa DR Congo Mbuti Pygmy	+	3	0.056												
Central Africa Gabon Baka	+	4	0.011	0.058											
Central Africa Gabon Bongo	-	5	0.018	0.054	0.017										
East Africa Tanzania Sandawe	-	46	0.05	0.078	0.046	0.033									
East Africa Uganda Batwa	+	48	0.049	0.051	0.048	0.039	0.052								
Cluster 18		ID	1	6	19	33	34	47							
West Africa Nigeria Yoruba	+	1													
Central Africa Gabon Nzebi	-	6	0.005												
South Africa South Africa SWBantu	-	19	0.014	0.011											
East Africa Kenya BantuKenya	-	33	0.008	0.006	0.015										
East Africa Kenya Luhya	-	34	0.007	0.006	0.014	0.001									

East Africa Uganda Bakiga	-	47	0.009	0.007	0.015	0.005	0.005								
Cluster 19		ID	7	9	10	11	15	16	17	8	45				
South Africa Botswana GuiGhanaKgal	-	7													
South Africa Namibia Nama	-	9	0.017												
South Africa South Africa/Namibia Khwe	+	10	0.025	0.023											
South Africa South Africa/Namibia Xun	+	11	0.019	0.019	0.027										
South Africa South Africa Karretjie	-	15	0.018	0.009	0.031	0.02									
South Africa South Africa Khomani	-	16	0.014	0.002	0.024	0.017	0.005								
South Africa South Africa San	-	17	0.014	0.005	0.029	0.017	0.005	0							
South Africa Namibia Juhoansi	-	8	0.031	0.03	0.05	0.016	0.028	0.026	0.024						
East Africa Tanzania SAN NB	-	45	0.032	0.031	0.052	0.016	0.029	0.027	0.024	-0.015					
Cluster 20		ID	18	20	12										
South Africa South Africa SEBantu	-	18													
South Africa South African Bantu Soweto	-	20	0												
South Africa South Africa BantuSA	-	12	0	0.001											
Cluster 21		ID	21	22	26	27	29	30	31	32	35	36	40	41	42
East Africa Ethiopia Afar	-	21													
East Africa Ethiopia Amhara	-	22	0.006												
East Africa Ethiopia Ethiopian Jews	-	26	0.011	0.006											
East Africa Ethiopia Ethiopians	-	27	0.006	0.001	0.006										
East Africa Ethiopia Oromo	-	29	0.006	0.002	0.006	0.002									
East Africa Ethiopia Somali	-	30	0.009	0.007	0.012	0.007	0.004								
East Africa Ethiopia Tygray	-	31	0.005	0.001	0.006	0	0.003	0.007							
East Africa Ethiopia Wolayta	-	32	0.011	0.006	0.01	0.007	0.003	0.008	0.007						
East Africa Kenya Samburu	-	35	0.015	0.012	0.014	0.012	0.006	0.008	0.012	0.008					
East Africa Kenya Turkana	-	36	0.016	0.022	0.02	0.019	0.012	0.015	0.017	0.016	0.004				
East Africa Somalia Somali	-	40	0.011	0.009	0.013	0.009	0.005	0	0.009	0.01	0.008	0.016			
East Africa Sudan Arab	-	41	0.01	0.006	0.011	0.005	0.007	0.011	0.006	0.012	0.014	0.021	0.013		
East Africa Sudan Nubian	-	42	0.01	0.006	0.01	0.005	0.007	0.011	0.005	0.012	0.014	0.02	0.013	0.003	

Cluster 22		ID	23	24	25	28	43	44
East Africa Ethiopia Anuak	-	23						
East Africa Ethiopia Ari Blacksmith	+	24	0.049					
East Africa Ethiopia Ari Cultivator	-	25	0.032	0.021				
East Africa Ethiopia Gumuz	+	28	0.019	0.046	0.029			
East Africa Sudan Sudanese	+	43	0.002	0.053	0.035	0.022		
East Africa Tanzania Hadza	+	44	0.062	0.08	0.063	0.066	0.064	

Supplementary figure 1 – EEMS[28] gradient map of the analysed dataset after convergence of 3×10^6 MCMC iterations. The color scale reveals low (blue) and high (orange) genetic barriers between populations localized on a grid of 1,000 demes. Each deme (green dot) is proportional to the number of populations included. The map was generated with the R package *rworldmap* (https://cran.r-project.org/web/packages/rworldmap/index.html).

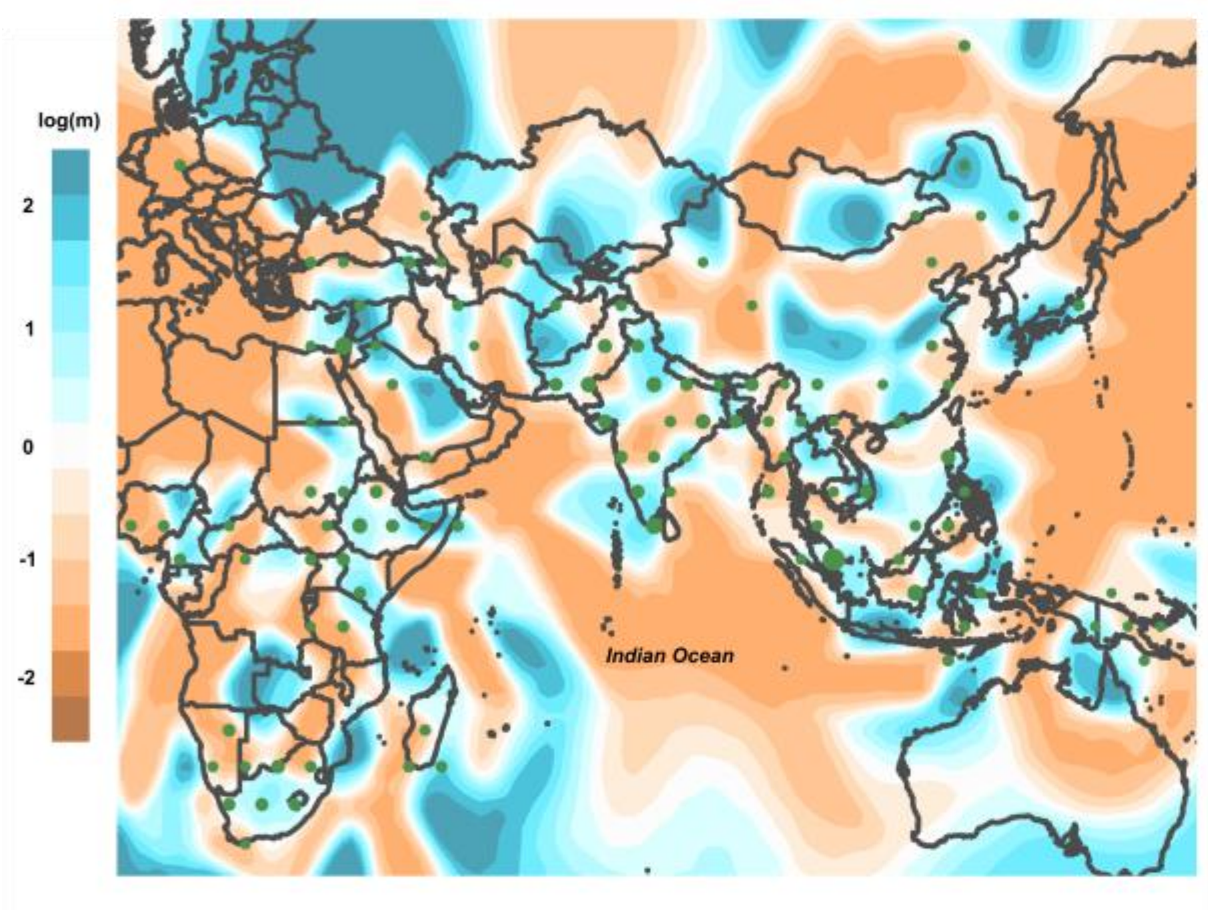

Supplementary figure 2 – EEMS[28] posterior probability plot obtained after 3 x 10^6 MCMC iterations.

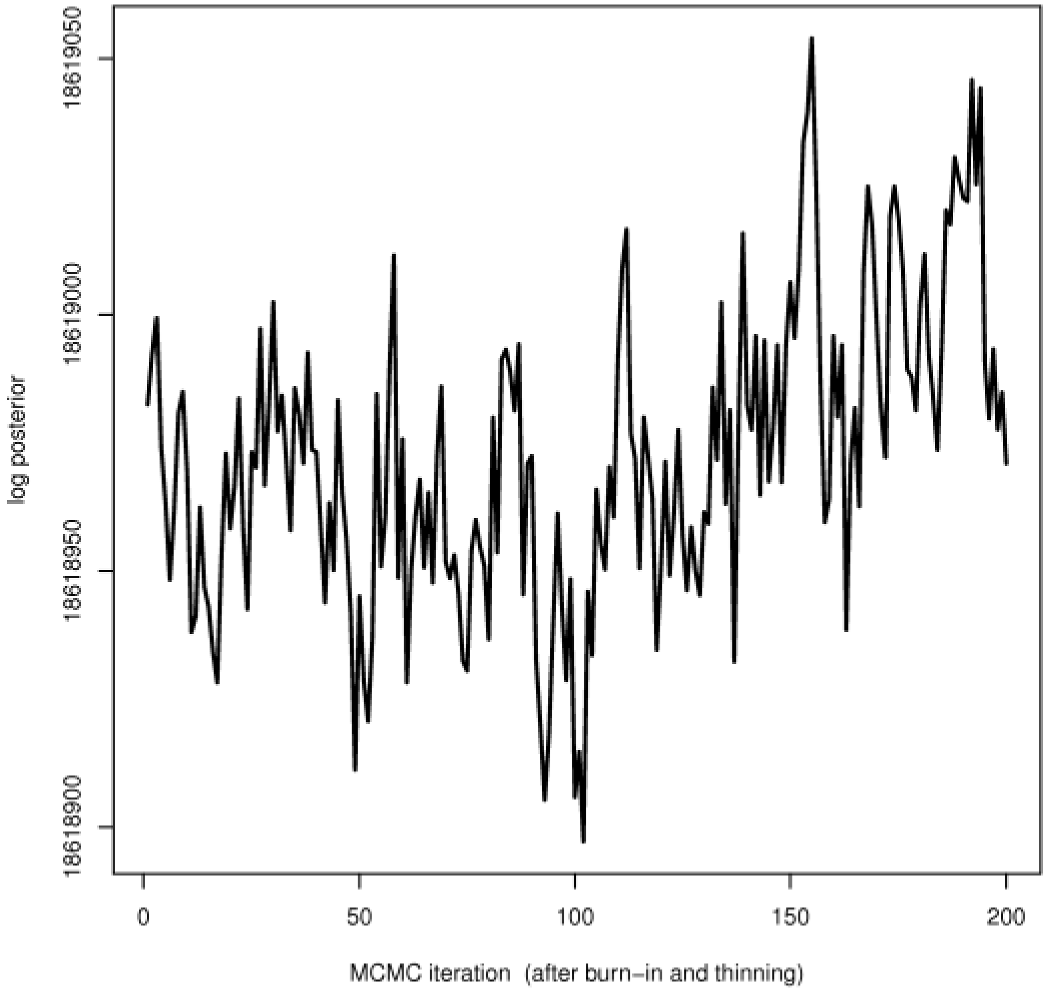

Supplementary figure 3 – Coancestry heat map generated by fineSTRUCTURE[1] of the individuals included in the dataset. The color scale indicates the chunk counts between each pair of individuals calculated by CHROMOPAINTER[1]. The numerical labels identify each cluster including the populations detailed in Supplementary Table 1.

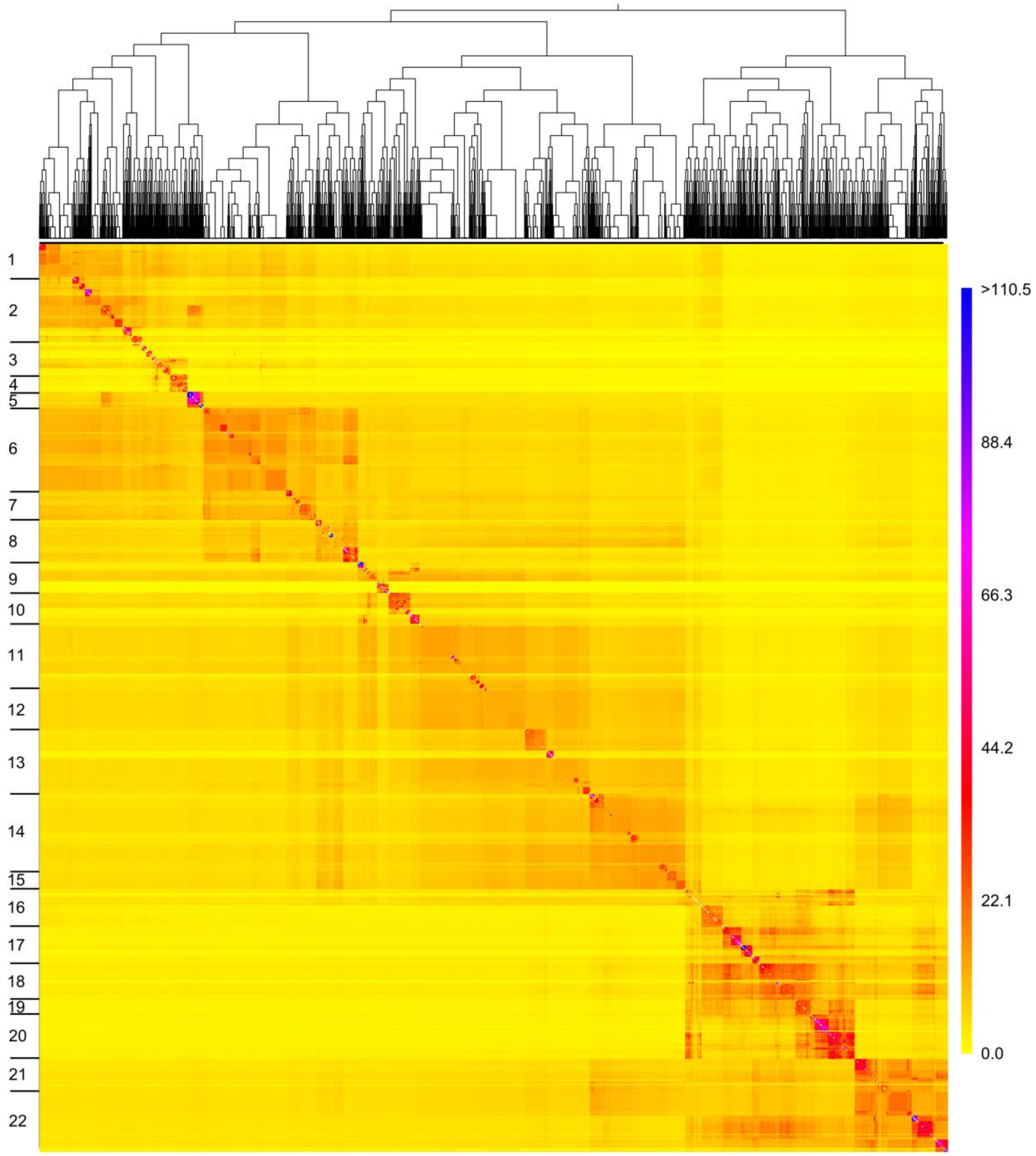

Supplementary figure 4 – Simplified ADMIXTURE[29] plot for the major mode of K=29, defined by CLUMPAK[30]. The plot is split in 22 regional clusters of populations, marked by white lines, using the clustering defined by fineSTRUCTURE[1]. Each colored line is a population whose genetic background can be decomposed into 29 genetic components. The numerical code corresponds to the one detailed for Supplementary Table 1.

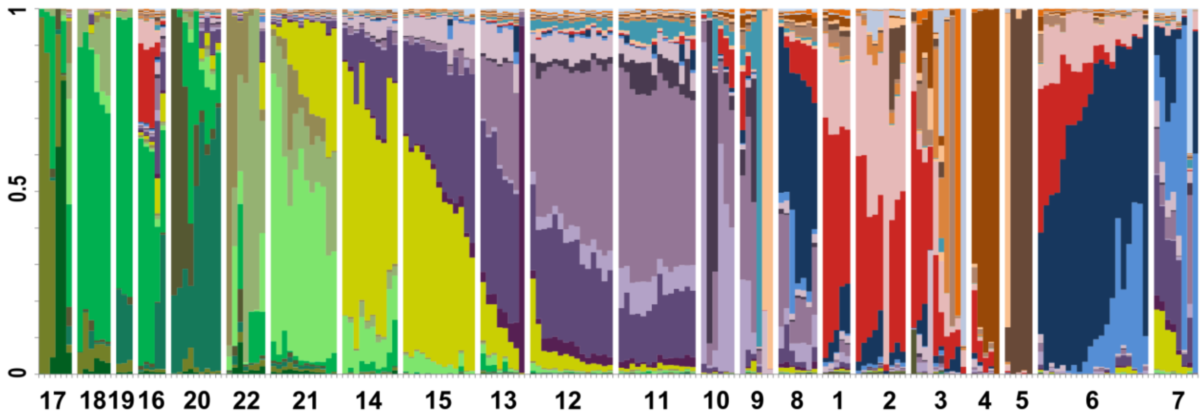

Supplementary figure 5 – ADMIXTURE[29] plots obtained with CLUMPAK[30] (major modes) from K=2 to K=36 and visualized with Genesis[31]. Each column is an individual, whose genotype data has been decomposed into K or fewer genetic components (colors).

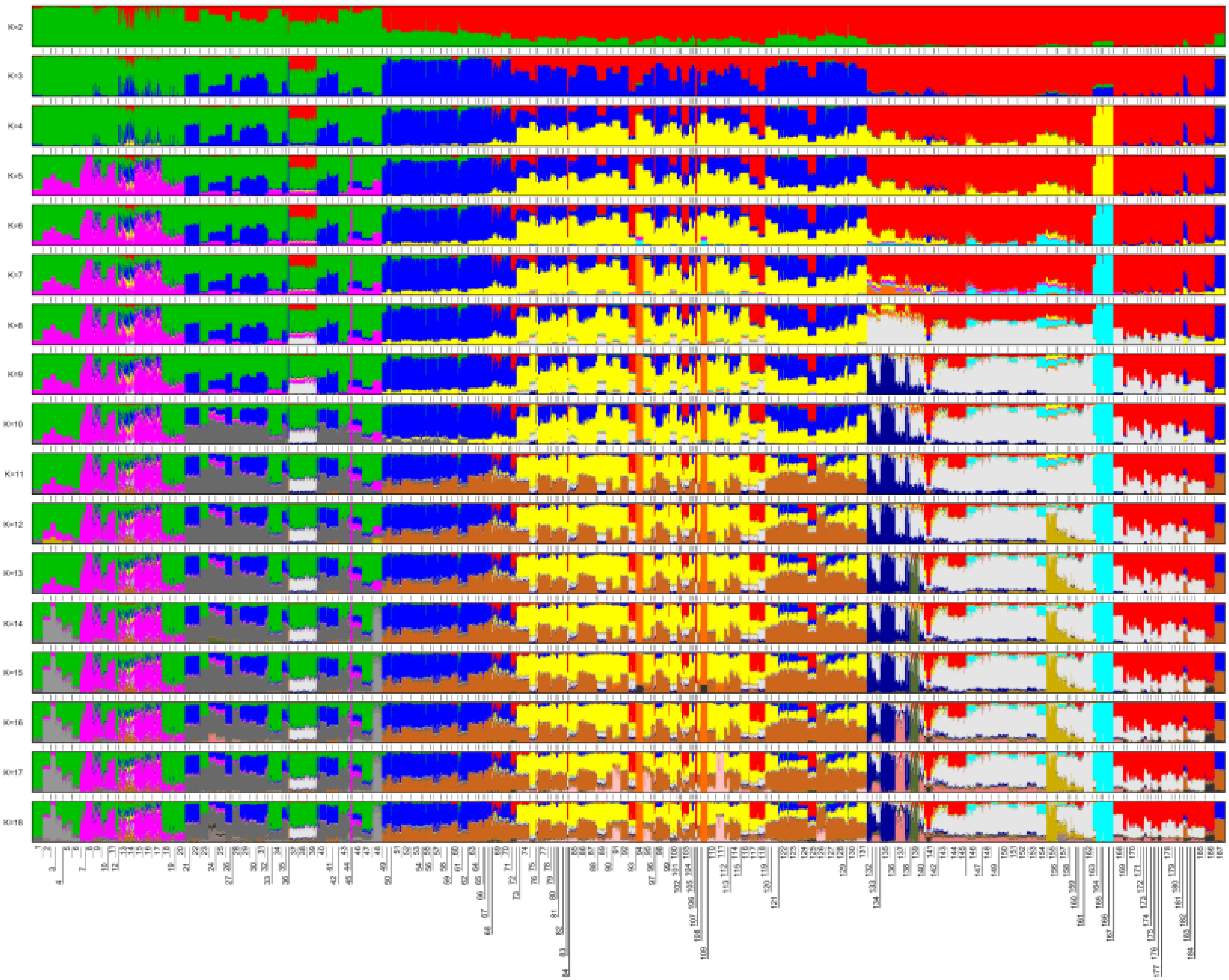

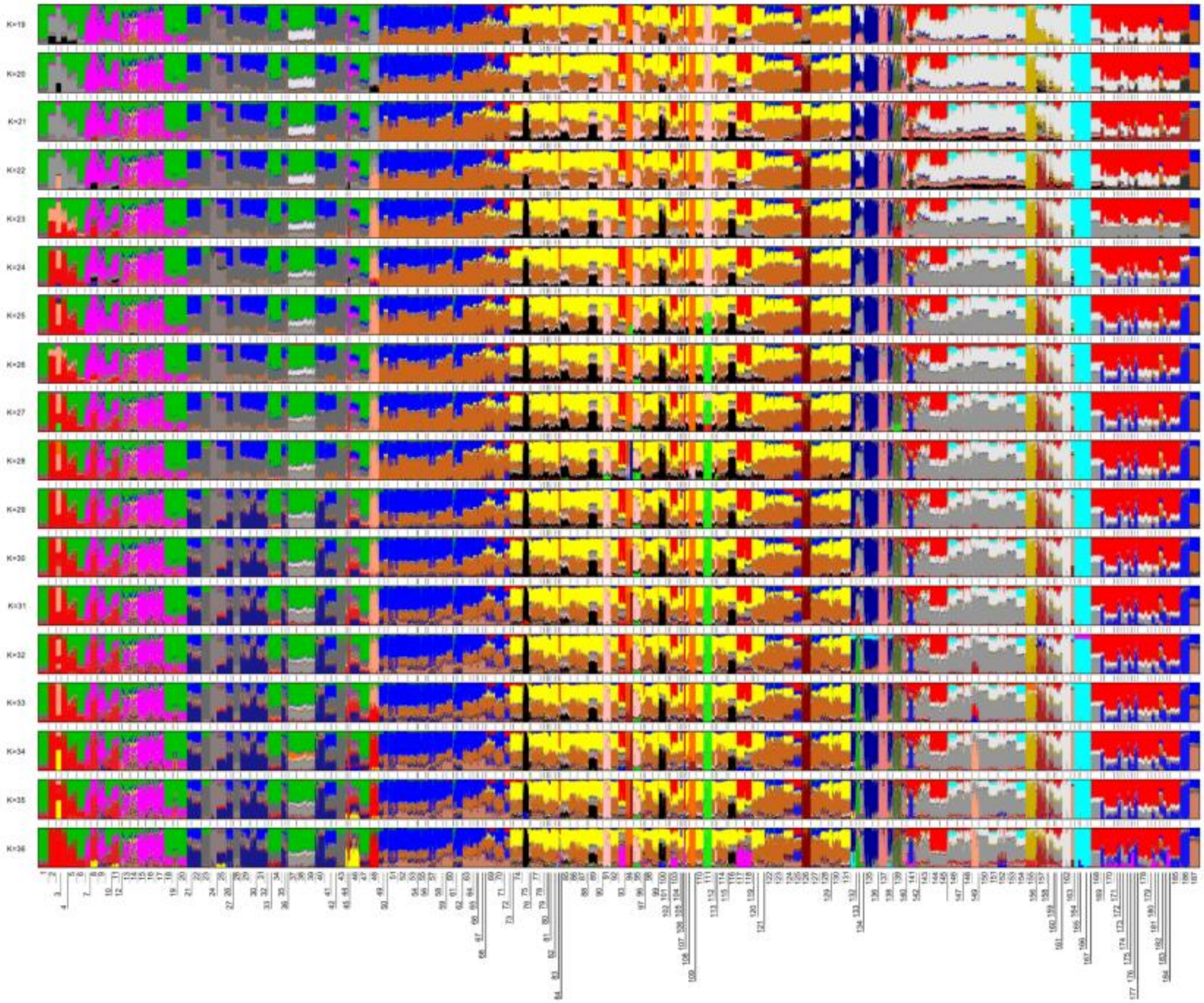
K=19
K=20
K=21
K=22
K=23
K=24
K=25
K=26
K=27
K=28
K=29
K=30
K=31
K=32
K=33
K=34
K=35
K=36

1: West Africa Nigeria Yoruba; 2: Central Africa CAR Biaka Pygmy; 3: Central Africa DR Congo Mbuti Pygmy; 4: Central Africa Gabon Baka; 5: Central Africa Gabon Bongo; 6: Central Africa Gabon Nzebi; 7: South Africa Botswana GuiGhanaKgal; 8: South Africa Namibia Juhoansi; 9: South Africa Namibia Nama; 10: South Africa South Africa/Namibia Khwe; 11: South Africa South Africa/Namibia Xun; 12: South Africa South Africa BantuSA; 13: South Africa South Africa ColouredColesberg; 14: South Africa South Africa ColouredWellington; 15: South Africa South Africa Karretjie; 16: South Africa South Africa Khomani; 17: South Africa South Africa San; 18: South Africa South Africa SEBantu; 19: South Africa South Africa SWBantu; 20: South Africa South African Bantu Soweto; 21: East Africa Ethiopia Afar; 22: East Africa Ethiopia Amhara; 23: East Africa Ethiopia Anuak; 24: East Africa Ethiopia Ari Blacksmith; 25: East Africa Ethiopia Ari Cultivator; 26: East Africa Ethiopia Ethiopian Jews; 27: East Africa Ethiopia Ethiopians; 28: East Africa Ethiopia Gumuz; 29: East Africa Ethiopia Oromo; 30: East Africa Ethiopia Somali; 31: East Africa Ethiopia Tygray; 32: East Africa Ethiopia Wolayta; 33: East Africa Kenya BantuKenya; 34: East Africa Kenya Luhya; 35: East Africa Kenya Samburu; 36: East Africa Kenya Turkana; 37: East Africa Malagasy Mikea; 38: East Africa Malagasy Temoro; 39: East Africa Malagasy Vezo; 40: East Africa Somalia Somali; 41: East Africa Sudan Arab; 42: East Africa Sudan Nubian; 43: East Africa Sudan Sudanese; 44: East Africa Tanzania Hadza; 45: East Africa Tanzania SAN NB; 46: East Africa Tanzania Sandawe; 47: East Africa Uganda Bakiga; 48: East Africa Uganda Batwa; 49: North Africa Egypt Egyptian; 50: Middle East Iraq Iraqi Jews; 51: Middle East Israel Bedouin; 52: Middle East Israel Druze; 53: Middle East Israel Palestinian; 54: Middle East Israel Samaritians; 55: Middle East Jordania Jordanian; 56: Middle East Lebanon Lebanese; 57: Middle East Saudi Arabia Saudi; 58: Middle East Syria Syrians; 59: Middle East Turkey Sephardic Jews; 60: Middle East Turkey Turks; 61: Middle East Yemen Yemeni; 62: Middle East Yemen Yemenite Jews; 63: Caucasus Armenia Armenian; 64: Caucasus Azerbaijan Azerbaijani Jews; 65: Caucasus Georgia Georgian; 66: Caucasus Georgia Georgian Jews; 67: Caucasus Georgia Georgians; 68: Caucasus Russia Lezgin; 69: Central Asia Afghanistan; 70: Central Asia Iran Iranian; 71: Central Asia Iran Iranian Jew; 72: Central Asia Uzbekstan Uzbek; 73: South Asia Bangladesh Bengali; 74: South Asia India Bengali; 75: South Asia India Birhor; 76: South Asia India Bonda; 77: South Asia India Brahmin; 78: South Asia India Brahmins from Uttar Pradesh; 79: South Asia India Chamar; 80: South Asia India Chenchus; 81: South Asia India Cochin Jews; 82: South Asia India Dharkar; 83: South Asia India Dusadh; 84: South Asia India Garo; 85: South Asia India Gond; 86: South Asia India Gujarat Brahmin; 87: South Asia India Gujarati; 88: South Asia India Hakkipikki; 89: South Asia India Ho; 90: South Asia India Indian; 91: South Asia India Irula; 92: South Asia India Iyer; 93: South Asia India Jamatia; 94: South Asia India Jarawa; 95: South Asia India Kadar; 96: South Asia India Kanjars; 97: South Asia India Khasi; 98: South Asia India Khatri; 99: South Asia India Kol; 100: South Asia India Korva; 101: South Asia India Kshatriya; 102: South Asia India Kurumba; 103: South Asia India Manipuri Brahmin; 104: South Asia India Maratha; 105: South Asia India Mumbai Jews; 106: South Asia India Muslim; 107: South Asia India Naga; 108: South Asia India North Kannadi; 109: South Asia India Onge; 110: South Asia India Palian; 111: South Asia India Paniya; 112: South Asia India Piramalai Kallar; 113: South Asia India Pulliyar; 114: South Asia India Punjabi; 115: South Asia India Sakilli; 116: South Asia India Santal; 117: South Asia India Tharu; 118: South Asia India Tripuri; 119: South Asia India Uttar Pradesh Scheduled Caste; 120: South Asia India Velama; 121: South Asia India West Bengal Brahmin; 122: South Asia Pakistan Balochi; 123: South Asia Pakistan Brahui; 124: South Asia Pakistan Burusho; 125: South Asia Pakistan Hazara; 126: South Asia Pakistan Kalash; 127: South Asia Pakistan Makrani; 128: South Asia Pakistan Pathan; 129: South Asia Pakistan Punjabi; 130: South Asia Pakistan Sindhi; 131: South Asia SriLanka Sri Lanka UK; 132: Mainland Southeast Asia Malaysia Bateq; 133: Mainland Southeast Asia Malaysia CheWong; 134: Mainland Southeast Asia Malaysia Jakun; 135: Mainland Southeast Asia Malaysia Jehai; 136: Mainland Southeast Asia Malaysia Kintaq; 137: Mainland Southeast Asia Malaysia MahMeri; 138: Mainland Southeast Asia Malaysia Mendriq; 139: Mainland Southeast Asia Malaysia Seletar; 140: Mainland Southeast Asia Malaysia Temuan; 141: Mainland Southeast Asia Myanmar Burmese; 142: Mainland Southeast Asia Cambodia Cambodian; 143: Mainland Southeast Asia Malaysia Malay; 144: Mainland Southeast Asia Vietnam Kinh; 145: Mainland Southeast Asia Vietnam Vietnamese; 146: Island Southeast Asia Indonesia Bajo; 147: Island Southeast Asia Indonesia Banjar; 148: Island Southeast Asia Indonesia Dusun; 149: Island Southeast Asia Indonesia Lebbo; 150: Island Southeast Asia Indonesia Ma'anyan; 151: Island Southeast Asia Indonesia Mandar; 152: Island Southeast Asia Indonesia Murut; 153: Island Southeast Asia Indonesia SKDayak; 154: Island Southeast Asia Indonesia Sumba; 155: Island Southeast Asia Philippines Aeta; 156: Island Southeast Asia Philippines Agta; 157: Island Southeast Asia Philippines Batak; 158: Island Southeast Asia Philippines Casigurans; 159: Island Southeast Asia Philippines Tagbanua; 160: Island Southeast Asia Philippines Zambales; 161: Island Southeast Asia Phillippines Filipino; 162: Island Southeast Asia Phillippines Igorot; 163: Melanesia Papua New Guinea Bougainville; 164: Melanesia Papua New Guinea Koinambe; 165:

Melanesia Papua New Guinea Kosipe; 166: Melanesia Papua New Guinea 5; 167: Melanesia Papua New Guinea PNG Highlander; 168: East Asia China Dai; 169: East Asia China Daur; 170: East Asia China Han; 171: East Asia China Han NChina; 172: East Asia China Hezhen; 173: East Asia China Lahu; 174: East Asia China Miao; 175: East Asia China Mongolian; 176: East Asia China Naxi; 177: East Asia China Oroqen; 178: East Asia China S Han; 179: East Asia China She; 180: East Asia China Tu; 181: East Asia China Tujia; 182: East Asia China Uygur; 183: East Asia China Xibo; 184: East Asia China Yi; 185: East Asia Japan Japanese; 186: North Asia Russia Yakut; 187: North America USA CEU

Supplementary figure 6 – Box plots of cross-validation values obtained for ADMIXTURE[29] runs from K=20 to K=36 after 10 iterations.

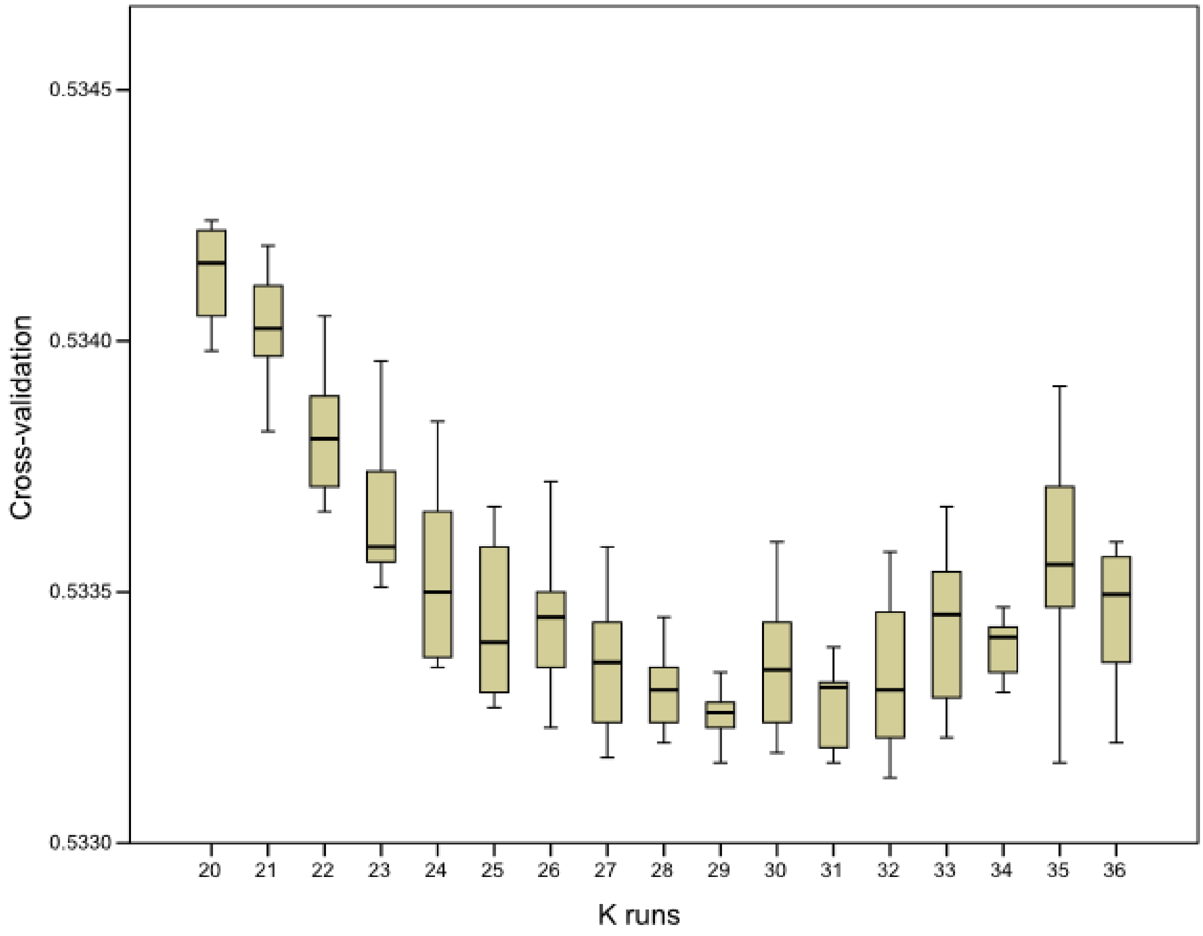

Supplementary figure 7 – Scatterplot showing the estimated dates of admixture using GLOBETROTTER[26] and MALDER[27] for each analysed population. The values are highly correlated (r2=0.65; P<0.00001).

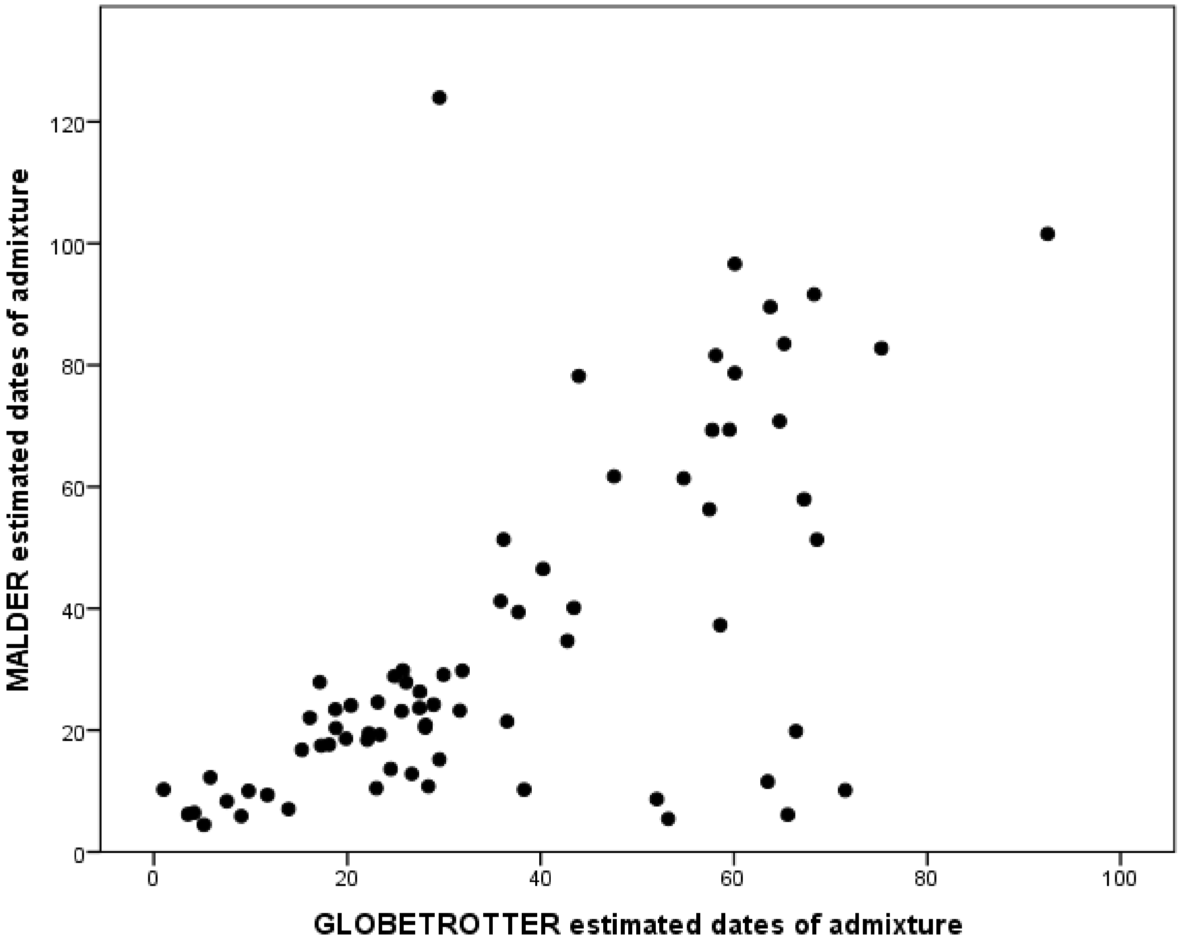

REFERENCES

1 Lawson, D. J., Hellenthal, G., Myers, S. & Falush, D. Inference of population structure using dense haplotype data. *PLoS Genet* **8**, e1002453, doi:10.1371/journal.pgen.1002453 (2012).

2 Patterson, N. J. *et al.* Ancient admixture in human history. *Genetics* **192**, 1065-1093, doi:10.1534/genetics.112.145037 (2012).

3 Patin, E. *et al.* The impact of agricultural emergence on the genetic history of African rainforest hunter-gatherers and agriculturalists. *Nat Commun* **5**, 3163, doi:10.1038/ncomms4163 (2014).

4 Henn, B. M. *et al.* Hunter-gatherer genomic diversity suggests a southern African origin for modern humans. *Proc Natl Acad Sci U S A* **108**, 5154-5162, doi:10.1073/pnas.1017511108 (2011).

5 Schlebusch, C. M. *et al.* Genomic variation in seven Khoe-San groups reveals adaptation and complex African history. *Science* **338**, 374-379, doi:10.1126/science.1227721 (2012).

6 May, A. *et al.* Genetic diversity in black South Africans from Soweto. *BMC Genomics* **14**, 644, doi:10.1186/1471-2164-14-644 (2013).

7 Pierron, D. *et al.* Genome-wide evidence of Austronesian–Bantu admixture and cultural reversion in a hunter-gatherer group of Madagascar. *PNAS* **111**, 936-941, doi:10.1073/pnas.1321860111 (2014).

8 Pagani, L. *et al.* Ethiopian genetic diversity reveals linguistic stratification and complex influences on the Ethiopian gene pool. *Am J Hum Genet* **91**, 83-96, doi:10.1016/j.ajhg.2012.05.015 (2012).

9 Triska, P. *et al.* Extensive Admixture and Selective Pressure Across the Sahel Belt. *Genome biology and evolution* **7**, 3484-3495, doi:10.1093/gbe/evv236 (2015).

10 Behar, D. M. *et al.* The genome-wide structure of the Jewish people. *Nature* **466**, 238-242, doi:10.1038/nature09103 (2010).

11 Basu, A., Sarkar-Roy, N. & Majumder, P. P. Genomic reconstruction of the history of extant populations of India reveals five distinct ancestral components and a complex structure. *Proc Natl Acad Sci U S A* **113**, 1594-1599, doi:10.1073/pnas.1513197113 (2016).

12 Reich, D., Thangaraj, K., Patterson, N., Price, A. L. & Singh, L. Reconstructing Indian population history. *Nature* **461**, 489-494, doi:10.1038/nature08365 (2009).

13 Metspalu, M. *et al.* Shared and unique components of human population structure and genome-wide signals of positive selection in South Asia. *Am J Hum Genet* **89**, 731-744, doi:10.1016/j.ajhg.2011.11.010 (2011).

14 Yunusbayev, B. *et al.* The genetic legacy of the expansion of Turkic-speaking nomads across Eurasia. *PLoS Genet* **11**, e1005068, doi:10.1371/journal.pgen.1005068 (2015).

15 Moorjani, P. *et al.* Genetic evidence for recent population mixture in India. *Am J Hum Genet* **93**, 422-438, doi:10.1016/j.ajhg.2013.07.006 (2013).

16 Chaubey, G. *et al.* Population genetic structure in Indian Austroasiatic speakers: the role of landscape barriers and sex-specific admixture. *Mol Biol Evol* **28**, 1013-1024, doi:10.1093/molbev/msq288 (2011).

17 Kusuma, P. *et al.* Contrasting Linguistic and Genetic Origins of the Asian Source Populations of Malagasy. *Sci Rep* **6**, 26066, doi:10.1038/srep26066 (2016).

18 Brucato, N. *et al.* Malagasy Genetic Ancestry Comes from an Historical Malay Trading Post in Southeast Borneo. *Mol Biol Evol* **33**, 2396-2400, doi:10.1093/molbev/msw117 (2016).

19 Teo, Y. Y. *et al.* Singapore Genome Variation Project: a haplotype map of three Southeast Asian populations. *Genome Res* **19**, 2154-2162, doi:10.1101/gr.095000.109 (2009).

20 Morseburg, A. *et al.* Multi-layered population structure in Island Southeast Asians. *Eur J Hum Genet*, doi:10.1038/ejhg.2016.60 (2016).

21 Migliano, A. B. *et al.* Evolution of the pygmy phenotype: evidence of positive selection fro genome-wide scans in African, Asian, and Melanesian pygmies. *Hum Biol* **85**, 251-284, doi:10.3378/027.085.0313 (2013).

22 Cox, M. P. *et al.* Small Traditional Human Communities Sustain Genomic Diversity over Microgeographic Scales despite Linguistic Isolation. *Mol Biol Evol* **33**, 2273-2284, doi:10.1093/molbev/msw099 (2016).
23 Aghakhanian, F. *et al.* Unravelling the genetic history of Negritos and indigenous populations of Southeast Asia. *Genome biology and evolution* **7**, 1206-1215, doi:10.1093/gbe/evv065 (2015).
24 Xing, J. *et al.* Toward a more uniform sampling of human genetic diversity: a survey of worldwide populations by high-density genotyping. *Genomics* **96**, 199-210, doi:10.1016/j.ygeno.2010.07.004 (2010).
25 Di Cristofaro, J. *et al.* Afghan Hindu Kush: where Eurasian sub-continent gene flows converge. *PLoS One* **8**, e76748, doi:10.1371/journal.pone.0076748 (2013).
26 Hellenthal, G. *et al.* A genetic atlas of human admixture history. *Science* **343**, 747-751, doi:10.1126/science.1243518 (2014).
27 Loh, P. R. *et al.* Inferring admixture histories of human populations using linkage disequilibrium. *Genetics* **193**, 1233-1254, doi:10.1534/genetics.112.147330 (2013).
28 Petkova, D., Novembre, J. & Stephens, M. Visualizing spatial population structure with estimated effective migration surfaces. *Nat Genet* **48**, 94-100, doi:10.1038/ng.3464 (2016).
29 Alexander, D. H., Novembre, J. & Lange, K. Fast model-based estimation of ancestry in unrelated individuals. *Genome Res.* **19**, 1655-1664, doi:10.1101/gr.094052.109 (2009).
30 Kopelman, N. M., Mayzel, J., Jakobsson, M., Rosenberg, N. A. & Mayrose, I. Clumpak: a program for identifying clustering modes and packaging population structure inferences across K. *Mol Ecol Resour* **15**, 1179-1191, doi:10.1111/1755-0998.12387 (2015).
31 Genesis v. 0.2.5 http://www.bioinf.wits.ac.za/software/genesis (2014).
